# Reannotation of cancer mutations based on expressed RNA transcripts reveals functional non-coding mutations in melanoma

**DOI:** 10.1016/j.ajhg.2025.04.005

**Published:** 2025-05-12

**Authors:** Daniele Pepe, Xander Janssens, Kalina Timcheva, Grecia M. Marrón-Liñares, Benno Verbelen, Vasileios Konstantakos, Dylan De Groote, Jolien De Bie, Amber Verhasselt, Barbara Dewaele, Arne Godderis, Charlotte Cools, Mireia Franco-Tolsau, Jonathan Royaert, Jelle Verbeeck, Kim R. Kampen, Karthik Subramanian, David Cabrerizo Granados, Gerben Menschaert, Kim De Keersmaecker

**Affiliations:** 1 Department of Oncology, KU Leuven, Leuven, Belgium; 2 Leuven Cancer Institute (LKI), Leuven, Belgium; 3 Department of Human Genetics, KU Leuven, Leuven, Belgium; 4 VIB Center for AI & Computational Biology (VIB.AI), Leuven, Belgium; 5 VIB-KU Leuven Center for Brain & Disease Research, Leuven, Belgium; 6 Center for Human Genetics, University Hospitals Leuven, Leuven, Belgium; 7 OHMX.bio NV, Evergem, Belgium; 8 Department of Radiation Oncology (Maastro), GROW School for Oncology and Reproduction, Maastricht University Medical Centre, Maastricht, the Netherlands; 9 Department of Data Analysis and Mathematical Modelling, Ghent University, Ghent, Belgium

**Keywords:** synonymous mutations, non-coding mutations, melanoma, functional genomics, CRISPR-Cas9, gene regulation, bioinformatics, mutation annotation, expressed transcript

## Abstract

The role of synonymous mutations in cancer pathogenesis is currently underexplored. We developed a method to detect significant clusters of synonymous and missense mutations in public cancer genomics data. In melanoma, we show that 22% (11/50) of these mutation clusters are misannotated as coding mutations because the reference transcripts used for their annotation are not expressed. Instead, these mutations are actually non-coding. This, for instance, applies to the mutation clusters targeting known cancer genes kinetochore localized astrin (SPAG5) binding protein (*KNSTRN*) and BCL2-like 12 (*BCL2L12*), each affecting 4%–5% of melanoma tumors. For the latter, we show that these mutations are functional non-coding mutations that target the shared promoter region of interferon regulatory factor 3 (*IRF3*) and *BCL2L12*. This results in downregulation of IRF3, BCL2L12, and tumor protein p53 (TP53) expression in a CRISPR-Cas9 primary melanocyte model and in melanoma tumors. In individuals with melanoma, these mutations were also associated with a worse response to immunotherapy. Finally, we propose a simple automated method to more accurately annotate cancer mutations based on expressed transcripts. This work shows the importance of integrating DNA- and RNA-sequencing data to properly annotate mutations and identifies a number of previously overlooked and wrongly annotated functional non-coding mutations in melanoma.

## Graphical abstract

**Figure:**
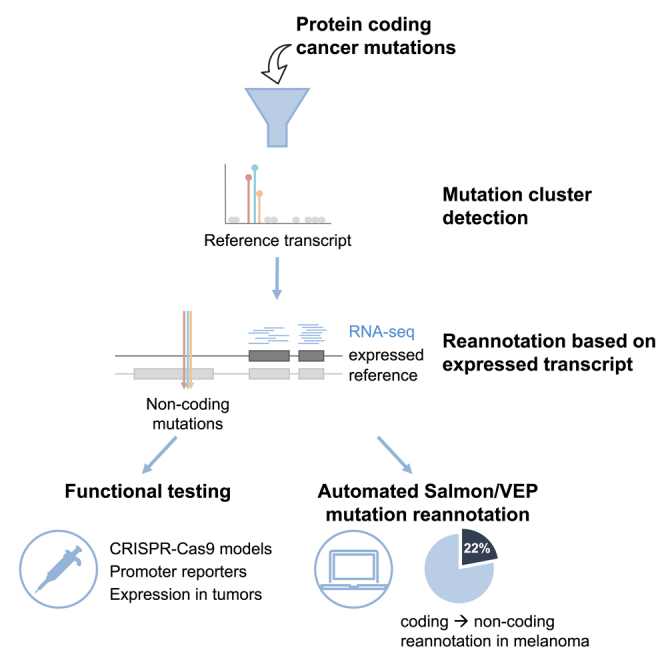

## Teaser

Cancer mutations are often annotated to the reference transcript, which is not always expressed. By annotating to expressed transcripts, we identify a number of overlooked functional non-coding mutations in melanoma, including *IRF3/BCL2L12* promoter mutations. Moreover, we propose a simple automated method for improved mutation annotation based on expressed transcripts.

## Introduction

Next-generation sequencing technologies have revolutionized the detection of mutations in cancer samples. Currently, the majority of clinicians and scientists interpreting cancer sequencing results only consider mutations that alter the amino acid sequence of proteins. Indeed, synonymous mutations are not always reported in cancer genomics databases (e.g., cBioportal). Furthermore, computational biologists frequently use synonymous mutations to calculate random background mutation rates (BMR), based on the assumption that these mutations are biologically insignificant and do not affect tumor development or drug sensitivity.^1^^,^^2^ Nevertheless, there are several examples of synonymous mutations that cause fitness and growth phenotypes in model organisms or that play causative roles in human diseases.^3^^,^^4^^,^^5^ These mutations affect gene-expression levels through various mechanisms, including altered splicing or microRNA (miRNA) binding. They can also modify secondary mRNA structure and, hence, mRNA stability and translation speed. Finally, synonymous mutations can result in codons with altered tRNA availability, affecting translation speed, protein expression, and protein folding.^5^^,^^6^^,^^7^ In cancer, several synonymous mutations change the expression of oncogenes and tumor suppressors. For instance, synonymous mutations in APC regulator of WNT signaling pathway (*APC* [MIM: 611731]), BRCA1/2 DNA repair associated (*BRCA1/2* [MIM: 113705 and 600185]), *TP53* [MIM: 191170], and KRAS proto-oncogene, GTPase (*KRAS* [MIM: 190070]) affect splicing and gene expression.^6^^,^^8^ The recurrent KRAS c.30A>C (GenBank: NM_004985.5) (p.Gly10=) mutation has been reported to affect the secondary structure of the *KRAS* mRNA, causing higher KRAS oncogene protein expression.^9^ In melanoma, a synonymous c.51C>T (GenBank: NM_001282520.1) (p.Phe17=) mutation in the *BCL2L12* (MIM: 610837) oncogene was shown to impair binding of miRNA hsa-miR-671-5p to the *BCL2L12* mRNA, leading to BCL2L12 overexpression and cellular resistance to UV-induced apoptosis.^10^ These examples illustrate rare experimentally tested synonymous mutations that can affect the expression of key proteins in cancer pathogenesis.

*In silico* approaches have been developed to systematically annotate synonymous mutations in cancer and identify candidate pathogenic mutations across entire cancer exomes.^9^^,^^11^^,^^12^ However, previous studies suffer from several limitations that prevent a comprehensive view on the landscape of candidate pathogenic synonymous mutations. First, mutations in known cancer genes were often prioritized, preventing discovery of relevant mutations in genes that have not previously been linked to cancer. Second, synonymous mutations were typically analyzed as a separate mutation category even though synonymous and missense nucleotide changes can affect the same molecular mechanisms as splicing, altered secondary RNA structure, and so forth. By focusing only on synonymous mutations, missense mutations that co-cluster with synonymous mutation remain undetected, and the statistical power of detecting mutations with evidence for strong positive selection is reduced. Additionally, missense mutations with a relevant role in cancer pathogenesis may have been overlooked, as predictions on their functional impact rarely account for effects on other gene-regulatory mechanisms apart from amino acid changes. Third, most studies on synonymous mutations limit their analyses to DNA-sequencing (DNA-seq) data without considering the impact of the mutation on gene-expression levels or whether the mutation is located in an expressed RNA transcript. Therefore, we developed an approach that overcomes these limitations to investigate the landscape of candidate pathogenic synonymous and missense mutations in cancer genomics data. Application of our method on The Cancer Genome Atlas (TCGA) data identified a number of previously underappreciated clusters of mutations that are annotated as synonymous and missense, in particular in melanoma. Intriguingly, in respect to the transcripts that are expressed in melanoma, 22% (11/50) of the identified mutational clusters corresponded to non-coding rather than synonymous or missense mutations. This includes previously characterized mutations that were formerly annotated as KNSTRN c.71C>T (GenBank: NM_033286.4) (p.Ser24Phe) and BCL2L12 (p.Phe17=). For the mutations targeting the *BCL2L12* locus, we generated isogenic CRISPR-Cas9 models in which we show that these mutations correspond to functional *IRF3/BCL2L12* promoter mutations that cause downregulation of IRF3, BCL2L12, and downstream TP53. These results were also confirmed in melanoma tumor samples, and the mutations are associated with a worse response to immunotherapy in individuals with melanoma. Also for other identified non-coding mutations, effects on gene-promoter activity and/or expression of their host gene support their functionality. Finally, we propose a simple automated method based on the freely available tools Salmon^13^ and Ensembl Variant Effect Predictor (VEP)^14^ to annotate cancer mutations in respect to the transcript that is expressed, avoiding misannotation of mutations. To conclude, our results underscore the limitations of cancer genomics databases relying on reference transcript annotations, as these may not reflect the transcripts expressed in a given tumor type. This work makes a major contribution to the cancer genomics field by showing the importance of integrating DNA- and RNA-sequencing data to properly annotate cancer mutations and by identifying a number of previously overlooked functional non-coding mutations in melanoma.

## Material and methods

### Public cancer genomics data

The research on human cancer genomics datasets described in this paper was performed in accordance with the ethical standards of the responsible committee on human experimentation (Ethics Committee Research UZ/KU Leuven; approval S63550), and proper informed consent was obtained. High-confidence somatic mutation calls of TCGA cancer genomics data were used from the MC3 working group.^15^ TCGA mRNA expression data were downloaded from https://gdc.cancer.gov/about-data/publications/pancanatlas. RNA-sequencing (RNA-seq) BAM files were downloaded from NCI Genomics Data Commons after having obtained dbGaP authorized access to dataset phs003014 (NCI’s Datasets for General Research Use, study accession phs000178.v11.p8—TCGA). DNA-seq data from skin tumors available from the Catalog of Somatic Mutations in Cancer (COSMIC v.101; consulted on November 22, 2024) were also utilized for this study. To check the association of mutations with response on immunotherapy, we utilized the aggregated whole-exome sequencing data from individuals receiving immunotherapy with matched Response Evaluation Criteria in Solid Tumors (RECIST) from six previously published studies, which was compiled by Gajic et al.^16^ DNase sequencing (DNase-seq) and chromatin immunoprecipitation sequencing (ChIP-seq) bigwig files were downloaded from ENCODE.

### Mutational concentration analysis

This analysis is also summarized in Figure S1.

#### Pre-processing of MC3 variant lists

MC3 working group somatic TCGA variant calls were downloaded from https://gdc.cancer.gov/about-data/publications/mc3-2017.^15^ Only PASS FILTER single-nucleotide variants (SNVs) with a protein coding annotation (missense, synonymous, stop lost, stop gained, or coding sequence variant) were kept. Variants in immunoglobulin, HLA, T cell receptor, and olfactory genes were removed. Variant calls in non-expressed genes were removed, with non-expressed genes defined as genes with an RNA expression FPKM value (fragments per kilobase of transcript per million mapped reads) lower than 2 for at least 80% of tumor samples in the entire dataset as proposed by others.^17^ All mutations detected in hypermutated tumors (mutation load below Q1 − 3 × interquartile range (IQR) or above Q3 + 3 × IQR for the analyzed tumor type) were discarded. Finally, all variants with a variant allele fraction (VAF) <0.2 were also discarded.

#### Mutational concentration methods

**Figure 1:**
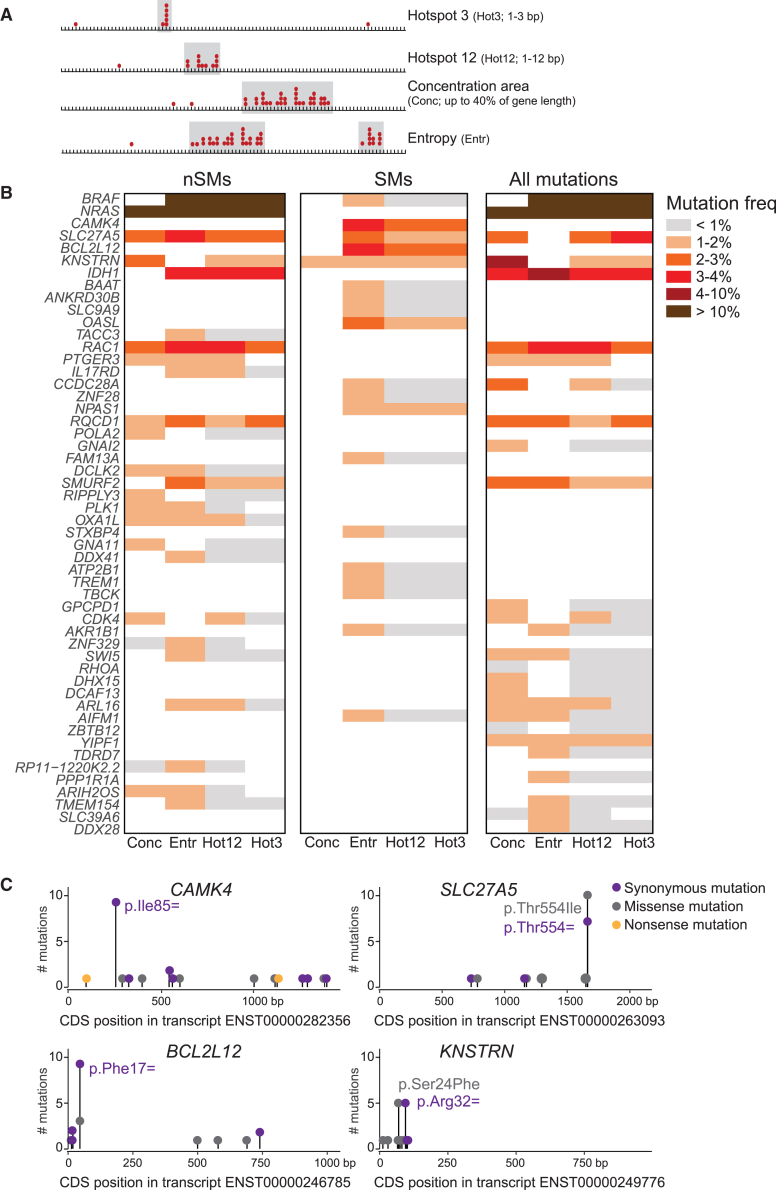
Significant mutation clusters in melanoma (SKCM) (A) Image explaining the four different concentration detection methods that were applied in this study. (B) Heatmap summarizing significant mutation clusters in SKCM detected using four mutational detection methods (Conc, concentration method; Entr, entropy method; Hot12, hotspot 12 method; Hot3, hotspot 3 method). The heatmap depicts results obtained when only considering non-synonymous missense and nonsense mutations (nSMs, left), when only considering synonymous mutations (SMs, middle), and when considering non-synonymous and synonymous mutations together (All mutations, right). Only genes that were significant for at least three concentration methods for nSMs, SMs, or All mutations are depicted in this heatmap. All genes with significant mutation clusters are reported in Tables S2, S3, S4, and S5. The genes in the heatmap are ranked according to mutation frequency, and detected significant mutation clusters are colored according to the frequency of mutations in the identified clusters in the SKCM dataset as explained in the legend on the right. (C) Needleplots illustrating the distribution of identified mutations in *CAMK4*, *SLC27A5*, *BCL2L12*, and *KNSTRN*.

On the obtained filtered MC3 variant list, four complementary approaches were applied to identify genes displaying significant clustering of mutations in restricted areas (Figure 1A). For the hotspot 3 method (hotspots of three contiguous nucleotides), the method developed by Chang et al. was applied.^18^ A gene scored positive in the hotspot 12 method if at least five mutations were detected and if at least 40% of all gene mutations cluster in a window of 12 contiguous nucleotides. For the mutational concentration method, genes with a minimum of five mutations and clustering of at least 70% of all gene mutations in maximum 40% of the transcript length were retained. Finally, for the entropy method, we calculated the Shannon entropy score.^19^^,^^20^ Genes with a minimum of five mutations and maximal entropy value of 0.2 were retained. For the hotspot 12, mutational concentration, and entropy methods, a permutation test was performed to evaluate significance. For this permutation test, the number of detected mutations in the transcript were 10,000 times assigned to random positions in the transcript, and we counted how many times the mutations clustered in a region of ≤12 nucleotides (Hotspot 12), in a region ≤40% of the transcript length (Concentration), or how many times the entropy score was <0.2 (Entropy). The permutation test *p* value was calculated as follows (number of positive results + 1)/(10,000 + 1).^21^ A *p* value threshold of 0.05 was utilized to evaluate significance. These four methods were applied on the list of filtered variants from all 17 tumor types with more than 200 tumors (Table S1). Only genes that were expressed in the analyzed tumor type were retained (cutoff FPKM >1 in at least 80% of the samples). Obtained results from the four concentration methods are reported in Tables S2, S3, S4, and S5. To further filter these results for representation in heatmaps, only genes detected by at least two of the above described methods in the analyzed tumor type were retained. For tumors with more than 100 genes detected by two methods (skin cutaneous melanoma [SKCM]), only genes that were significant in three concentration methods were retained for the heatmap (Figures 1B and S2; Table S6).

### Cell culture

Mel-ST cells were kindly provided by Robert A. Weinberg.^22^ Genetic analysis and engineering of these cells were approved by the Ethics Committee Research UZ/KU Leuven (S66987). HEK-293T cells (ACC 635) were obtained from the German Collection of Microorganisms and Cell Cultures (DSMZ). Mel-ST and HEK-293T cells were grown in DMEM (#41965039, Thermo Fisher Scientific) supplemented with 10% fetal bovine serum (#1017760, Thermo Fisher Scientific) in a humidified cell-culture incubator at 37°C with 5% CO_2_. Cells were regularly verified to be mycoplasma free.

### CRISPR genome editing

The CRISPR-Cas9 genome editing design and generation of the mutant Mel-ST cell lines was performed by genOway (Lyon, France). In brief, cells were cultivated at subconfluence and electroporated with CRISPR reagents (Cas9 nuclease, guide RNA, and single-stranded donor oligodeoxynucleotide [ssODN]) after cell dissociation, using a Neon transfection system (Thermo Fisher) and following the manufacturer’s guidelines. The used guide RNA sequences were CCAAAAAAGGGCATAGGGGC (for g.49665847C>T [GenBank: NC_000019.10] mutation) and GCTCCCAGCATGCCTCTGGC (for g.49665874C>T [GenBank: NC_000019.10] and g.49665875C>T [GenBank: NC_000019.10] mutations). The ssODN was designed to only introduce the desired point mutation without introducing additional mutations to disrupt the protospacer adjacent motif (PAM) site. Electroporated cells were single-cell sorted and expanded to obtain pure clonal populations. DNA from sorted clones was extracted and characterized by PCR and Sanger sequencing of the targeted locus. The absence of mutations at predicted off-target sites was verified by Sanger sequencing, and diploid copy number of the targeted locus was verified by qPCR.

### RT-qPCR

RNA was extracted using RNeasy Mini Kit (#74106, Qiagen), and 500 ng was used as input for cDNA synthesis with GoScript Reverse Transcriptase, Random Primers, dNTP Mix, and RNasin Ribonuclease Inhibitors (#A5004, #C1181, #U1511, #N2511; Promega). RT-qPCR was performed on a QuantStudio 5 instrument (Thermo Fisher) and analyzed using the ΔΔCt method. Primer sequences are reported in Table S10.

### Western blot

Protein electrophoresis and western blotting were performed using the Bio-Rad Criterion system according to manufacturer’s guidelines with 40 μg of total loaded protein. Enhanced chemiluminescence-based western blot detections were performed using standard procedures using the antibodies specified in Table S11. Proteins were visualized on an Azure c600 (Azure Biosystems) and quantified using Image Studio Lite v.5.2 (LI-COR Biotech).

### shRNA transduction

Lentiviral plasmids containing VSV.G (Addgene), psPAX2 (Addgene), and BCL2L12 short hairpin RNA (shRNA) constructs (Table S10) with a GFP reporter (TL306421, Origene) were transiently transfected into HEK293-T cells using JetOPTIMUS according to the manufacturer’s instructions. Culture medium containing viral particles was used to stably transduce Mel-ST cells in presence of 16 μg/mL polybrene infection reagent (Merck). Transduction efficiencies of 95% or more were reached in all conditions.

### *In silico* transcription factor binding analysis

For scoring non-coding variants for impact on transcription factor binding, we applied DeepMEL2, which was trained on *cis*-regulatory topics (sets of co-accessible genomic regions clustered by cisTopic^23^) from 30 melanoma lines and was shown to accurately predict the accessibility and activity of a sequence in different melanoma subtypes.^24^ Here, we focused on Topic 14, which was ubiquitously active (General), and Topics 16 and 36, which were associated with the melanocytic (MEL) and mesenchymal (MES) subtype, respectively. In addition, we used model interpretability techniques such as DeepExplainer (DeepSHAP)^25^ to calculate the contribution of each nucleotide to the final prediction of the model for the chosen topic. We used 250 randomly selected genomic regions to initialize the explainer with a background distribution. For a given 500-bp input, we multiplied the explainer’s output (averaged over the forward and reverse strand) by the one-hot encoded DNA sequence and visualized it as the height of the nucleotide letters. The altered motifs were manually annotated to demonstrate the disruption or creation of transcription factor binding sites that guided the model predictions. We also applied FABIAN-variant,^26^ which utilizes transcription factor flexible models based on hidden Markov models and position weight matrices to predict the impact of DNA variants on transcription factor binding. FABIAN-variant was applied to GRCh38 including 67 known transcription factor binding sites based on ENCODE, Ensembl, and FANTOM5 data and filtered on the JASPAR2022 database. Finally, we utilized PhysBinder,^27^ which is based on random forest machine learning to analyze models for the binding of 75 transcription factors with direct evidence in *Homo sapiens*. The PhysBinder threshold was set to average (PPV and F-measure), and the recommended position-specific scoring matrix filter was applied.

### Luciferase reporter assays

The luciferase reporter assay was performed using the Dual-Luciferase Reporter Assay System (E1910, Promega) according to manufacturer’s guidelines. In brief, HEK-293T cells were transfected with the dual-luciferase reporter plasmids (maps are available in Figure S25, and sequences of *BCL2L12* promoter reporter assays are listed in Figure S11) in a 48-well plate using Lipofectamine 3000 (#L3000001, Thermo Fisher Scientific). After 24 h the cells were lysed, and firefly and *Renilla* luciferase expression was measured consecutively using the Dual-Luciferase Reporter Assay System (E1910, Promega) on a Victor X4 multilabel microplate reader (PerkinElmer). Background signal was subtracted from luciferase signals before calculating the relative firefly/*Renilla* ratio and normalizing to the wild-type reporter.

### Salmon/VEP automated annotation of mutations

To identify the main expressed transcript for genes of interest in a particular tumor type, transcriptomic BAM files from 35 human TCGA tumors were downloaded from dbGaP. Salmon^13^ was applied to quantify the different expressed transcript isoforms per gene in each sample, and results obtained from the 35 samples were averaged to identify the main expressed transcript per analyzed gene in the tumor type of interest. Next, Ensembl VEP^14^ was used to annotate mutations of interest, and VEP results were filtered to only retain the annotation in respect to the main expressed transcript identified by Salmon.

### Nanopore sequencing isoform analysis

RNA from three replicates of Mel-ST cells taken on different days was extracted using an RNeasy Mini Kit (#74106, Qiagen). To convert RNA fragments into cDNA and perform library preparation, the Direct cDNA Sequencing Kit (#SQK-LSK114, Oxford Nanopore Technologies) in combination with the Native Barcoding Expansion Kit (#SQK-NBD114.96, Oxford Nanopore Technologies) were used. The library pool was sequenced on a PromethION flow cell (#FLO-PRO114M, Oxford Nanopore Technologies). Obtained sequencing reads were basecalled and demultiplexed using the high-accuracy basecall model of Dorado. After removal of reads with a PHRED score below 9, a total number of 26.38 million reads were retained for downstream analysis (≥7.2 million reads per replicate). Isoform detection, quantification, and *de novo* transcript detection was performed using FLAIR2.^28^ The raw data were mapped to the human genome using Minimap2 through the FLAIR align module. Misaligned splice sites were corrected with FLAIR correct using the genome annotation from GENCODE release 47. Lastly, FLAIR collapse was used to define high-confidence isoforms from the corrected reads. The output from the FLAIR correct step was concatenated to create GTF files containing the detected transcript isoforms. A count matrix of the merged samples was also generated to identify the main expressed isoform identified by FLAIR. *De novo* transcripts identified by FLAIR were only considered when no known GENCODE v.47 transcript was identified. Finally, to verify obtained FLAIR results, BAM files of the nanopore sequencing data were loaded into the Integrative Genomics Viewer (IGV), and the identity of the main expressed isoform identified by FLAIR was corrected based on manual IGV inspection where needed.

### Impact of mutations on RNA expression

To analyze whether mutations in an identified mutation cluster are associated with a significant difference in expression of the mutated gene (for coding mutations) or of the gene that is closest by (for non-coding mutations) in SKCM, the SKCM samples were divided into two groups.

(1) Samples containing mutations in the mutational concentration area of interest (referred to as “mutated samples” below). All samples containing a mutation at the most recurrently mutated position in the mutation cluster, as well as all samples with mutations in the region ranging from 30 nt upstream of this position to 30 nt downstream, were included in this group.
(2) Samples lacking mutations in the concentration area (“wild-type samples”). All samples lacking mutations in the mutated gene of interest (coding mutations) or in the gene that is closest by (for non-coding mutations) were assigned to this group.

Samples containing mutations in the gene of interest that are located outside of the mutational concentration area were excluded. For each analyzed mutation cluster, this group assignment in wild-type samples, mutated samples, and excluded samples was repeated. To analyze impact on RNA expression, raw RNA-seq counts were obtained from TCGAbiolinks.^29^ Next, DESeq2 was used to analyze differential mRNA expression between the mutated samples group versus wild-type samples group.

## Results

### Melanoma displays the highest number of significant mutation clusters

To analyze the landscape of synonymous and missense mutations in cancer, we started from the accurate mutation calls of TCGA cancer data generated by the MC3 working group.^15^ This dataset contains somatic mutation calls from 10,437 tumors representing 33 different tumor types. For 8,170 of these samples, RNA-seq data are also available. To avoid inclusion of low-confidence and subclonal variants in our downstream analyses, a thorough filtering of the MC3 dataset was performed (specified in material and methods and Figure S1).

A common approach to identify cancer driver mutations is based on determining whether the mutation frequency of a gene or region is significantly higher than the random background mutation rate (BMR). However, most algorithms utilizing this principle cannot be applied to synonymous mutations, as they use synonymous mutations to estimate the BMR.^1^^,^^2^ Estimating BMR is challenging, so we utilized the concentration of mutations in restricted gene areas as an indicator of positive selection. Four complementary methods were applied to evaluate different patterns of mutational concentration in genes (Figure 1A). First, mutations can cluster in one codon or region of three contiguous nucleotides. To detect such events, we applied a previously developed method (hotspot 3 method, Hot3).^18^ Second, mutations can accumulate in regions of 6–12 nucleotides such as in transcription factor or miRNA binding sites in the gene body. Therefore, we screened for mutational hotspots in nucleotide stretches of up to 12 nt (hotspot 12 method, Hot12). Third, even longer functional DNA or RNA elements of at least 200 bp such as CpG islands or long non-coding RNAs (lncRNAs) may be disrupted by mutations. To identify mutational concentration across such larger gene areas, we identified genes with significant mutation clustering within up to 40% of the length of the coding sequence of a transcript (concentration method, Conc). Finally, some genes may contain multiple hypermutated regions. For example, notch receptor 1 (*NOTCH1* [MIM: 190198]) displays two distinct mutational concentration areas in the gene regions encoding the heterodimerization and the PEST domain in T cell acute lymphoblastic leukemia.^30^ To identify multiple concentrated regions of mutations within one gene, we also applied a fourth, entropy-based, method (entropy method, Entr).

Since synonymous and non-synonymous nucleotide changes can dysregulate similar gene-expression regulatory mechanisms (DNA methylation, splicing, secondary RNA structure, etc.), we reasoned that the power of detecting gene regions with significant clustering of cancer driver mutations is enhanced by analyzing both mutation types together. Therefore, the four mutational concentration analyses were applied on non-synonymous mutations (nSMs), on synonymous mutations (SMs), and on non-synonymous and synonymous mutations together (all mutations). Our methodologies were applied on DNA-seq data from the 17 tumor types from which data from more than 200 samples were available (Table S1). The highest number of genes with significant concentrations of mutations were detected in melanoma (SKCM) (Figures 1B and S2; Tables S2, S3, S4, S5, and S6; Figure S2). Whereas SKCM is known to have the highest overall mutational burden,^31^ our methodology only retains mutations that cluster in restricted gene areas. As such, our method enriches for mutations that have been positively selected in the tumor and hence may exert a driver role in cancer pathogenesis. We re-identified well-established oncogenes in SKCM, such as B-Raf proto-oncogene, serine/threonine kinase (*BRAF* [MIM: 164757]) and NRAS proto-oncogene, GTPase (*NRAS* [MIM: 164790]) and also detected the previously described synonymous mutation hotspot in *BCL2L12*.^10^ Interestingly, our analysis also identified synonymous mutation concentration regions in genes that have not previously been implicated in melanoma, such as calcium/calmodulin-dependent protein kinase IV (*CAMK4* [MIM: 114080]), solute carrier family 27 member 5 (*SLC27A5* [MIM: 603314]), and *KNSTRN* (MIM: 614718) (Figure 1C). These results highlight the potential of our methodology to identify candidate cancer genes.

### Reannotation of synonymous *BCL2L12* mutations in melanoma as non-coding *IRF3/BCL2L12* promoter mutations

**Figure 2:**
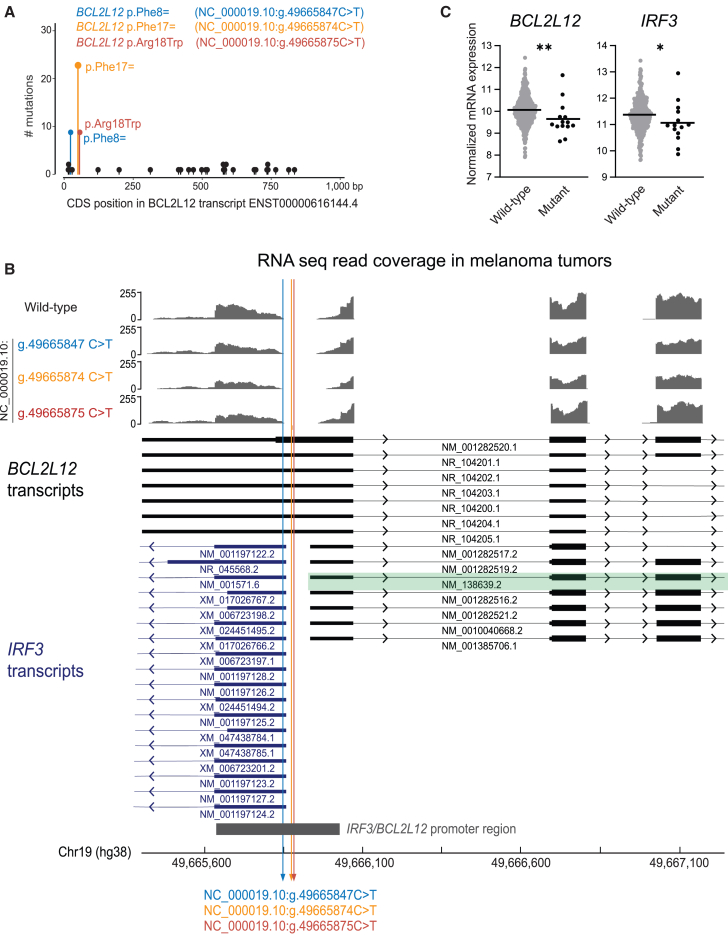
Reannotation of *BCL2L12* mutations in SKCM as functional *IRF3/BCL2L12* promoter mutations (A) Needleplot of COSMIC mutations in *BCL2L12* in malignant melanoma in respect to transcript ENST00000616144.4, the default transcript shown in COSMIC. (B) RNA-seq data from melanoma tumors were mapped against hg38 in the Integrative Genomics Viewer (IGV). A representative RNA-seq read distribution plot of a wild-type and three mutant tumors is shown in the upper part of the panel. The genomic location of the *IRF3/BCL2L12* promoter variants is indicated, as well as the reported transcripts for *BCL2L12* (black) and *IRF3* (blue) in NCBI. Transcript GenBank: NM_138639.2 is highlighted in green, as it was identified as main expressed BCL2L12 transcript, as can be seen in Figure S3. (C) Normalized *BCL2L12* and *IRF3* mRNA expression in melanoma tumors with a wild-type (*n* = 420) or mutant (g.49665874C>T [GenBank: NC_000019.10], g.49665847C>T [GenBank: NC_000019.10], or g.49665875C>T [GenBank: NC_000019.10] mutation; *n* = 14) *IRF3/BCL2L12* promoter region. Horizontal lines indicate median expression. Statistics calculated by Mann-Whitney U test: ^∗^*p* < 0.05, ^∗∗^*p* < 0.01.

Our results indicated that the previously described BCL2L12 p.Phe17= mutation^10^ is part of a formerly unrecognized cluster of mutations (Figure 1C). To better characterize the cluster, we analyzed *BCL2L12* mutation load in 1,076 melanoma samples in the catalog of somatic mutations in cancer (COSMIC) (Figure 2A). This analysis confirmed the BCL2L12 p.Phe17= mutation hotspot (g.49665874C>T [GenBank: NC_000019.10]) in *BCL2L12* transcript ENST00000616144.4 and revealed two additional recurrent mutation hotspots: one upstream point mutation (g.49665847C>T [GenBank: NC_000019.10]) corresponding to a synonymous c.24C>T (GenBank: NM_001282520.1) (p.Phe8=) mutation and one downstream point mutation (g.49665875C>T [GenBank: NC_000019.10]) corresponding to a c.52C>T (GenBank: NM_001282520.1) (p.Arg18Trp) missense mutation in this transcript.

Important to note when describing these mutations is the variety of *BCL2L12* transcripts. Currently, 19 *BCL2L12* transcripts are reported in Ensembl (GRCh38.p13) and 14 are reported in NCBI and UCSC (GRCh38/hg38) (Table S7). Interestingly, depending on which of these transcripts is expressed, the above described variants correspond to synonymous *BCL2L12* mutations, mutations targeting the *BCL2L12* 5′ UTR, or upstream non-coding mutations (Figure 2B and Table S7). To determine which transcripts are expressed in melanoma, we mapped public RNA-seq data from melanoma tumors that are wild-type for the mutations and tumors with the g.49665847C>T (GenBank: NC_000019.10), g.49665874C>T (GenBank: NC_000019.10), or g.49665875C>T (GenBank: NC_000019.10) mutations to the genome. Analysis of the read distribution supported that the expressed *BCL2L12* transcripts corresponded to isoform GenBank: NM_138639.2, for which the identified mutations are upstream non-coding variants (Figures 2B and S3). Additionally, the mutations are located close to the expressed *IRF3* (MIM: 603734) and target the shared promoter region of *IRF3* and *BCL2L12*,^21^^,^^22^ with the g.49665847 C>T (GenBank: NC_000019.10) variant overlapping with the 5′ UTR of the expressed *IRF3* transcripts. In conclusion, we show that the chr19:49665874C>T (formerly described as BCL2L12 p.Phe17=), g.49665847C>T (GenBank: NC_000019.10) (p.Phe8=), and g.49665875C>T (GenBank: NC_000019.10) (p.Arg18Trp) variants correspond to non-coding *IRF3/BCL2L12* promoter mutations, which are detected in 3.8%–5.5% of melanoma tumors (41/1,076 in COSMIC and 26/470 in TCGA-SKCM).

### *IRF3/BCL2L12* promoter mutations reduce *IRF3*, *BCL2L12*, and *TP53* expression and are associated with a worse response to immunotherapy in individuals with SKCM

**Figure 3:**
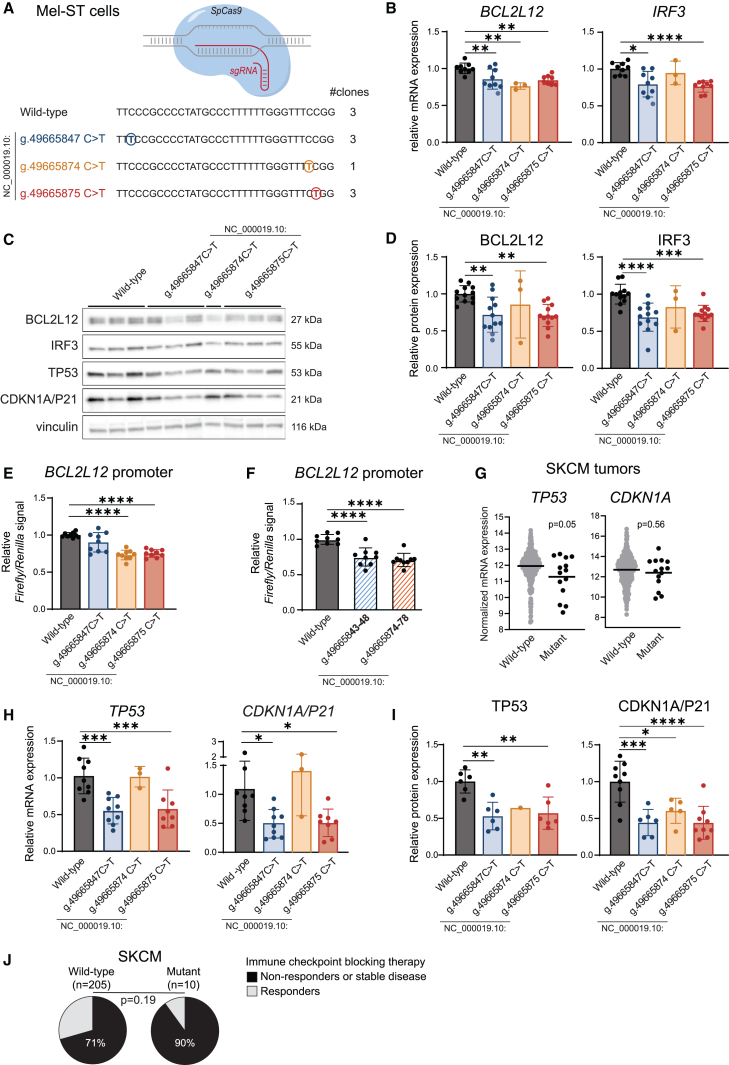
*IRF3/BCL2L12* promoter mutations are functional (A) Overview of knocked-in mutations in Mel-ST cells using CRISPR-Cas9. No additional nucleotide changes besides the indicated mutations were inserted. We obtained three independent single-cell-derived Mel-ST clones for genotypes wild-type, g.49665847C>T (GenBank: NC_000019.10), and g.49665875C>T (GenBank: NC_000019.10) mutations, whereas only one clone could be obtained containing the g.49665874C>T (GenBank: NC_000019.10) variant. All clones were used in each independent experiment. (B) mRNA levels of *BCL2L12* and *IRF3* in Mel-ST cells as measured via RT-qPCR. Expression was normalized to *GAPDH* using the ΔΔCt method. Combined data of three independent experiments. Statistics calculated by ordinary one-way ANOVA (*BCL2L12*) or Brown-Forsythe and Welch ANOVA (*IRF3*). (C) Representative western blot of BCL2L12, IRF3, TP53, CDKN1A, and vinculin in Mel-ST cell clones. Each lane corresponds to sample from an independent single-cell-derived Mel-ST clone of the indicated genotype. Vinculin signal was used to normalize for protein input. (D) Quantified levels of BCL2L12 and IRF3 Mel-ST cells as measured via western blot. Combined data of three independent experiments. Statistics calculated by ordinary one-way ANOVA. (E) Ratio of firefly luciferase signal (under BCL2L12 promoter) over *Renilla* luciferase signal (under constitutive TK promoter) as a measurement of wild-type and mutant *BCL2L12* promoter activity in reporter assays in HEK293T cells. Combined data of three independent experiments. Statistics calculated by Brown-Forsythe and Welch ANOVA (*BCL2L12*). (F) Ratio of firefly luciferase signal (under *BCL2L12* promoter) over *Renilla* luciferase signal (under constitutive TK promoter) to evaluate *BCL2L12* promoter activity in HEK293T cells. g.49665843-48 (GenBank: NC_000019.10) and g.49665874-78 (GenBank: NC_000019.10) correspond to mutated reporters that abolish PhysBinder predicted binding of transcription factors in the corresponding wild-type sequences (sequences shown in Figure S11). Combined data of three independent experiments. Statistics calculated by Brown-Forsythe and Welch ANOVA (*BCL2L12*). (G) Normalized *TP53* and *CDNK1A* mRNA expression in SKCM tumors with a wild-type (*n* = 420) or mutant (g.49665874C>T [NC000019.10], g.49665847C>T [NC000019.10], or g.49665875 C>T [NC000019.10] mutation; *n* = 14) *IRF3/BCL2L12* promoter region. Horizontal lines indicate median expression. Statistics calculated by Mann-Whitney U test. (H) mRNA levels of *TP53* and *CDKN1A* in Mel-ST cells as measured via RT-qPCR. Expression was normalized to *GAPDH* using the ΔΔCt method. Combined data of three independent experiments. Statistics calculated by ordinary one-way ANOVA. (I) TP53 and CDKN1A levels in Mel-ST cells as measured via western blot. Combined data of two (TP53) or three (CDKN1A) independent experiments. Statistics calculated by ordinary one-way ANOVA. (J) Response to immune-checkpoint therapy in individuals with melanoma with a wild-type or mutant *IRF3/BCL2L12* promoter status in the tumor. Statistics calculated by chi-square test. Error bars in (B), (D), (E), (F), (H), and (I) indicate standard deviations. ^∗^*p* ≤ 0.05, ^∗∗^*p* < 0.01, ^∗∗∗^*p* < 0.001, ^∗∗∗∗^*p* < 0.0001.

To assess the functionality of the identified *IRF3/BCL2L12* promoter mutations, we evaluated their impact on *IRF3* and *BCL2L12* mRNA expression in melanoma tumor samples (Figure 2C). Interestingly, *IRF3* and *BCL2L12* mRNA expression was significantly reduced in melanoma tumors with an *IRF3/BCL2L12* promoter mutation as compared to tumors with a wild-type promoter region. Next, we aimed to validate our observations in an experimental model and further elucidate the functional impact of these mutations on melanoma pathogenesis. For this we chose Mel-ST cells,^22^ which are immortalized primary melanocytes offering a suitable pre-malignant model. Illumina-based RNA-seq as well as direct cDNA long-read nanopore sequencing confirmed that Mel-ST cells express the same *IRF3* and *BCL2L12* isoforms as we had observed in melanoma tumors (Figures S4 and S5). Expression of GenBank: NM_138639.2 as main expressed *BCL2L12* isoform was also confirmed at protein level (Figure S6). Next, the three *IRF3/BCL2L12* promoter mutations that we had identified in melanoma were each introduced heterozygously and without additional mutations into Mel-ST cells using CRISPR-Cas9 (Figures 3A and S7). We obtained three independent single-cell-derived Mel-ST clones for the g.49665847C>T (GenBank: NC_000019.10) and g.49665875C>T (GenBank: NC_000019.10) mutations, whereas only one clone could be obtained containing the g.49665874C>T (GenBank: NC_000019.10) variant. In these isogenic models, we confirmed that the *IRF3/BCL2L12* promoter mutations resulted in reduction of *IRF3* and *BCL2L12* mRNA levels by 20% and 16%, respectively (Figure 3B), and a 27% and 21% reduction of IRF3 and of BCL2L12 protein levels (Figures 3C, 3D, and S8).

We then applied DeepMEL2,^24^ PhysBinder,^27^ and FABIAN-variant^26^ to predict the mutational impact on promoter and enhancer function and on transcription factor binding. These different tools consistently predict disruption of transcription factor binding sites for ETS family members, in particular ETS1, ELK1, ELK4, ELF1 ELF4, and GA binding protein transcription factor subunit alpha (GABPA), for the g.49665874C>T (GenBank: NC_000019.10) and g.49665875C>T (GenBank: NC_000019.10) mutations (Figure S9). For ELK1 and ELK4, strong binding to the wild-type *IRF3/BCL2L12* promoter region was confirmed in ENCODE ChIP-seq data (Figure S10). Instead, the effect of the g.49665847C>T (GenBank: NC_000019.10) mutation is more ambiguous. Besides creating weak ETS binding sites, these mutations are predicted to disrupt binding of SP and E2F family factors (Figure S9). Among these, SP1 and E2F4 showed the strongest binding to the wild-type promoter sequence (Figure S10). ENCODE DNase-seq and H3K27ac and H3K4me3 ChIP-seq data also support that the wild-type *IRF3/BCL2L12* promoter region corresponds to open active chromatin in primary melanocytes (Figure S10). Furthermore, all three identified mutations significantly reduced promoter activity in a *BCL2L12* luciferase reporter assay (Figure 3E). Unfortunately, no mutational effects could be shown in *IRF3* promoter luciferase reporter assays (data not shown), which may be due to use of non-optimal *IRF3* promoter sequences in our reporters. Additionally, we performed reporter assays incorporating additional mutations in the *BCL2L12* promoter region that abolish all predicted transcription factor binding sites in the region of our mutations (Figure S11). Mutation of g.49665843-48 (GenBank: NC_00019.10) reduced *BCL2L12* promoter activity by 25% (Figure 3F), as we had observed for the g.49665847C>T (GenBank: NC_00019.10) *BCL2L12* promoter reporter assay (Figure 3E). This further supports that the g.49665847C>T (GenBank: NC_00019.10) mutation is disrupting SP/E2F binding rather than creating an ETS binding site. Consistent with our findings for the g.49665874C>T (GenBank: NC_00019.10) and g.49665875C>T (GenBank: NC_00019.10) reporter assays (Figure 3E), mutation of the g.49665874-78 (GenBank: NC_00019.10) region resulted in a 29% decrease in promoter activity (Figure 3F). These findings thus further support that the g.49665847C>T (GenBank: NC_00019.10), g.49665874C>T (GenBank: NC_00019.10), and g.49665875C>T (GenBank: NC_00019.10) mutations disrupt transcription factor binding in the *IRF3/BCL2L12* promoter.

Given that both IRF3 and BCL2L12 are regulators of TP53,^32^^,^^33^^,^^34^^,^^35^^,^^36^ we evaluated the expression of the TP53 tumor suppressor and its primary target cyclin-dependent kinase inhibitor 1A (*CDKN1A* [MIM: 116899]) (encoding P21). In melanoma tumors with *IRF3/BCL2L12* promoter mutations, *TP53* was significantly downregulated on the mRNA level and *CDKN1A* showed a trend toward downregulation (Figure 3G). This downregulation of TP53 and CDKN1A was confirmed in our Mel-ST cell model at mRNA and protein level (Figures 3C, 3H, and 3I). Furthermore, the IRF3 transcription factor is a potent activator of type I interferon (IFN) genes and IFN-stimulated genes (ISGs) in response to DNA damage.^37^ However, we were not able to show consistent impairment of IFN or ISG activation upon DNA damage in our Mel-ST models. IFN signaling is also essential for the targeting of melanoma cells by the immune system and for the proper functioning of immunotherapies that are part of the standard of care for melanoma.^38^^,^^39^^,^^40^ As the IFN-regulatory role of IRF3 plays a pivotal part in this, we tested whether the non-coding mutations we had identified could affect immunotherapy response in individuals with melanoma. Although the number of persons with mutated tumors that could be analyzed was limiting the statistical power, the fraction of non-responders or individuals with stable disease upon administration of immune-checkpoint therapy was 19% higher in individuals with melanoma tumors with non-coding mutations in the *BCL2L12/IRF3* promoter as compared to individuals without such mutations (Figure 3J). In conclusion, we demonstrate that *IRF3/BCL2L12* promoter mutations are functional through their impact on IRF3 and BCL2L12 expression and TP53 regulation, suggesting a potential link to immunotherapy response.

### Mutation clusters in oncogene *KNSTRN* and in *SLC27A5* correspond to non-coding gene-promoter mutations

**Figure 4:**
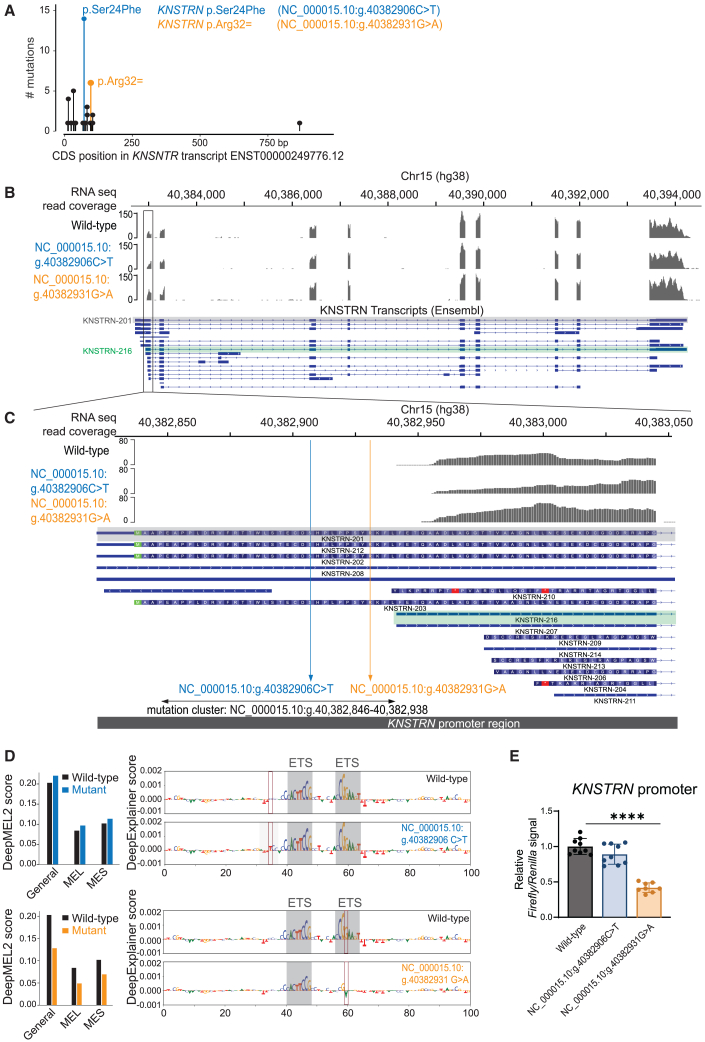
Reannotation of KNSTRN mutations in SKCM as promoter mutations (A) Needleplot of COSMIC mutations in *KNSTRN* in malignant melanoma in respect to transcript ENST00000249776.12, the default transcript shown in COSMIC. (B) RNA-seq data from melanoma tumors were mapped against hg38 in IGV. An RNA-seq read distribution plot of a representative wild-type and *KNSTRN* mutated tumor is shown in the upper part of the panel. The reported *KNSTNR* transcripts in Ensembl are indicated, the *KNSTRN-201* reference transcript is highlighted in gray, and the *KNSTRN-216* transcript that is expressed in melanoma is highlighted in green. The gray rectangle indicates the region where the *KNSTRN* promoter variants are located and is shown in more detail in (C). (C) More detailed image of the area in the gray box in (B). This image clearly shows the location of the non-coding *KNSTRN* promoter mutations in respect to expressed transcript *KNSTRN-216*. (D) Scoring of the *KNSTRN* promoter variants (g.40382906C>T [GenBank: NC_000015.10] and g.40382931G>A [GenBank: NC_000015.10]) using DeepMEL2, a melanoma-specific deep-learning model to interpret how sequence variation affects gain or loss of TF binding sites.^24^ DeepMEL2 predictions are based on training the model based on three classes/topics, which represent a general melanoma (general), a melanocytic (MEL), and a mesenchymal (MES) state. The visualization on the right shows the nucleotide targeted by the mutation (indicated by a red rectangle) as well as the nucleotides upstream and downstream and illustrates the disruption (g.40382931G>A [GenBank: NC_000015.10]) of an ETS transcription factor binding site that underlies the observed prediction difference. Gray shaded areas indicate an ETS binding site. (E) Ratio of firefly luciferase signal (under *KNSTRN* promoter) over *Renilla* luciferase signal (under constitutive TK promoter) as a measurement of wild-type and mutant *KNSTRN* promoter activity in reporter assays performed in HEK293T cells. Combined data of three independent experiments. Error bars indicate standard deviations. Statistics calculated by ordinary one-way ANOVA: ^∗∗∗∗^*p* < 0.0001.

The discovery of the non-coding nature of the *BCL2L12* mutations made us suspect that other identified clusters of synonymous and/or missense mutations also could be wrongly annotated in cancer genomics databases. For *KNSTRN*, a prominent clustering of synonymous and non-synonymous mutations was detected in SKCM (Figures 1B and 1C). This mutation cluster targeting nucleotides 4–103 of the *KNSTRN* reference transcript was confirmed when analyzing melanoma samples in COSMIC (Figure 4A), and the mutations were detected in 4.4%–5.5% of melanoma tumors (46/1,034 in COSMIC and 26/470 in TCGA-SKCM). Ensembl reports 16 *KNSTRN* transcripts. To verify which of these transcripts are expressed in SKCM, we mapped RNA-seq data from *KNSTRN* wild-type and mutated melanoma to the genome. In all analyzed melanomas, the identified mutations were located upstream of the expressed *KNSTRN* transcripts. Indeed, the analysis showed that SKCM tumors do not express the reference transcript *KNSTRN-201*. Instead, they express *KNSTRN-216*, in respect to which the identified mutations are non-coding *KNSTRN* promoter variants (Figures 4B and 4C). *KNSTRN-216* was also confirmed as main expressed transcript in Mel-ST cells using nanopore sequencing (Figure S12). *KNSTRN* expression levels were not significantly different between melanoma tumors with a wild-type or mutant *KNSTRN* promoter status (Figure S13), which may be due to the limited number of mutated tumors for which expression data were available. To further evaluate the role of the identified mutations in regulating *KNSTRN* mRNA expression, we again applied DeepMEL2 and analyzed the two most frequent mutations in the cluster.^24^ Whereas the effect of g.40382906C>T (GenBank: NC_000015.10) was predicted to be minor, g.40382931G>A (GenBank: NC_000015.10) resulted in disruption of ETS transcription factor binding (Figure 4D). Additionally, we applied the PhysBinder and FABIAN-variant tools. In line with the DeepMEL2 results, these tools predict very minor changes to transcription factor binding sites for g.40382906C>T (GenBank: NC_000015.10). They also predict the disruption of transcription factor binding sites of ETS family factors for the g.40382931G>A (GenBank: NC_000015.10) mutation, in particular of ETS1, ELK1, ELF1, and GABPA (Figure S14A). Publicly available ChIP-seq data in cell lines from which data were available confirm binding peaks for ELK1, ELF1, ETS1, and GABPA at the site of the locations. Additionally, DNase-seq data, along with H3K27ac and H3K4me3 ChIP-seq data, confirmed that the KNSTRN mutation region is active in primary melanocytes (Figure S14B). Taken together, these results suggest that g.40382931G>A (GenBank: NC_000015.10) is a functional mutation that disrupts an ETS binding site, whereas g.40382906C>T (GenBank: NC_000015.10) does not. In line with these predictions, g.40382931G>A (GenBank: NC_000015.10) was associated with a 58% reduction of promoter activity in a luciferase reporter assay (Figure 4E), further underscoring the functionality of this particular *KNSTRN* promoter mutation.

**Figure 5:**
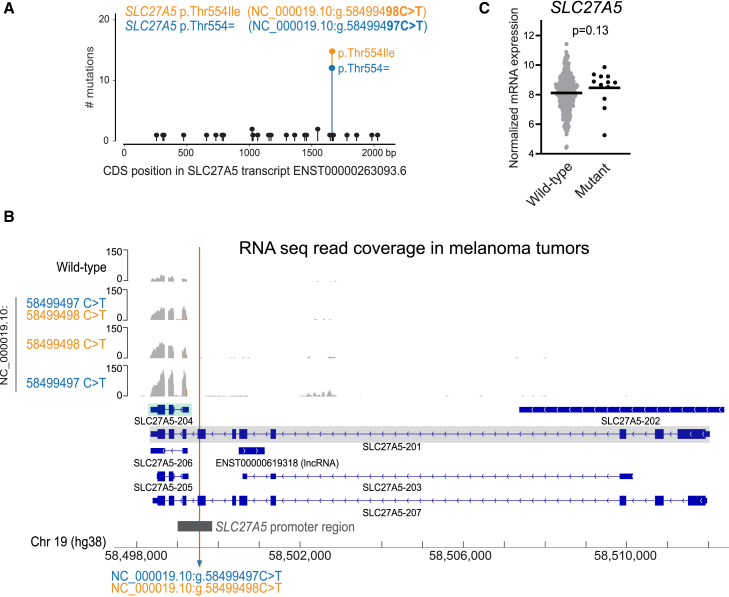
Reannotation of *SLC27A5* mutations in SKCM as promoter mutations (A) Needleplot of COSMIC mutations in *SLC27A5* in malignant melanoma in respect to transcript ENST00000263093.6, the default transcript shown in COSMIC. (B) RNA-seq data from melanoma tumors mapped against hg38. This analysis reveals that transcript *SLC27A5-204* is expressed in melanoma (indicated with green shading) instead of the *SLC27A5-201* reference transcript (gray shading). In respect to expressed transcript *SLC27A5-204*, the identified *SLC27A5* mutations in melanoma are non-coding. (C) Normalized *SLC27A5* mRNA expression in melanoma tumors with a wild-type (*n* = 422) or mutated (g.58499497C>T [GenBank: NC_000019.10] or g.58499498C>T [GenBank: NC_000019.10]; *n* = 12) *SLC27A5* promoter region. Horizontal lines indicate median expression. Statistics calculated by Mann-Whitney U test.

We subsequently also verified *SLC27A5* and *CAMK4*, in which we identified mutation clusters of interest in melanoma (Figure 1C). For both genes, the identified mutational hotspots were confirmed in COSMIC (Figures 5A and S15A). Analysis of expressed transcripts in melanoma revealed that the *SLC27A5* mutations also corresponded to non-coding promoter mutations in respect to expressed transcript *SLC27A5-204* (Figures 5B and S16), whereas the CAMK4 c.255C>T (GenBank: NM_001744.6) (p.Ile85=) mutations are true synonymous mutations in respect to the expressed transcripts (Figures S15B and S15C). The *SLC27A5* promoter mutations were associated with increased *SLC27A5* expression in melanoma tumors (Figure 5C). However, DeepMEL2 and luciferase reporter assays did not indicate any effect on promoter activity (Figure S17).

In conclusion, we show that three out of four of the identified cluster of mutations, which consist of mutations that were formerly annotated as synonymous and missense mutations in commonly used cancer genomics databases such as TCGA and COSMIC, correspond to functional non-coding promoter mutations that can affect gene-expression levels. This discovery was made because we verified the transcripts that are expressed in SKCM. This is typically not done in cancer genomics databases, where mutations are annotated in respect to a reference transcript for which expression in the analyzed tumor type is not analyzed.

### Annotation in respect to expressed transcripts reveals that 22% of identified mutation clusters in SKCM are non-coding

We then wanted to verify how widespread is the problem of wrong annotation of cancer mutations in SKCM. Therefore, we utilized RNA-seq data from 35 TCGA melanoma tumors and applied Salmon^13^ to identify the main expressed transcript in SKCM for the 52 genes in which the most significant mutation clusters were found (Figure 1B). Next, we annotated the most frequent mutation in each of these clusters in respect to the main expressed transcript in SKCM using Ensembl VEP^14^ (Table 1). This Salmon/VEP method correctly identified the mutations in *SLC27A5*, *BCL2L12*, and *KNSTRN* as upstream gene variants and the *CAMK4* mutation as synonymous. To evaluate the accuracy of this Salmon/VEP method in more detail, we first verified whether Salmon was able to identify the correct main expressed transcript in SKCM by inspection of mapped RNA-seq data of 35 melanoma tumors against the genome in the IGV (Table S8). For two out of the 52 tested genes, expression levels were too low to identify the expressed transcript in IGV. For 44 out of the remaining 50 genes, the Salmon-identified main transcript was confirmed in IGV, giving Salmon an accuracy of 88% at identifying the correct main expressed transcript. For three genes, namely OXA1L mitochondrial inner membrane insertase (*OXA1L* [MIM: 601066]), G-protein subunit alpha 11 (*GNA11* [MIM: 139313]), and DEAD-box helicase 28 (*DDX28* [MIM: 607618]), IGV confirmed expression of the transcript identified by Salmon, but the expressed transcript showed a different UTR or 5′ end compared to the reported transcript in Ensembl (Figures S18–S20). The RNA-seq data from melanoma that we utilized to determine the main expressed transcript are based on Illumina sequencing technology. To rule out that Illumina sequencing artifacts would result in identification of incorrect main expressed transcripts, we also performed direct cDNA sequencing on Mel-ST cells using Oxford Nanopore technology (ONT) (Table S8; Figures S5, S12, and S16). Mel-ST cells expressed 43 of the 52 genes of interest, and for 42 of the 43 expressed genes, the ONT sequencing data confirmed the main expressed transcript that was identified in the Illumina-based SKCM RNA-seq data, ruling out major artifacts from the used sequencing technology. Next, we focused on the accuracy of the mutation annotation. The 50 mutations that could be evaluated were all manually annotated in IGV, and the result that was obtained in this way was compared to the annotation obtained by Salmon/VEP and to the VEP annotation of the IGV- and ONT-validated main expressed transcript in SKCM (Table 1). For 45 out of 50 mutations, Salmon/VEP resulted in the same annotation as the one that was manually obtained in IGV, resulting in 90% accuracy of Salmon/VEP. The annotation accuracy was further increased to 96% (48/50) when performing VEP annotation on the IGV- and ONT-corrected main expressed isoform in SKCM (Table 1). The two mutations that still could not be correctly annotated by this last method were in prostaglandin E receptor 3 (*PTGER3* [MIM: 176806]), because the identified main expressed transcript was not annotated by VEP, and in *OXA1L*, because the *OXA1L* transcript that is expressed in SKCM samples is not reported in Ensembl. The transcript that was identified by Salmon as expressed is similar but has a different 5′ starting position, resulting in wrong mutation annotation by Salmon/VEP (Figure S18).

**Table 1:** Reannotation of SKCM mutations based on main expressed transcript

**Gene**	**Mutation (hg38)**	**Former annotation**	**Main expressed SKCM transcript (Salmon) and VEP annotation**	**IGV- and nanopore-sequencing-validated main SKCM transcript and VEP annotation**	**IGV-validated annotation (SKCM)**
*BRAF*	g.140753336T>A (GenBank: NC_000007.14)	missense	ENST00000496384_missense	ENST00000496384_missense	missense
*NRAS*	g.114713908A>G (GenBank: NC_000001.11)	missense	ENST00000369535_missense	ENST00000369535_missense	missense
*CAMK4*	g.111374864C>T (GenBank: NC_000005.10)	synonymous	ENST00000282356_synonymous	ENST00000282356_synonymous	synonymous
*SLC27A5*	g.58499498C>T (GenBank: NC_000019.10)	missense	ENST00000594786_upstream_gene_variant	ENST00000594786_upstream_gene_variant	gene promoter
*BCL2L12*	g.49665874C>T (GenBank: NC_000019.10)	synonymous	ENST00000246784_upstream_gene_variant	ENST00000246784_upstream_gene_variant	gene promoter (also IRF3)
*KNSTRN*	g.40382931G>A (GenBank: NC_000015.10)	synonymous	ENST00000608100_upstream_gene_variant	ENST00000608100_upstream_gene_variant	gene promoter
*IDH1*	g.208248389C>T (GenBank: NC_000002.12)	missense	ENST00000345146_missense	ENST00000345146_missense	missense
*BAAT*	g.101362725C>T (GenBank: NC_000009.12)	synonymous	ENST00000259407_synonymous	ENST00000259407_synonymous	synonymous
*ANKRD30B*	g.14763876G>A (GenBank: NC_000018.10)	synonymous	ENST00000320584_NA	expressed too low to evaluate	expressed too low to evaluate
*SLC9A9*	g.143574137C>T (GenBank: NC_000003.12)	synonymous	ENST00000316549_synonymous	ENST00000316549_synonymous	synonymous
*OASL*	g.121031505C>T (GenBank: NC_000012.12)	synonymous	ENST00000257570_synonymous	ENST00000679655_synonymous	synonymous
*TACC3*	g.1728651C>T (GenBank: NC_000004.12)	missense	ENST00000313288_missense	ENST00000313288_missense	missense
*RAC1*	g.6387261C>T (GenBank: NC_000007.14)	missense	ENST00000348035_missense	ENST00000348035_missense	missense
*PTGER3*	g.70852856G>A (GenBank: NC_000001.11)	missense	ENST00000370924_NA	ENST00000370924_NA	non_coding
*IL17RD*	g.57109588C>T (GenBank: NC_000003.12)	missense	ENST00000296318_missense	ENST00000296318_missense	missense
*CCDC28A*	g.138773767C>T (GenBank: NC_000006.12)	synonymous	ENST00000617445_upstream_gene_variant	ENST00000617445_upstream_gene_variant	gene promoter
*ZNF28*	g.52800669C>T (GenBank: NC_000019.10)	synonymous	ENST00000457749_synonymous	ENST00000457749_synonymous	synonymous
*NPAS1*	g.47032687C>T (GenBank: NC_000019.10)	synonymous	ENST00000439365_upstream_gene_variant	ENST00000439365_upstream_gene_variant	in enhancer
*CNOT9*;*RQCD1*	g.218584683C>T (GenBank: NC_000002.12)	missense	ENST00000273064_missense	ENST00000273064_missense	missense
*POLA2*	g.65294599C>T (GenBank: NC_000011.10)	synonymous	ENST00000265465_synonymous	ENST00000706538_synonymous	synonymous
*GNAI2*	g.50256262C>T (GenBank: NC_000003.12)	missense	ENST00000313601_missense	ENST00000313601_missense	missense
*FAM13A*	g.88737535G>A (GenBank: NC_000004.12)	synonymous	ENST00000264344_synonymous	ENST00000264344_synonymous	synonymous
*DCLK2*	g.150193204C>T (GenBank: NC_000004.12)	missense	ENST00000411937_missense, NMD_transcript_variant	ENST00000506325_missense	missense
*SMURF2*	g.64561537C>T (GenBank: NC_000017.11)	missense	ENST00000582081_3′_UTR_variant, NMD_transcript_variant	ENST00000262435_missense	missense
*RIPPLY3*	g.37018067G>A (GenBank: NC_000021.9)	missense	ENST00000329553_missense	ENST00000329553_missense	missense
*PLK1*	g.23684030C>T (GenBank: NC_000016.10)	missense	ENST00000300093_missense	ENST00000300093_missense	missense
*OXA1L*	g.22766690C>T (GenBank: NC_000014.9)	missense	ENST00000285848_missense	ENST00000285848.9 (but shorter 5′ end)_missense	gene promoter (also of OXA1L-DT)
*STXBP4*	g.55159824C>T (GenBank: NC_000017.11)	synonymous	ENST00000376352_synonymous	ENST00000376352_synonymous	synonymous
*GNA11*	g.3118944C>T (GenBank: NC_000019.10)	missense	ENST00000078429_missense	ENST00000078429 (but shorter 5′UTR)_missense	missense
*DDX41*	g.177512792C>T (GenBank: NC_000005.10)	missense	ENST00000330503_missense	ENST00000330503_missense	missense
*ATP2B1*	g.89604224C>T (GenBank: NC_000012.12)	synonymous	ENST00000635033_upstream_gene_variant	ENST00000428670_synonymous	synonymous
*TREM1*	g.41280966C>T (GenBank: NC_000006.12)	synonymous	ENST00000589695_non_coding_transcript_exon_variant	ENST00000244709_synonymous	synonymous
*TBCK*	g.106116364C>T (GenBank: NC_000004.12)	synonymous	ENST00000361687_synonymous	ENST00000361687_synonymous	synonymous
*GPCPD1*	g.5565054C>T (GenBank: NC_000020.11)	missense	ENST00000379019_missense	ENST00000379019_missense	missense
*CDK4*	g.57751653A>T (GenBank: NC_000012.12)	missense	ENST00000257904_missense	ENST00000257904_missense	missense
*AKR1B1*	g.134449780C>T (GenBank: NC_000007.14)	synonymous	ENST00000285930_synonymous	ENST00000285930_synonymous	synonymous
*ZNF329*	g.58128983C>T (GenBank: NC_000019.10)	missense	ENST00000358067_missense	ENST00000358067_missense	missense
*SWI5*	g.128276245C>T (GenBank: NC_000009.12)	missense	ENST00000418976_upstream_gene_variant	ENST00000418976_upstream_gene_variant	gene promoter (also of GOLGA2)
*RHOA*	g.49360277G>A (GenBank: NC_000003.12)	missense	ENST00000418115_missense	ENST00000418115_missense	missense
*DHX15*	g.24541994C>T (GenBank: NC_000004.12)	missense	ENST00000336812_missense	ENST00000336812_missense	missense
*DCAF13*	g.103415436G>A (GenBank: NC_000008.11)	synonymous	ENST00000612750_5′_UTR_variant	ENST00000612750_5′_UTR_variant	5′ UTR/gene promoter (also of SLC25A32)
*ARL16*	g.81683810G>A (GenBank: NC_000017.11)	missense	ENST00000622299_upstream_gene_variant	ENST00000622299_upstream_gene_variant	gene promoter (also of HGS)
*AIFM1*	g.130136694C>T (GenBank: NC_000023.11)	synonymous	ENST00000287295_synonymous	ENST00000287295_synonymous	synonymous
*ZBTB12*	g.31900592C>T (GenBank: NC_000006.12)	synonymous	ENST00000375527_synonymous	ENST00000375527_synonymous	synonymous
*YIPF1*	g.53878697C>T (GenBank: NC_000001.11)	missense	ENST00000464950_missense, NMD_transcript_variant	ENST00000464950_missense, NMD_transcript_variant	missense
*TDRD7*	g.97432099C>T (GenBank: NC_000009.12)	missense	ENST00000355295_missense	ENST00000355295_missense	missense
*MGAM2*	g.142196742G>A (GenBank: NC_000007.14)	missense	ENST00000477922_missense	expressed too low to evaluate	expressed too low to evaluate
*PPP1R1A*	g.54580956G>A (GenBank: NC_000012.12)	synonymous	ENST00000257905_synonymous	ENST00000257905_synonymous	synonymous
*ARIH2OS*	g.48918840G>A (GenBank: NC_000003.12)	missense	ENST00000647812_upstream_gene_variant	ENST00000647812_upstream_gene_variant	gene promoter (also of ARIH2)
*TMEM154*	g.152652703C>T (GenBank: NC_000004.12)	missense	ENST00000304385_missense	ENST00000304385_missense	missense
*SLC39A6*	g.36109607C>T (GenBank: NC_000018.10)	missense	ENST00000269187_missense	ENST00000269187_missense	missense
*DDX28*	g.68021877C>T (GenBank: NC_000016.10)	synonymous	ENST00000332395_synonymous	ENST00000332395 (but shorter 3′ UTR)_synonymous	synonymous

This table reports the most frequent mutation in each of the 52 mutation clusters identified in SKCM (Figure 1A). In the column “former annotation,” we report the current annotation of these mutations as reported in cancer genomics databases. Furthermore, we report the main expressed transcript of the respective genes and their corresponding annotation (based on Salmon analysis of Illumina data from SKCM tumors and based on subsequent validation of the main expressed transcript in IGV and by long-read nanopore sequencing). Finally, in the outer right column, we report the IGV-validated mutation annotation based on the consensus main expressed transcript of the mutated gene.

When considering the manual IGV reannotation of mutations based on expressed transcripts in SKCM (Table 1, right column), we found that 11 out of 50 (22%) analyzed mutations corresponded to non-coding mutations (Table 1, gray shaded lines). IGV showed that nine of these mutations, in *SLC27A5*, *BCL2L12*, *KNSTRN*, coiled-coil domain containing 28A (*CCDC28A* [MIM: 615353]), *OXA1L*, SWI5 homologous recombination repair protein (*SWI5* [MIM: 616528]), DDB1- and CUL4-associated factor 13 (*DCAF13* [MIM: 616196]), ARF-like GTPase 16 (*ARL16* [MIM: 619117]), and ARIH2 opposite strand lncRNA (*ARIH2OS*), were targeting the corresponding gene-promoter regions. Interestingly, in addition to the promoter mutation in *BCL2L12* that also targets the promoter of *IRF3* (Figure 2), five other of the newly identified promoter mutations (in *OXA1L*, *SWI5*, *DCAF13*, *ARL16*, and *ARIH2OS*) were also targeting the promoter of an adjacent gene OXA1L divergent transcript (*OXA1L-DT*), golgin A2 (*GOLGA2* [MIM: 602580]), solute carrier family 25 member 32 (*SLC25A32* [MIM: 138480]), hepatocyte growth factor-regulated tyrosine kinase substrate (*HGS* [MIM: 604375]), and ariadne RBR E3 ubiquitin protein ligase 2 (*ARIH2* [MIM: 605615]), respectively (Figures S18 and S21–S24).

**Figure 6:**
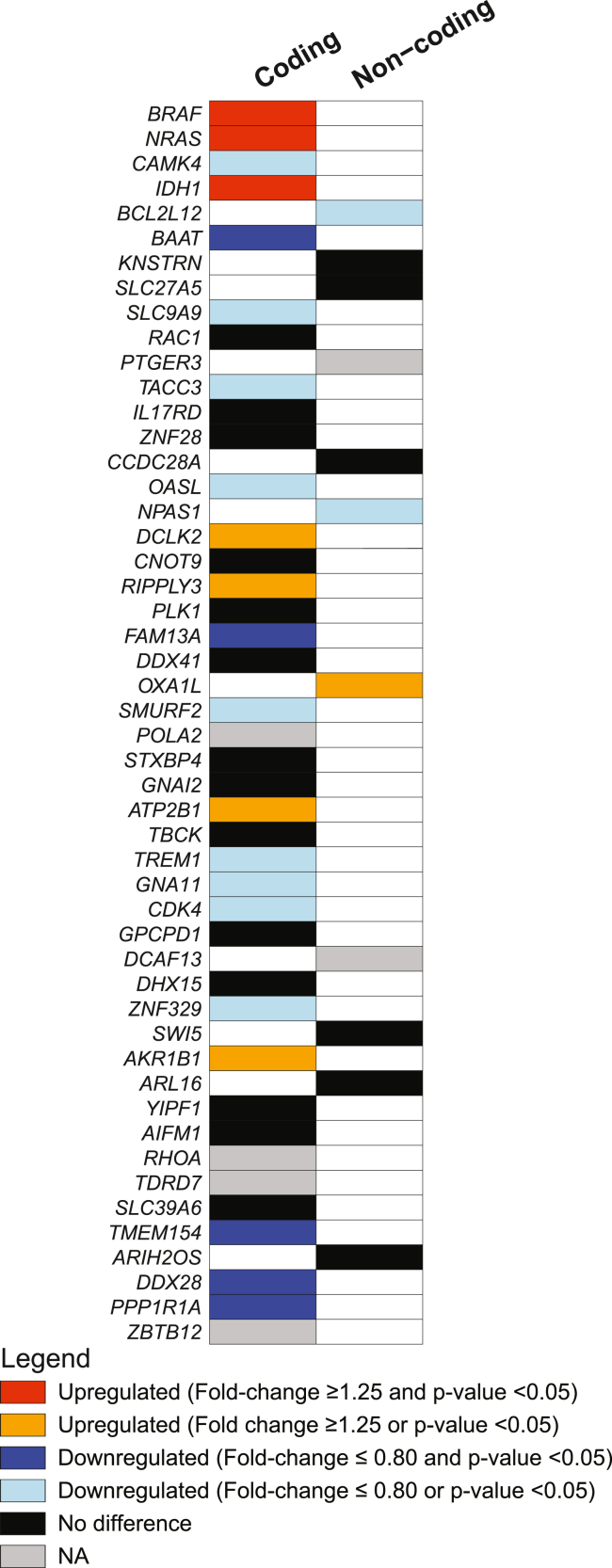
Impact of SKCM mutation clusters on RNA expression of their host gene Differential RNA expression of the indicated genes in SKCM tumors with a mutated status for the identified mutation cluster in that gene (mutation cluster defined here as the region ranging from 30 nt upstream to 30 nt downstream of the most recurrent mutation) as compared to tumors with a wild-type status for the gene of interest. The genes are divided into two columns to clearly indicate whether the mutation cluster was identified as coding (missense and synonymous mutations) or non-coding. *MGAM2* and *ANKRD30B* were not included because the coding or non-coding nature of these mutations is unclear. The legend in the figure explains the color coding. Statistical testing was done in DESeq2.

Finally, we also evaluated whether the non-coding mutations that we identified in SKCM may be functional. We therefore compared RNA-expression levels of tumors with a wild-type or mutated status for the identified mutation cluster in the gene of interest and tested whether these mutations were associated with a significant change in gene expression of the host gene (Figure 6 and Table S9). Although the number of mutated tumors was severely limiting the statistical power, especially for the mutations with lower frequency, this analysis revealed that, in addition to *BCL2L12*, the non-coding mutations in neuronal PAS domain protein 1 (*NPAS1* [MIM: 603346]) and *OXA1L* were also associated with respective down- and upregulation of their host gene.

In conclusion, combining Salmon and VEP to annotate cancer mutations in respect to the transcripts that are expressed in a particular tumor type represents a simple automated methodology to more correctly annotate cancer mutations, which allowed us to obtain an accuracy of 90% in melanoma. Furthermore, we found that 11 of the 50 (22%) analyzed SKCM mutations annotated as synonymous or missense in cancer genomics databases in reality correspond to non-coding mutations.

## Discussion

Previous studies that identified pathogenic synonymous mutations in cancer are limited by prioritizing well-known cancer genes, ignoring co-clustering of synonymous and missense mutations, or relying solely on DNA-seq. In this work, we developed a method that overcomes these limitations when screening for candidate pathogenic mutations in cancer and that can be applied to both synonymous and non-synonymous mutations. Our approach identified numerous mutation hotspots in known oncogenes and genes not yet implicated in (skin) cancer. On the other hand, the re-identification of well-established mutations in oncogenes such as *BRAF* and *NRAS* validated the robustness of our approach. However, less-frequent variants or variants in tumor types with limited samples may have been missed due to the stringent filtering that we applied throughout the pipeline. It remains challenging to computationally identify strong candidate drivers that occur less frequently.

Most other available mutation detection pipelines and cancer genomics databases such as COSMIC and Genomic Data Commons by default report on mutations annotated relative to a reference transcript, which can result in wrong mutation annotation as we show in this work. Indeed, mutation annotation can change depending on which gene transcript is expressed. In COSMIC, researchers have the option to select other transcripts against which mutations should be annotated, allowing for greater flexibility in aligning mutations with the expressed transcripts. However, this functionality is not available in all databases. As a result, mutations in genes such as *BCL2L12*, *KNSTRN*, and *SLC27A5* are currently consistently annotated incorrectly.

In particular, we have recharacterized g.49665874C>T (GenBank: NC_000019.10), which was described as a synonymous BCL2L12 p.Phe17= mutation by Gartner et al. in 2013.^10^ At the time, Gartner et al. considered BCL2L12 transcript GenBank: NM_138639.1, in which g.49665874C>T (GenBank: NC_000019.10) indeed corresponds to a synonymous p.Phe17= mutation. However, *BCL2L12* has multiple transcripts, and their annotation has changed over time. Transcript GenBank: NM_138639.1 was updated to GenBank: NM_138639.2 in 2020, which no longer overlaps with g.49665874C>T (GenBank: NC_000019.10). Nevertheless, cancer genomics databases such as COSMIC and Genomics Data Commons are still annotating g.49665874C>T (GenBank: NC_000019.10) as synonymous, as they are using a *BCL2L12* reference transcript that is not expressed in melanoma for annotation. Interestingly, we identified two additional non-coding mutations targeting the shared *IRF3/BCL2L12* promoter region at g.49665847 (GenBank: NC_000019.10) and g.49665875 (GenBank: NC_000019.10). Together with the first hotspot, these mutations have a combined frequency of 3.8%–5.5% and result in downregulation of *BCL2L12* and *IRF3* expression in melanoma tumors.

We also generated highly accurate isogenic knockin models using CRISPR-Cas9 to study the effect of these non-coding mutations when introduced at the endogenous locus. Importantly, no additional mutations were introduced to avoid recutting by Cas9. This unique model is a significant advancement in the field of synonymous and non-coding mutations where typically only *in silico* predictions, reporter assays, or overexpression methodologies are used. Heterozygous knockin of any of the three mutations in Mel-ST cells significantly downregulated BCL2L12 and IRF3 mRNA and protein levels. This co-regulation aligns with findings by Nakajima et al. when deleting a 1.6-kb region in the shared promoter of *BCL2L12* and *IRF3* in mice.^41^ Notably, our study demonstrates a significant effect with only single-nucleotide conversions, highlighting the functionality of these subtle genetic alterations. The downstream effect of these non-coding mutations is further underscored by downregulation of TP53—a major tumor suppressor known to be regulated by BCL2L12 and IRF3—and its primary target CDKN1A. Our findings contrast with those of Gartner et al., who previously described these mutations as synonymous.^10^ We show that they are non-coding mutations affecting ETS transcription factor binding sites and gene-promoter activity, leading to decreased expression rather than the miRNA-mediated increased expression proposed by Gartner et al.^10^ Although we were unable to validate the impact of the mutations on IFN signaling in our Mel-ST cell model, our data suggest a negative effect on immunotherapy response in individuals with melanoma. Experiments in cultured Mel-ST cells may not recapitulate these effects, which likely require *in vivo* interactions with various immune cell populations. Given the importance of immunotherapy in melanoma, the modulation of IRF3 function by these mutations and its downstream immune effects merits further exploration. Nonetheless, we characterized three single-nucleotide mutations in a non-coding region that demonstrate a clear functional impact, contributing to dysregulation of a major tumor suppressor such as TP53. Other studies demonstrating such significant effects from non-coding point mutations are extremely rare. The only other well-established example are the mutations in the promoter of the telomerase reverse transcriptase (*TERT* [MIM: 187270]). These mutations induce expression of the TERT oncogene by creating a GABPA transcription factor binding site in glioblastoma and melanoma.^42^^,^^43^^,^^44^^,^^45^ To the best of our knowledge, these non-coding mutations have not been studied at the endogenous level in an isogenic cell model as we did.

In *KNSTRN*, we identified an intriguing cluster of non-coding promoter mutations present in 4.4%–5.5% of melanoma cases. Lee et al. described the KNSTRN p.Ser24Phe mutation in cutaneous squamous cell carcinoma, a less-aggressive type of skin cancer arising from keratinocytes.^46^ The KNSTRN p.Ser24Phe mutation described by Lee et al. corresponds to g.40382906C>T (GenBank: NC_000015.10), the most frequent mutation in the *KNSTRN* promoter mutation cluster that we identified. Interestingly, elevated *KNSTRN* expression affects the immune microenvironment and is a poor prognostic marker for immunotherapy response across different cancer types,^47^^,^^48^ inviting further investigation of the impact of *KNSTRN* promoter mutations on *KNSTRN* expression in larger tumor cohorts. Our results for the two most frequent *KNSTRN* mutations show that g.40382931G>A (GenBank: NC_000015.10) disrupts an ETS transcription factor binding site and that *KNSTRN* promoter activity is significantly reduced, whereas this is not the case for g.40382906C>T (GenBank: NC_000015.10). However, this latter mutation may still be functional, potentially regulating genes located further away,^49^ since the remarkable clustering of mutations strongly suggests positive selection and functionality. A similar intriguing cluster of non-coding mutations was observed in *SLC27A5*, a member of the *SLC27* family that encodes a long-chain fatty acid transport protein involved in lipid metabolism. While little is known about the role of SLC27A5 in cancer, these mutations may be relevant in the light of the established importance of lipid metabolism dysregulation in cancer.^50^

Finally, we propose an automated approach integrating the freely available Salmon^13^ and Ensembl VEP^14^ tools to identify the expressed transcript for a specific tumor type and annotate the mutations accordingly. Using this method, we could show an accuracy of 90% to annotate mutations in SKCM, and we found that 22% (11/50) of analyzed mutations in melanoma were misclassified as synonymous or missense, whereas they correspond to non-coding mutations as experimentally validated by long-read sequencing. Although we annotated mutations in respect to the main expressed transcript, we observed that additional transcripts were also expressed at lower abundance for some genes. In certain cases, this may result in mutations that function both as coding and non-coding mutations, a phenomenon that warrants further investigation.

We also applied Salmon/VEP on the mutation clusters identified in two other tumor types (breast cancer [BRCA] and low-grade glioma/glioblastoma-multiform [LGGGBM]). In these other tumor types, no clear examples of non-coding mutations could be identified. This could either be due to the more limited number of highly significant mutation clusters that were identified in these other tumor types (*n* = 16 for BRCA and *n* = 23 for LGGGBM) but most likely also reflects the higher incidence of non-coding mutations in gene-promoter regions in SKCM. Indeed, several of the non-coding mutations picked up by our pipeline in melanoma target the ETS family binding motif TTCCGG.^51^ This motif is known to be frequently targeted by UV-induced mutations in melanoma, and this has previously resulted in identification of a large number of non-coding mutations in melanoma.^52^^,^^53^ The winged helix repair factor 1 (WHR1, previously known as STK19) c.265G>A (GenBank: NM_004197.1) (p.Asp89Asn) mutation is another example of a misannotated gene-promoter mutation in melanoma.^54^ This mutation was also detected by our concentration area method (Table S2) and is part of a cluster of non-coding mutations entailing 93 nucleotides (Figure S26). This illustrates once more that our work reveals that additional mutations, previously misannotated as synonymous or missense mutations, have been overlooked.

In conclusion, this work makes a significant contribution to the cancer genomics field by identifying functional non-coding mutations in melanoma and by experimentally testing them in highly accurate CRISPR-Cas9 models. Furthermore, we propose a methodology to identify functional synonymous and missense mutations and address misannotation of mutations through the integration of DNA-seq and RNA-seq data. Correct annotation of mutations is critical for researchers designing experiments, as experimental approaches to analyze non-coding mutations are different from those that are relevant for synonymous and missense mutations. Also, in a clinical setting accurate mutation annotation is highly desirable. While we acknowledge that the accuracy of Salmon/VEP is not 100%, we strongly encourage researchers to verify expressed transcripts and avoid relying solely on annotation in respect to a reference transcript for mutation annotation.

## Data availability

ONT sequencing results from Mel-ST cells have been made available at NCBI SRA (BioProject accession number BioProject: PRJNA1196620) and GEO (accession number GEO: GSE290597). Codes for our own developed mutational concentration analyses (Hotspot 12, Concentration area, and Entropy) and the used SKCM dataset are available on Github: https://github.com/Lab-DMC/reannotation_mutations.git.

## Acknowledgments

This project was funded by the 10.13039/501100000781European Research Council (ERC) under the European Union’s Horizon 2020 research and innovation program (grant agreement no. 862246). X.J. is recipient of an 10.13039/501100003130FWO aspirant mandate fundamental research grant (no. 1174021N). G.M.M.-L. was supported by a 10.13039/501100004040KU Leuven PDM mandate (PDMt1/23/021). Research by V.K. is funded by the Belgian 10.13039/501100013845Foundation against Cancer (F/2020/1396 and F/2024/140) and 10.13039/501100003130FWO-EOS (ID: 40007513). Research by B.D., A.V., and J.D.B. is supported by the 10.13039/100016895Belgian Foundation against Cancer (clinical mandate 2021/1605, research project C/2022/1941) and by the Funds Catharina Weekers, Raymond Wuyts, Arlette Lemaître, Yvette Gembauve, and Cambier-Sandra, managed by the 10.13039/501100006282King Baudouin Foundation. We are grateful to OHMX.bio and to genOway for their excellent services in this project and wish to thank Prof. Stein Aerts for his constructive suggestions and support on the *in silico* predictions of effects on transcription factor binding.

## Declaration of interests

The authors declare no conflicts of interest.

## Web resources

MC3 working group variant calls, https://gdc.cancer.gov/about-data/publications/mc3-2017
NCI Genomic Data Commons PanCanAtlas publications, https://gdc.cancer.gov/about-data/publications/pancanatlas
OMIM, https://www.omim.org/

## Supplemental information

Document S1. Figures S1–S26, Table S1, and Tables S7–S11

Table S2. Results of concentration area method Table reporting results obtained for the concentration area method that was applied on the pre-processed MC3 working group variant list. Results obtained when considering non-synonymous mutations (nSMs), synonymous mutations (SMs), and non-synonymous and synonymous mutations together (All) are reported in different tabs in the Excel file. Columns O–U in the sheet always report on the most frequent mutation in the identified mutation cluster. Note that mutation positions are indicated in respect to hg19, as in the MC3 working group dataset.

Table S3. Results of hotspot 12 method Table reporting results obtained for the hotspot 12 method that was applied on the pre-processed MC3 working group variant list. Results obtained when considering non-synonymous mutations (nSMs), synonymous mutations (SMs), and non-synonymous and synonymous mutations together (All) are reported in different tabs in the Excel file. Columns O–U in the sheet always report on the most frequent mutation in the identified mutation cluster. Note that mutation positions are indicated in respect to hg19, as in the MC3 working group dataset.

Table S4. Results of hotspot 3 method Table reporting results obtained for the hotspot 3 method that was applied on the pre-processed MC3 working group variant list. Results obtained when considering non-synonymous mutations (nSMs), synonymous mutations (SMs), and non-synonymous and synonymous mutations together (All) are reported in different tabs in the Excel file. Note that mutation positions are indicated in respect to hg19, as in the MC3 working group dataset.

Table S5. Results of entropy method Table reporting results obtained for the entropy method that was applied on the pre-processed MC3 working group variant list. Results obtained when considering non-synonymous mutations (nSMs), synonymous mutations (SMs), and non-synonymous and synonymous mutations together (All) are reported in different tabs in the Excel file. Columns I–O in the sheet always report on the most frequent mutation in the gene. Note that mutation positions are indicated in respect to hg19, as in the MC3 working group dataset.

Table S6. Concentration results heatmaps Table reporting mutations in the genes depicted in the heatmaps in Figures 1B and S2.

Document S2. Article plus supplemental information
