# Supplementary material for: Reannotation of cancer mutations based on expressed RNA transcripts reveals functional non-coding mutations in melanoma
Source: Am J Hum Genet. 2025 May 12;112(6):1447–67. doi: 10.1016/j.ajhg.2025.04.005 (PMC12256795; doi:10.1016/j.ajhg.2025.04.005)
Supplement: Document S1. Figures S1–S26, Table S1, and Tables S7–S11 [file mmc1.pdf]

The American Journal of Human Genetics, Volume *112*

Supplemental information

Reannotation of cancer mutations based on expressed RNA transcripts reveals functional non-coding mutations in melanoma

Daniele Pepe, Xander Janssens, Kalina Timcheva, Grecia M. Marrón-Liñares, Benno Verbelen, Vasileios Konstantakos, Dylan De Groote, Jolien De Bie, Amber Verhasselt, Barbara Dewaele, Arne Godderis, Charlotte Cools, Mireia Franco-Tolsau, Jonathan Royaert, Jelle Verbeeck, Kim R. Kampen, Karthik Subramanian, David Cabrerizo Granados, Gerben Menschaert, and Kim De Keersmaecker

MC3 variant list pre-processing

- Only keep PASS FILTER variants
- Only keep SNVs with protein coding annotation
- Removal of variants in immunoglobulin, HLA, T-cell receptor and olfactory genes
- Removal of variants in non-expressed genes in dataset
- Removal of variants in hypermutated tumor samples
- Remove all SNVs with a VAF < 0.2

Mutational concentration analyses

- Performed on Synonymous Mutations (SMs) only, Non-Synonymous coding Mutations only (nSMs), or SMs and nSMs together (All mutations))
- Performed on 17 different tumor types with >200 samples
- **Entropy:**
  genes with ≥ 5 mutations, maximal entropy value of 0.2
  and significant permutation test
- **Concentration:**
  genes with ≥ 5 mutations, clustering of at least 70% of gene mutations
  in maximum 40% of the transcript length and significant permutation test
- **Hotspot 12:**
  genes with ≥ 5 mutations, clustering of at least 40% of gene mutations
  in a window of 12 contiguous nucleotides in the transcript and significant permutation test
- **Hotspot 3**:
  Method by Chang et al., *Nature Biotechnology* **34**, 155-163 (2016)
- Removal of genes that are not expressed in the analyzed tumor type

Results reporting:

- **All results:** Tables S2-5
- **Heatmaps:** Representation of genes detected by 2 concentration methods in the analyzed tumor type. For tumor types with more than 100 genes detected by 2 concentration methods (SKCM), only genes detected by 3 concentration methods are shown. Mutations shown in the heatmap are described in Table S6

Figure S1. Analysis pipeline to identify clusters of synonymous and missense mutations. Overview of analysis pipeline to identify clusters of synonymous and missense mutations in TCGA cancer genomics data.

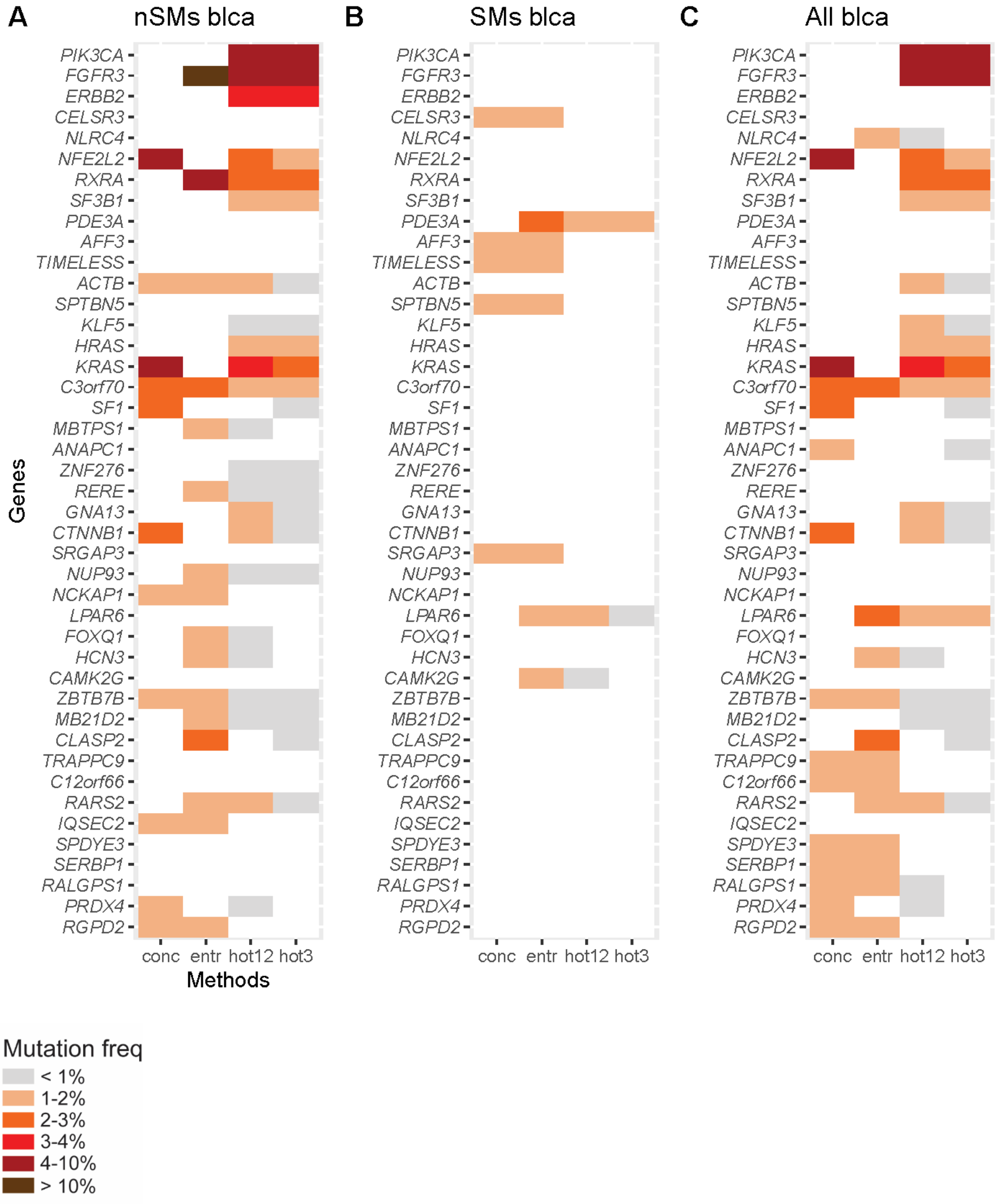
A
nSMs blca
B
SMs blca
C
All blca
Genes
PIK3CA
FGFR3
ERBB2
CELSR3
NLRC4
NFE2L2
RXRA
SF3B1
PDE3A
AFF3
TIMELESS
ACTB
SPTBN5
KLF5
HRAS
KRAS
C3orf70
SF1
MBTPS1
ANAPC1
ZNF276
RERE
GNA13
CTNNB1
SRGAP3
NUP93
NCKAP1
LPAR6
FOXQ1
HCN3
CAMK2G
ZBTB7B
MB21D2
CLASP2
TRAPPC9
C12orf66
RARS2
IQSEC2
SPDYE3
SERBP1
RALGPS1
PRDX4
RGPD2
conc
entr
hot12
hot3
Methods
Mutation freq
< 1%
1-2%
2-3%
3-4%
4-10%
> 10%

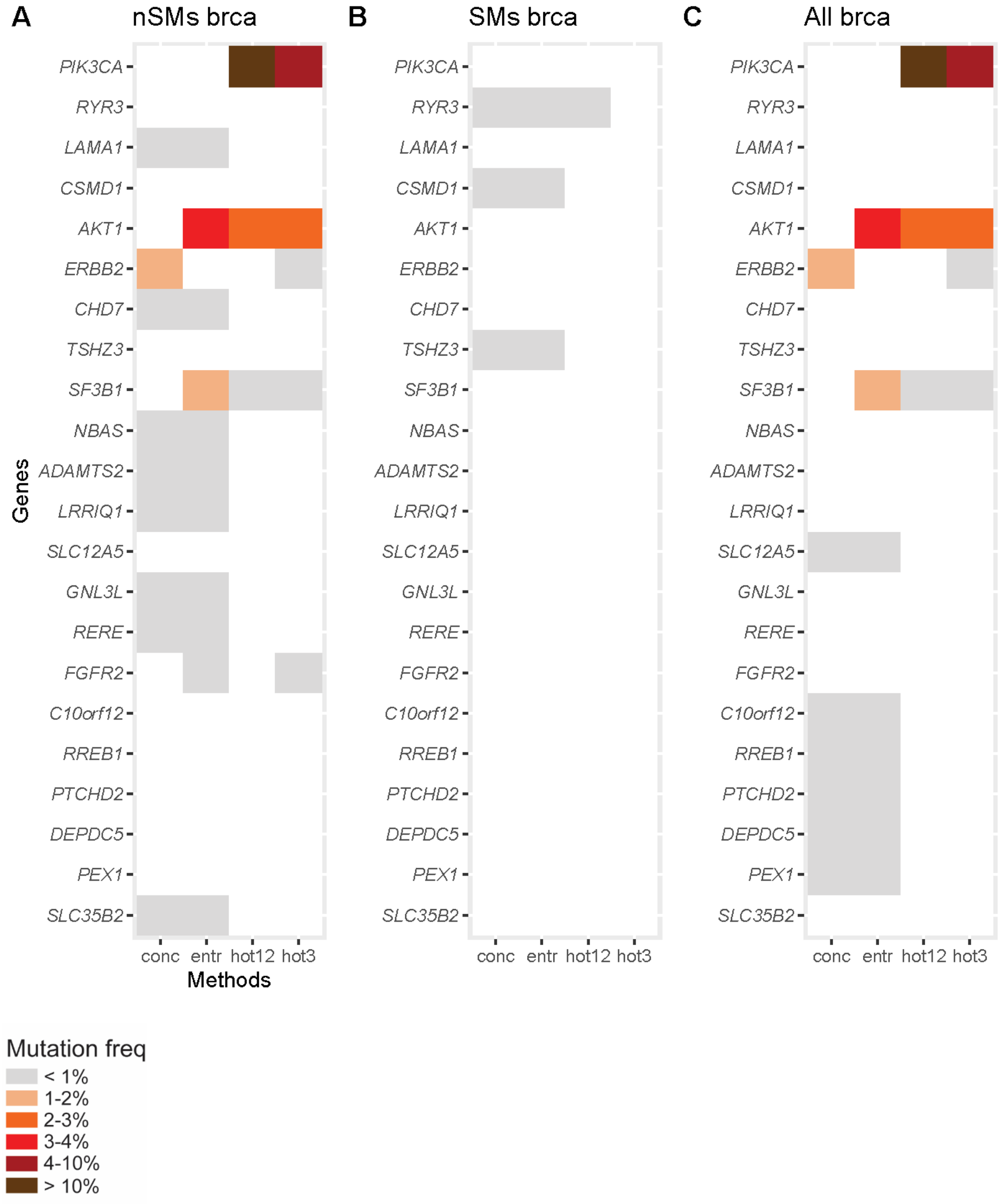
A
nSMs brca
B
SMs brca
C
All brca
PIK3CA
RYR3
LAMA1
CSMD1
AKT1
ERBB2
CHD7
TSHZ3
SF3B1
NBAS
ADAMTS2
LRRIQ1
SLC12A5
GNL3L
RERE
FGFR2
C10orf12
RREB1
PTCHD2
DEPDC5
PEX1
SLC35B2
Genes
conc
entr
hot12
hot3
Methods
Mutation freq
< 1%
1-2%
2-3%
3-4%
4-10%
> 10%

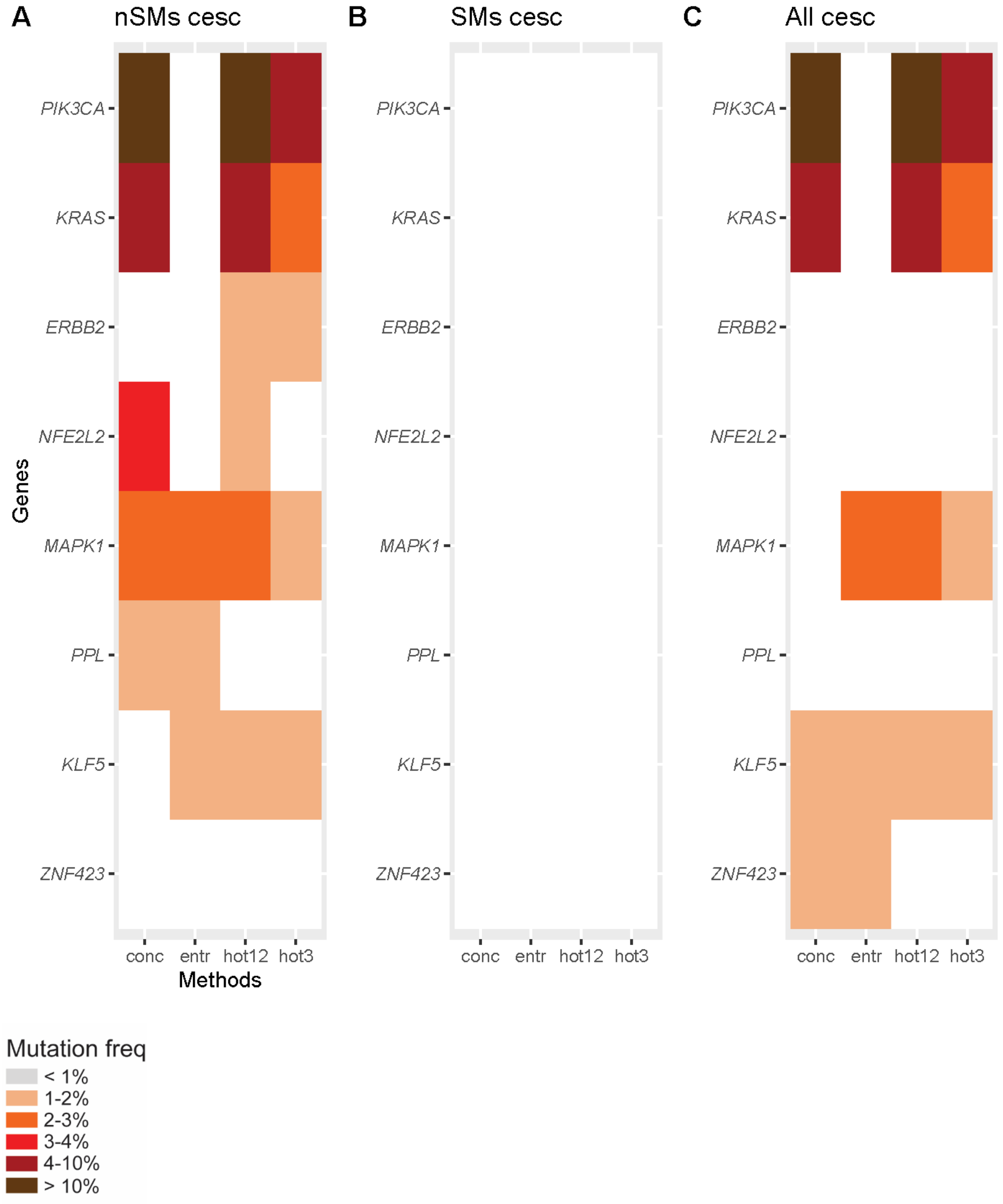
A
nSMs cesc
B
SMs cesc
C
All cesc
PIK3CA
KRAS
ERBB2
NFE2L2
MAPK1
PPL
KLF5
ZNF423
Genes
conc
entr
hot12
hot3
Methods
Mutation freq
< 1%
1-2%
2-3%
3-4%
4-10%
> 10%

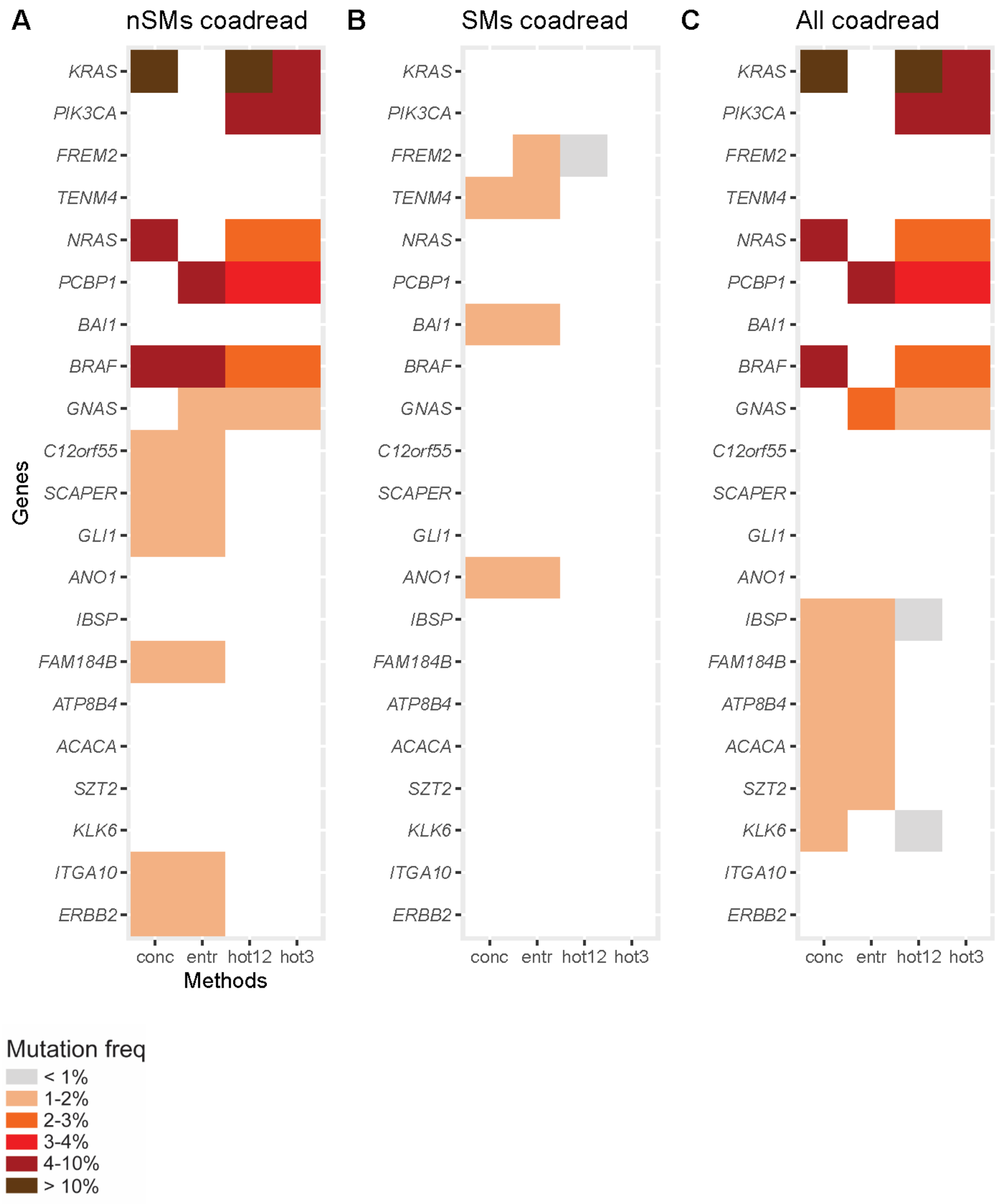
A
nSMs coadread
B
SMs coadread
C
All coadread
KRAS
PIK3CA
FREM2
TENM4
NRAS
PCBP1
BAI1
BRAF
GNAS
C12orf55
SCAPER
GLI1
ANO1
IBSP
FAM184B
ATP8B4
ACACA
SZT2
KLK6
ITGA10
ERBB2
Genes
conc
entr
hot12
hot3
Methods
Mutation freq
< 1%
1-2%
2-3%
3-4%
4-10%
> 10%

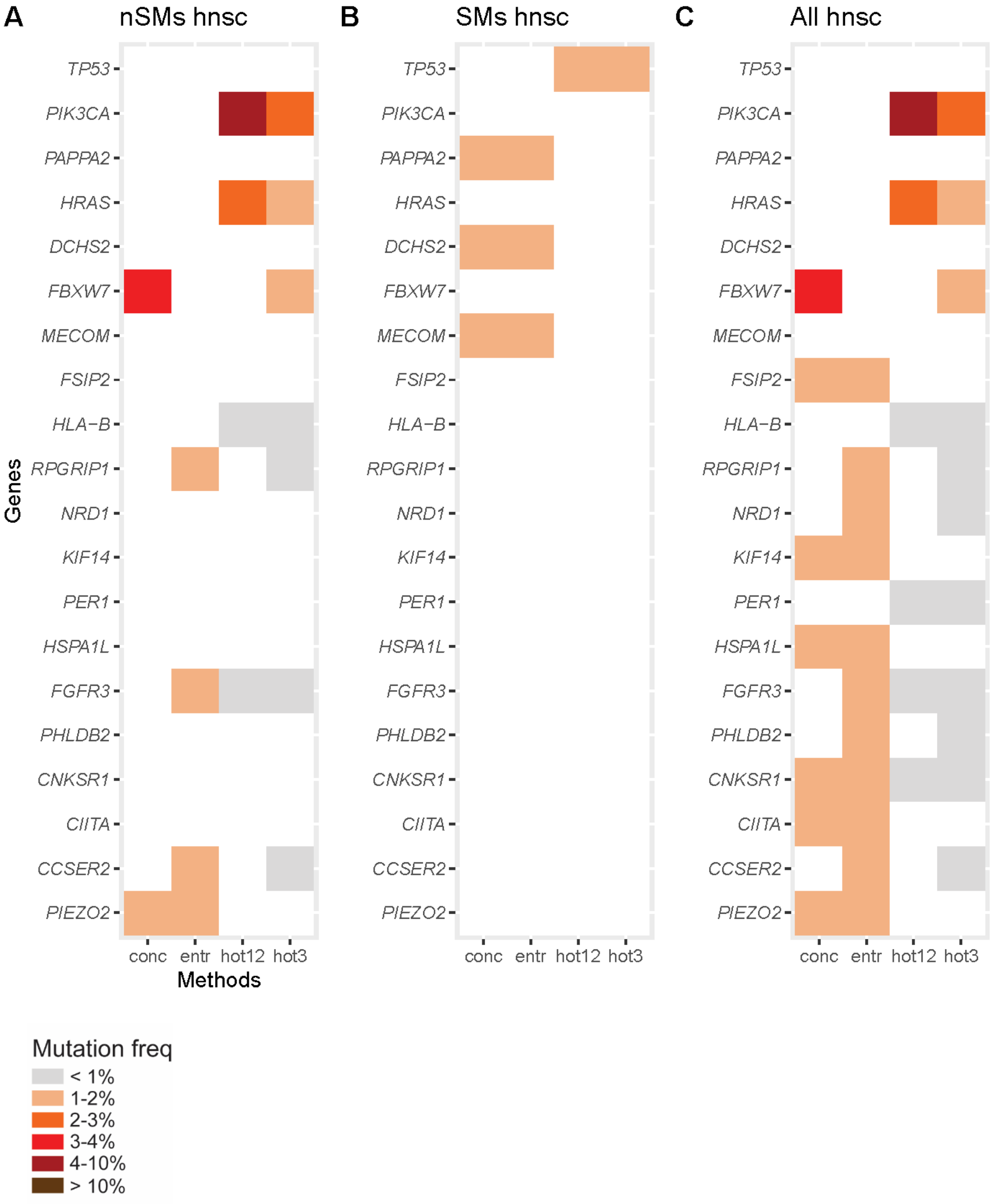
A
nSMs hnsc
B
SMs hnsc
C
All hnsc
TP53
PIK3CA
PAPPA2
HRAS
DCHS2
FBXW7
MECOM
FSIP2
HLA−B
RPGRIP1
NRD1
KIF14
PER1
HSPA1L
FGFR3
PHLDB2
CNKSR1
CIITA
CCSER2
PIEZO2
Genes
conc
entr
hot12
hot3
Methods
Mutation freq
< 1%
1-2%
2-3%
3-4%
4-10%
> 10%

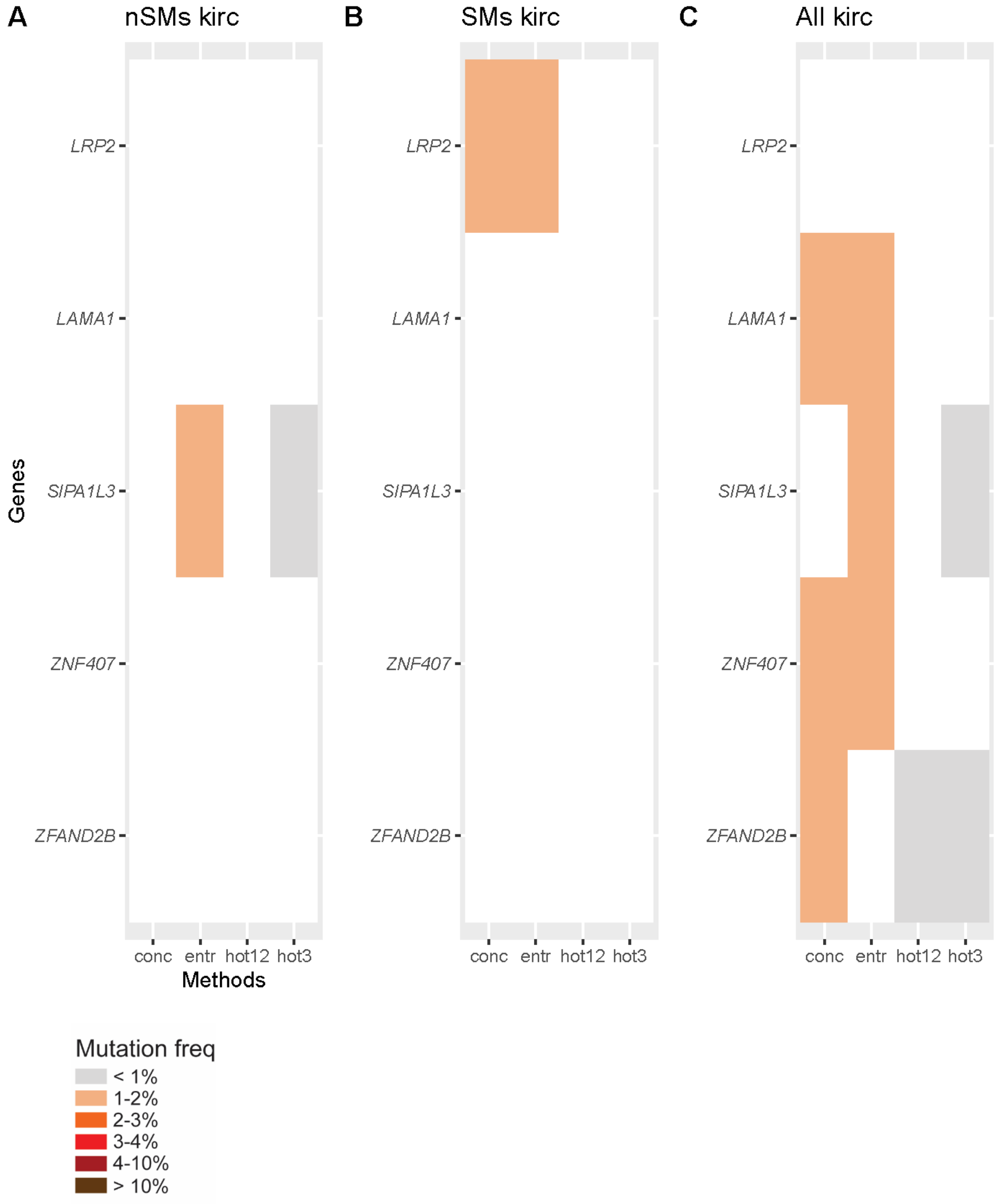

A
nSMs kirc
B
SMs kirc
C
All kirc
Genes
LRP2
LAMA1
SIPA1L3
ZNF407
ZFAND2B
conc
entr
hot12
hot3
Methods
Mutation freq
< 1%
1-2%
2-3%
3-4%
4-10%
> 10%

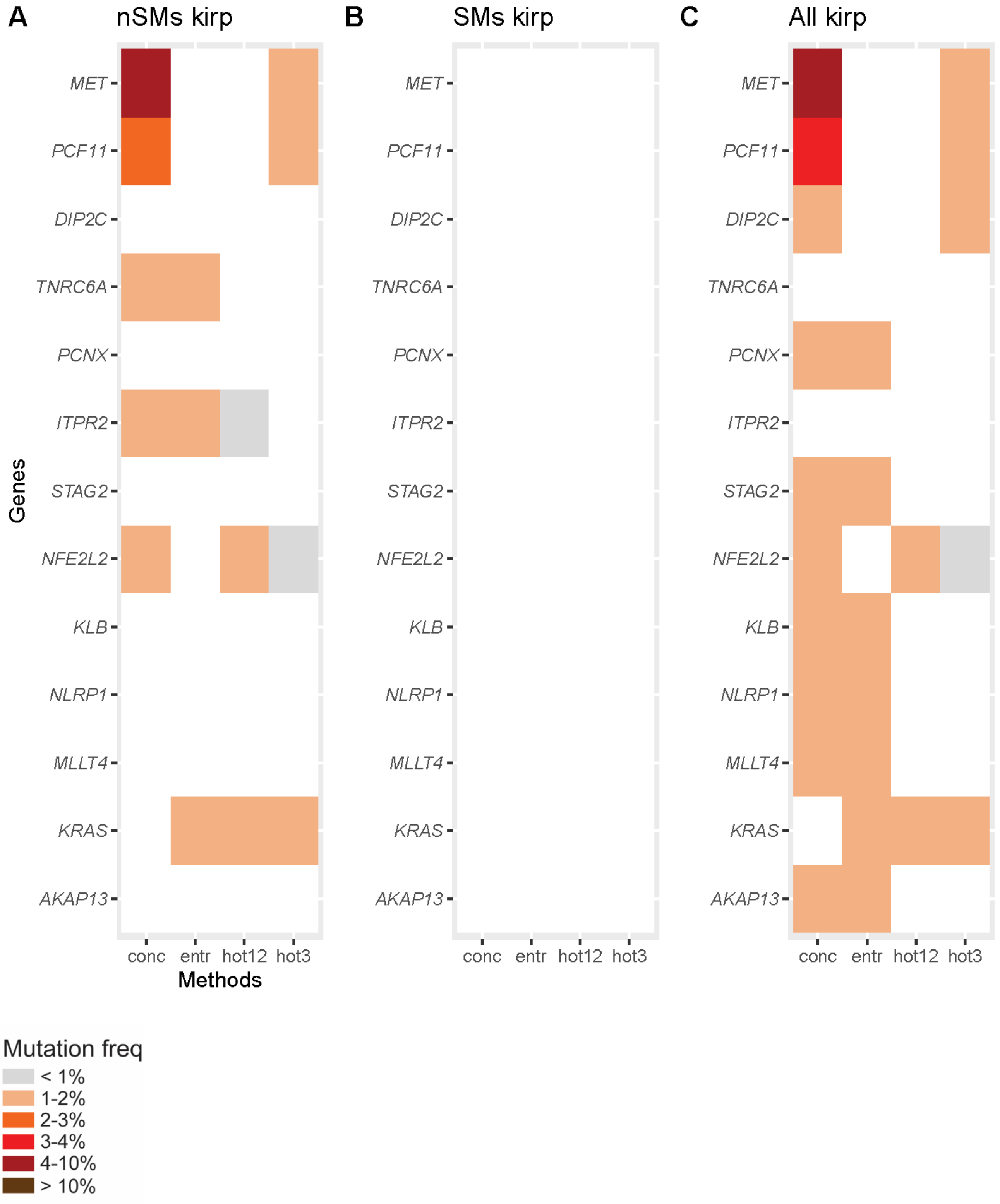
A
nSMs kirp
B
SMs kirp
C
All kirp
Genes
MET
PCF11
DIP2C
TNRC6A
PCNX
ITPR2
STAG2
NFE2L2
KLB
NLRP1
MLLT4
KRAS
AKAP13
conc
entr
hot12
hot3
Methods
Mutation freq
< 1%
1-2%
2-3%
3-4%
4-10%
> 10%

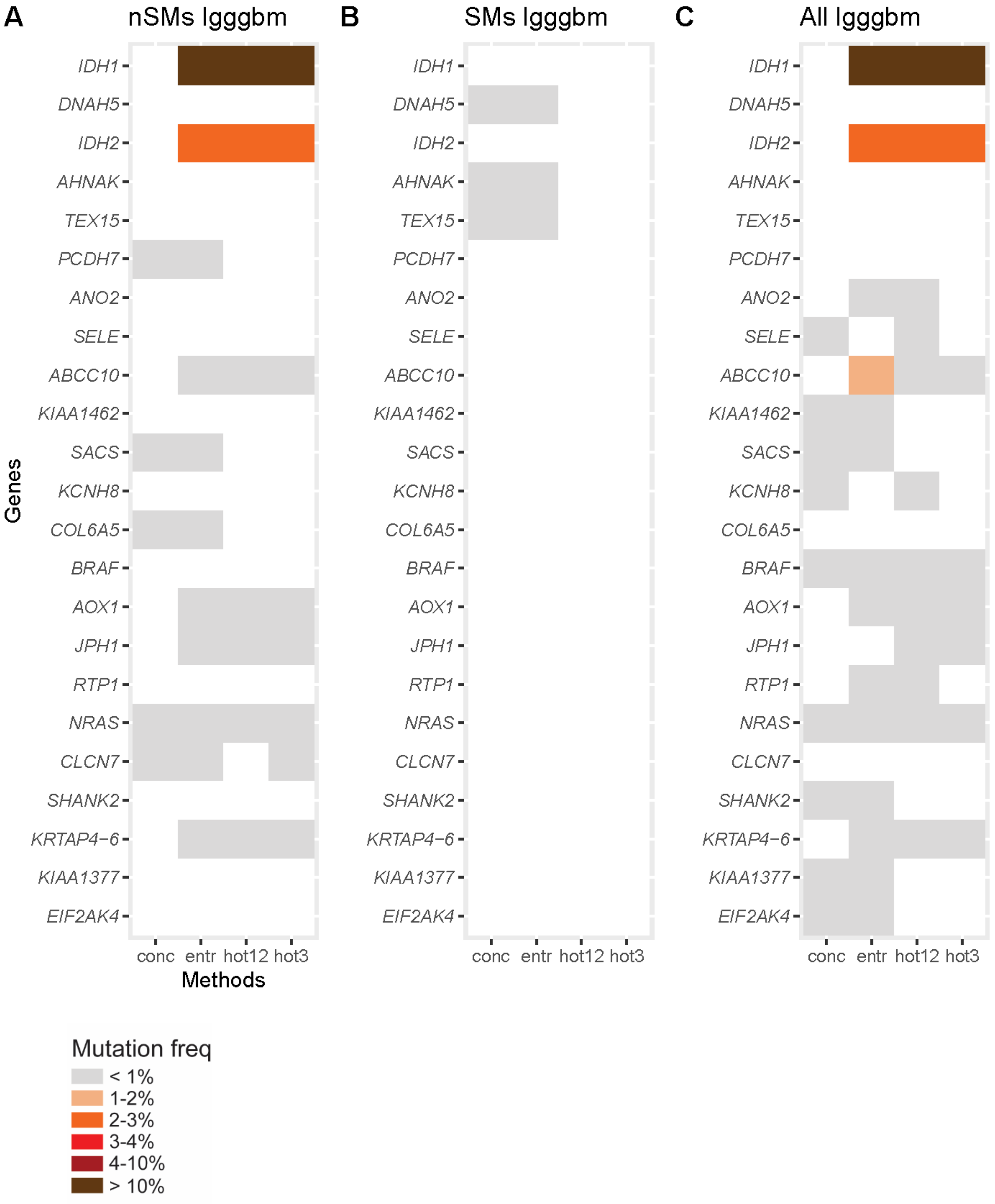
A
nSMs lgggbm
B
SMs lgggbm
C
All lgggbm
Genes
IDH1
DNAH5
IDH2
AHNAK
TEX15
PCDH7
ANO2
SELE
ABCC10
KIAA1462
SACS
KCNH8
COL6A5
BRAF
AOX1
JPH1
RTP1
NRAS
CLCN7
SHANK2
KRTAP4−6
KIAA1377
EIF2AK4
conc
entr
hot12
hot3
Methods
Mutation freq
< 1%
1-2%
2-3%
3-4%
4-10%
> 10%

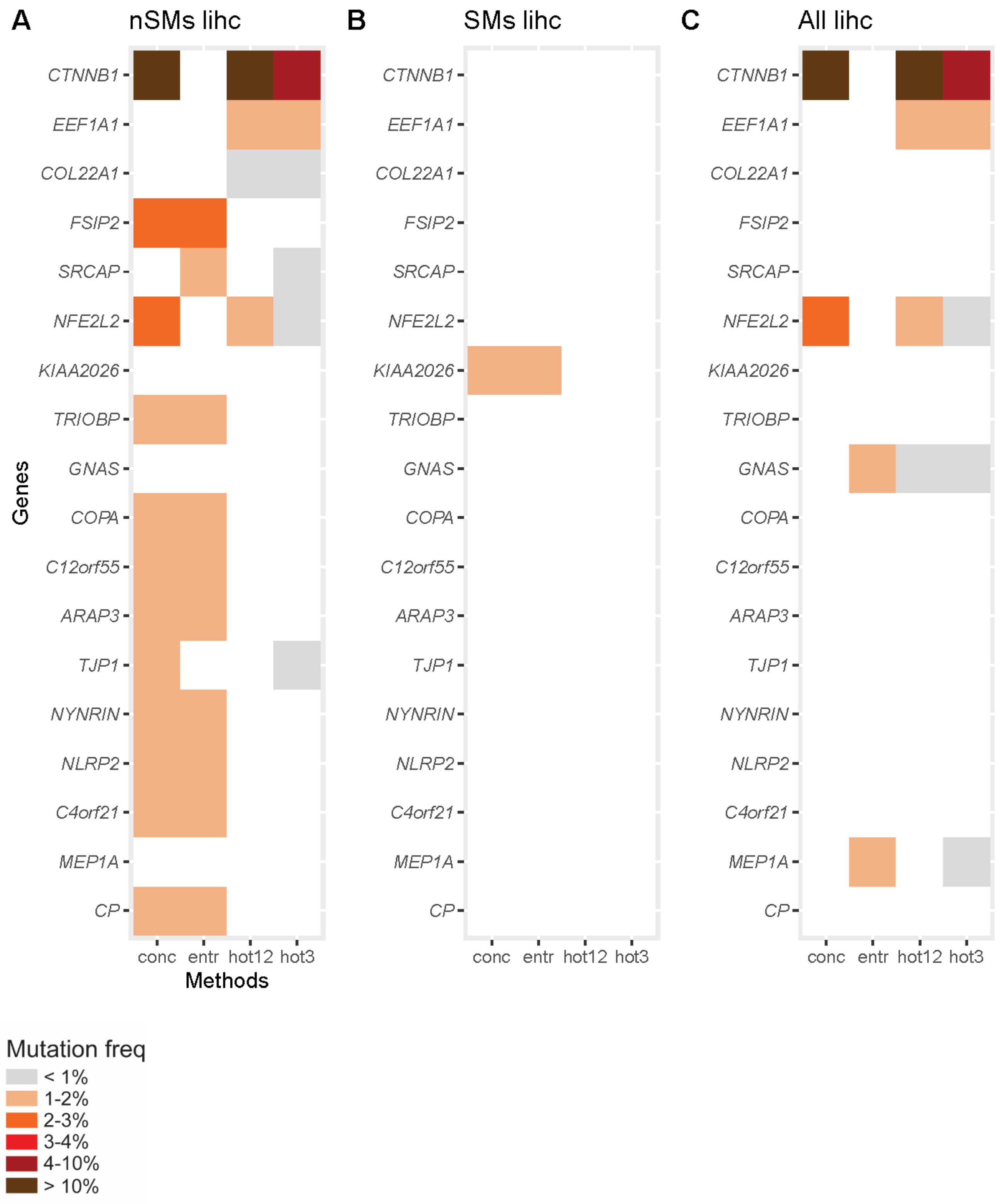

A
nSMs lihc
B
SMs lihc
C
All lihc
CTNNB1
EEF1A1
COL22A1
FSIP2
SRCAP
NFE2L2
KIAA2026
TRIOBP
GNAS
COPA
C12orf55
ARAP3
TJP1
NYNRIN
NLRP2
C4orf21
MEP1A
CP
Genes
conc
entr
hot12
hot3
Methods
Mutation freq
< 1%
1-2%
2-3%
3-4%
4-10%
> 10%

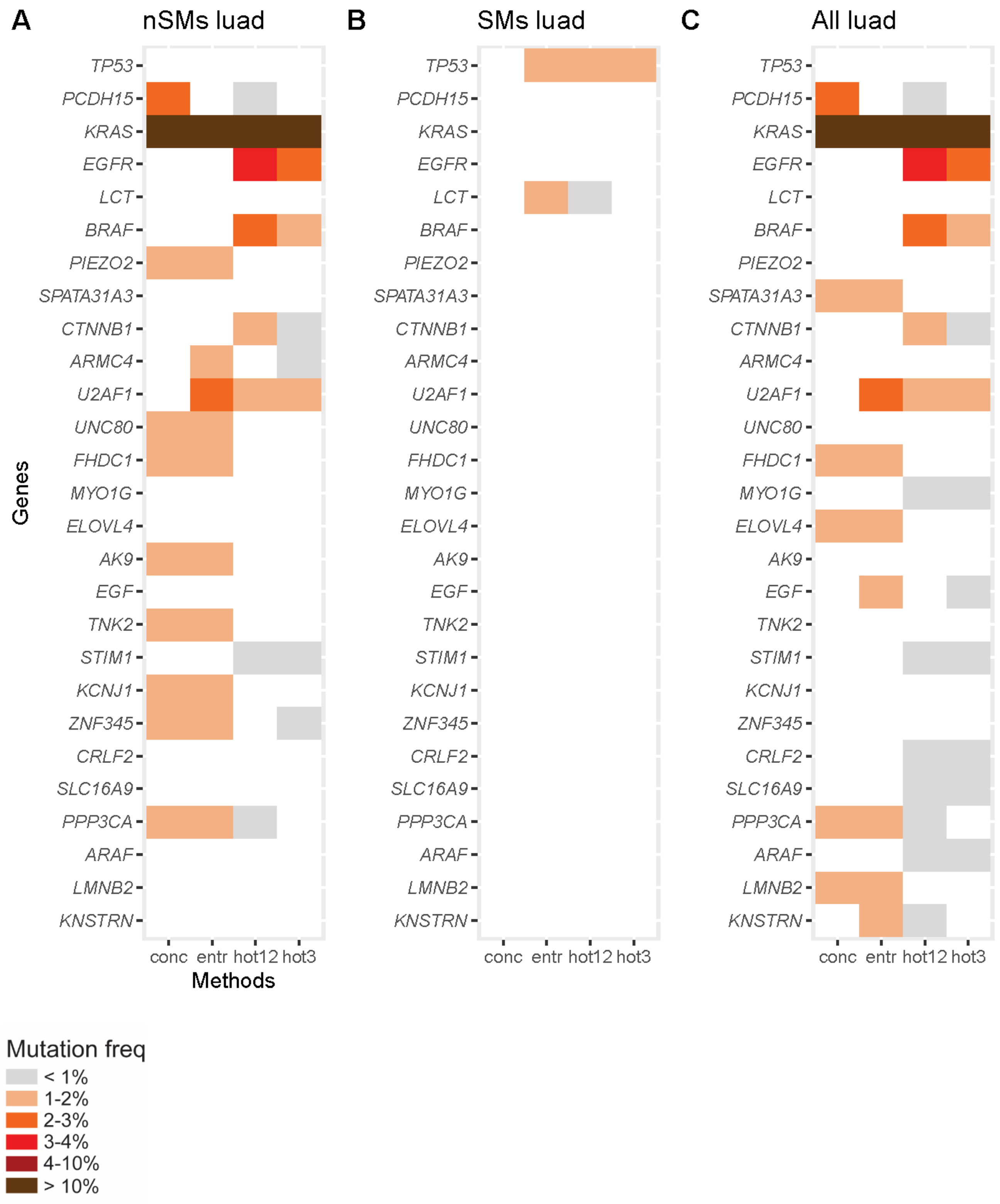

A
nSMs luad
B
SMs luad
C
All luad
Genes
TP53
PCDH15
KRAS
EGFR
LCT
BRAF
PIEZO2
SPATA31A3
CTNNB1
ARMC4
U2AF1
UNC80
FHDC1
MYO1G
ELOVL4
AK9
EGF
TNK2
STIM1
KCNJ1
ZNF345
CRLF2
SLC16A9
PPP3CA
ARAF
LMNB2
KNSTRN
conc
entr
hot12
hot3
Methods
Mutation freq
< 1%
1-2%
2-3%
3-4%
4-10%
> 10%

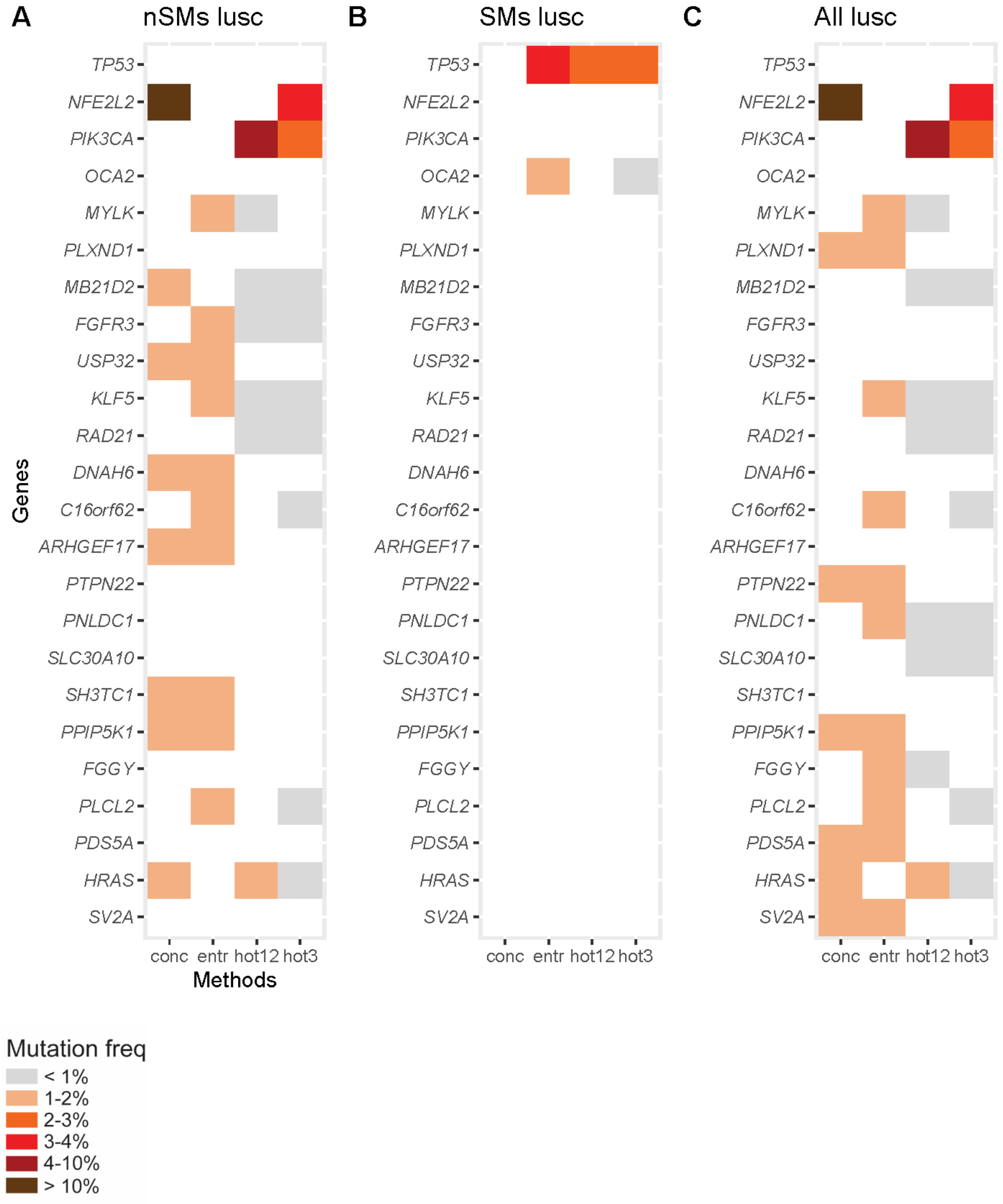
A
nSMs lusc
B
SMs lusc
C
All lusc
TP53
NFE2L2
PIK3CA
OCA2
MYLK
PLXND1
MB21D2
FGFR3
USP32
KLF5
RAD21
DNAH6
C16orf62
ARHGEF17
PTPN22
PNLDC1
SLC30A10
SH3TC1
PPIP5K1
FGGY
PLCL2
PDS5A
HRAS
SV2A
Genes
conc
entr
hot12
hot3
Methods
Mutation freq
< 1%
1-2%
2-3%
3-4%
4-10%
> 10%

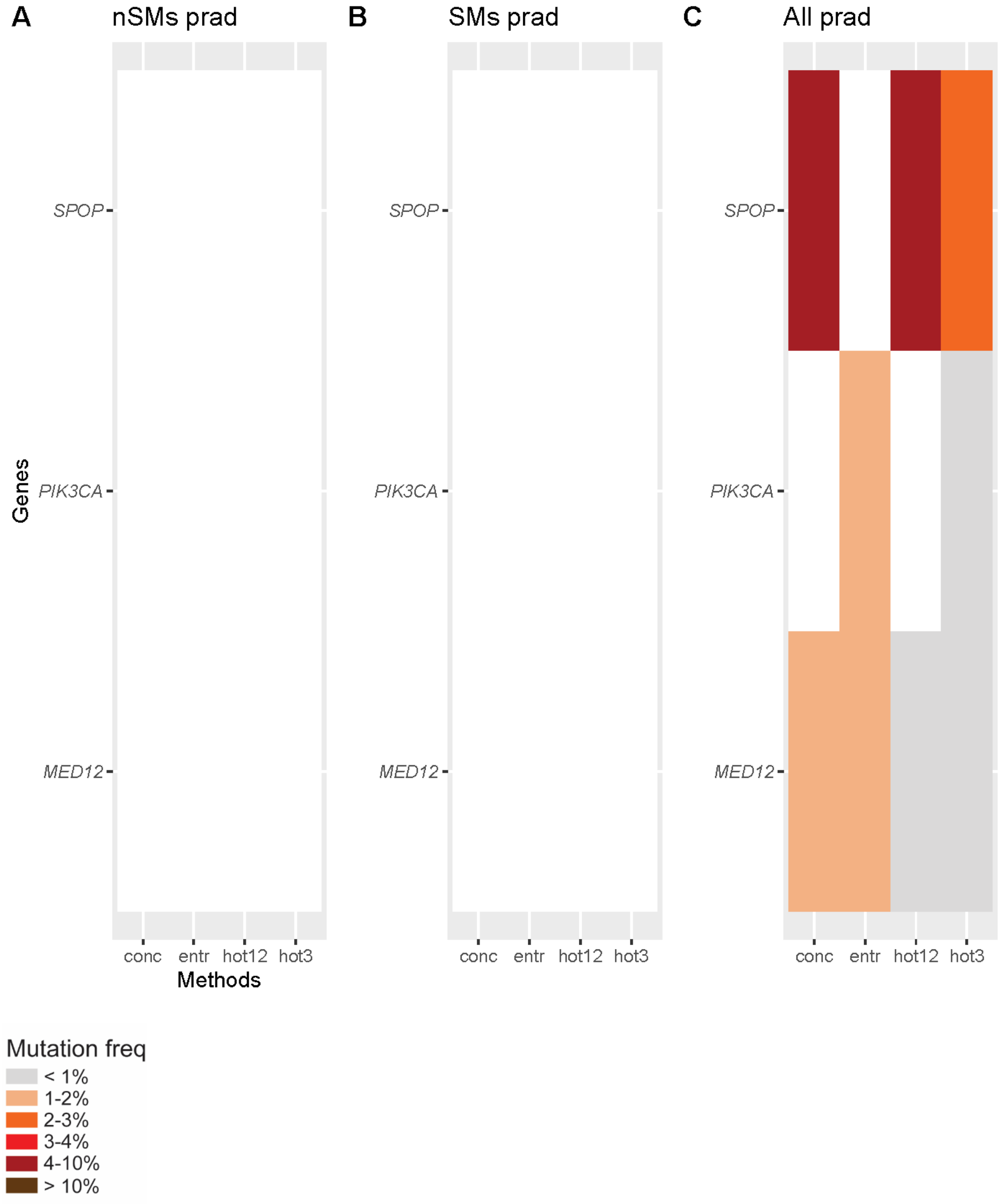
A
nSMs prad
B
SMs prad
C
All prad
SPOP
PIK3CA
MED12
Genes
conc
entr
hot12
hot3
Methods
Mutation freq
< 1%
1-2%
2-3%
3-4%
4-10%
> 10%

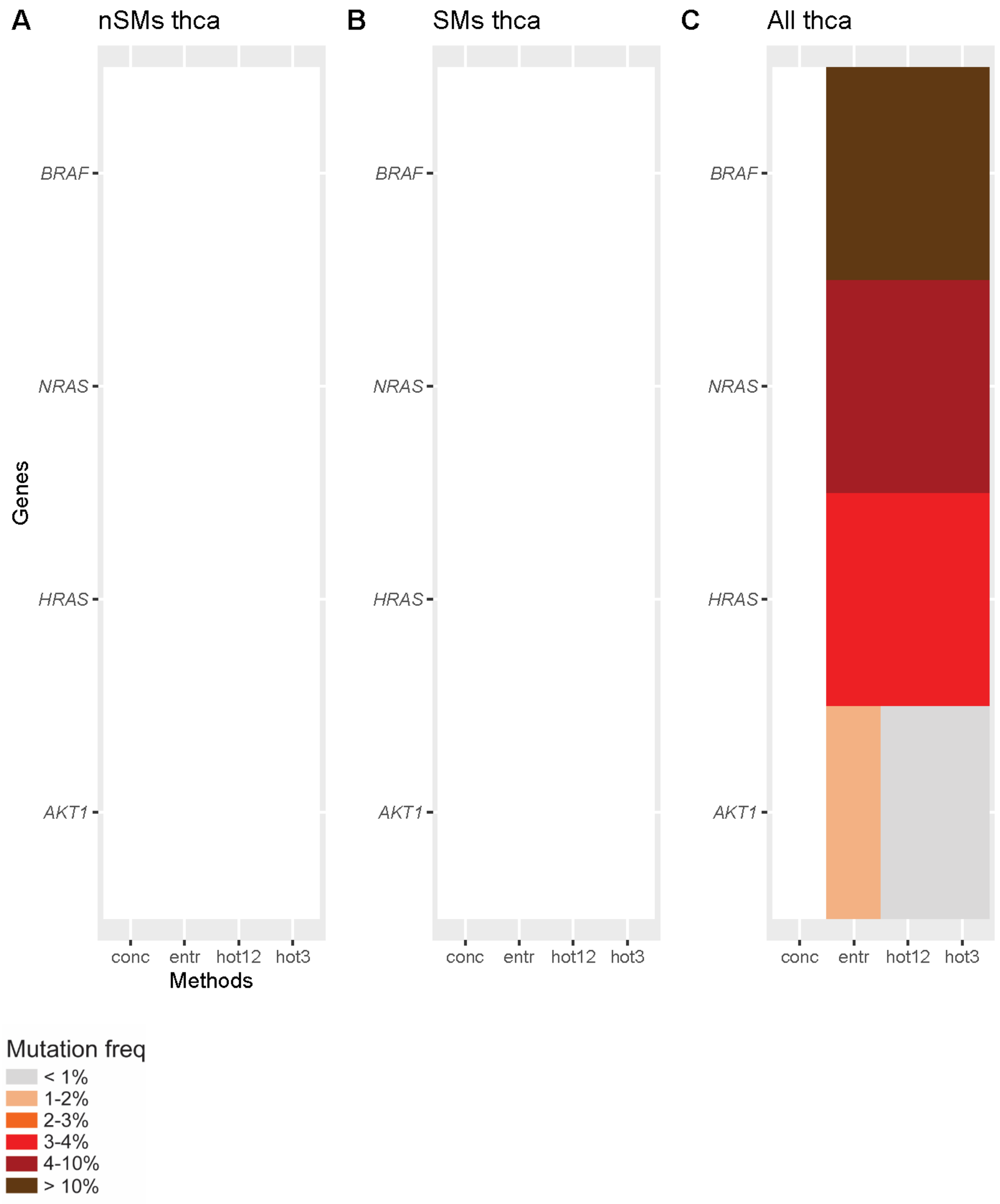
A
nSMs thca
B
SMs thca
C
All thca
BRAF
NRAS
HRAS
AKT1
Genes
conc
entr
hot12
hot3
Methods
Mutation freq
< 1%
1-2%
2-3%
3-4%
4-10%
> 10%

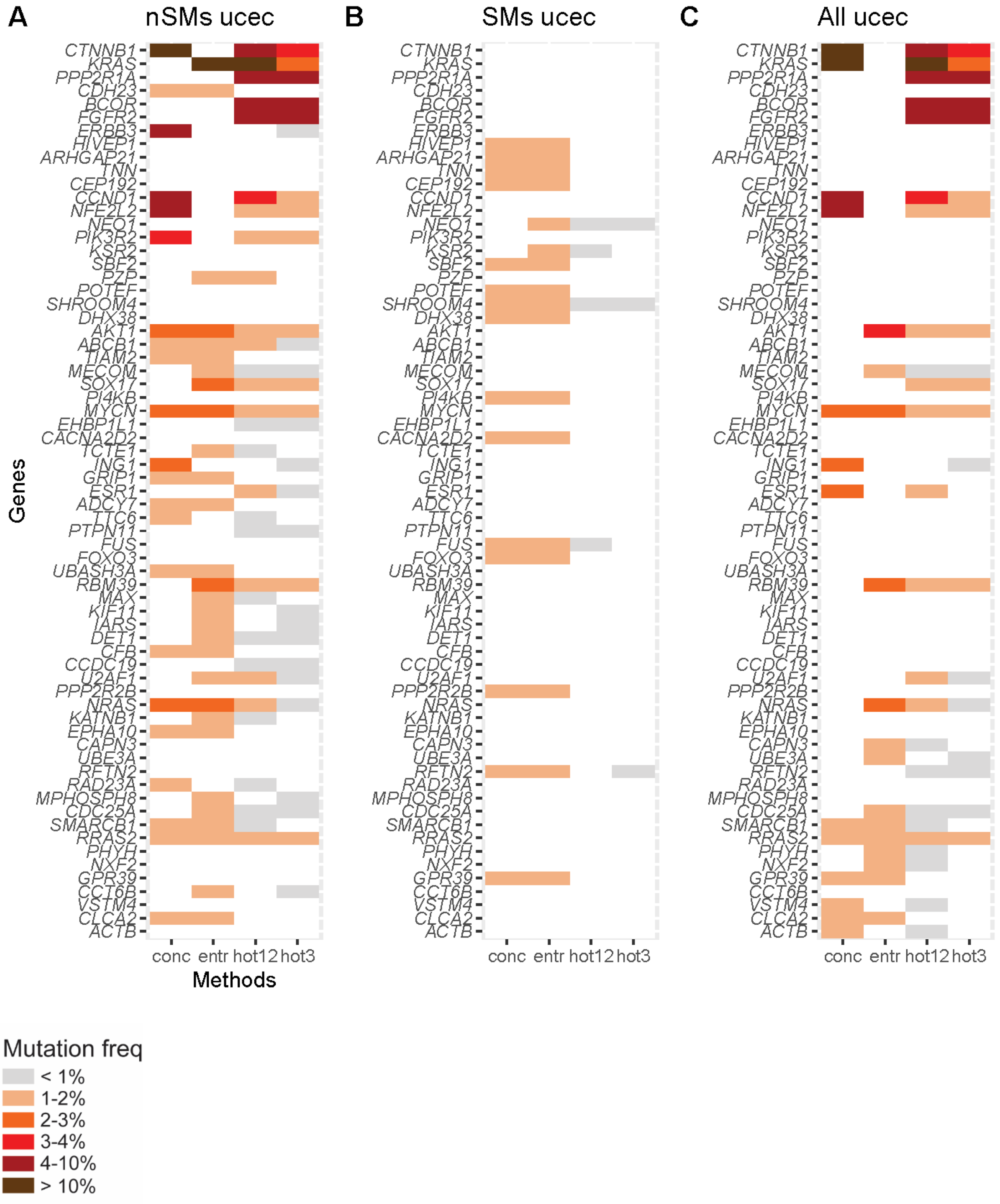
A
nSMs ucec
B
SMs ucec
C
All ucec
Genes
CTNNB1
KRAS
PPP2R1A
CDH23
BCOR
FGFR2
ERBB3
HIVEP1
ARHGAP21
TNN
CEP192
CCND1
NFE2L2
NEO1
PIK3R2
KSR2
SBF2
PZP
POTEF
SHROOM4
DHX38
AKT1
ABCB1
TIAM2
MECOM
SOX17
PI4KB
MYCN
EHBP1L1
CACNA2D2
TCTE1
ING1
GRIP1
ESR1
ADCY7
TTC6
PTPN11
FUS
FOXO3
UBASH3A
RBM39
MAX
KIF11
IARS
DET1
CFB
CCDC19
U2AF1
PPP2R2B
NRAS
KATNB1
EPHA10
CAPN3
UBE3A
RFTN2
RAD23A
MPHOSPH8
CDC25A
SMARCB1
RRAS2
PHYH
NXF2
GPR39
CCT6B
VSTM4
CLCA2
ACTB
conc
entr
hot12
hot3
Methods
Mutation freq
< 1%
1-2%
2-3%
3-4%
4-10%
> 10%

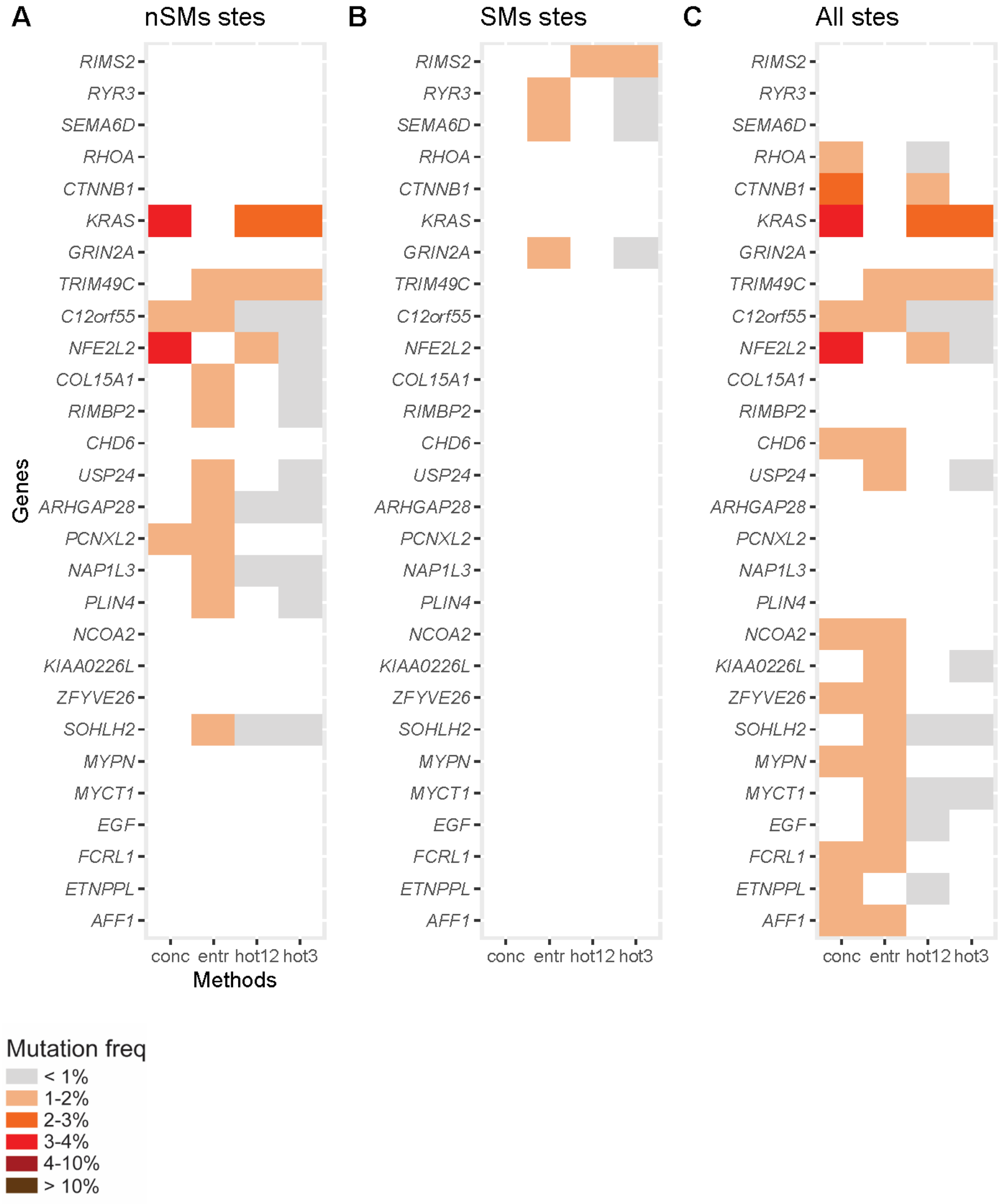
A
nSMs stes
B
SMs stes
C
All stes
Genes
RIMS2
RYR3
SEMA6D
RHOA
CTNNB1
KRAS
GRIN2A
TRIM49C
C12orf55
NFE2L2
COL15A1
RIMBP2
CHD6
USP24
ARHGAP28
PCNXL2
NAP1L3
PLIN4
NCOA2
KIAA0226L
ZFYVE26
SOHLH2
MYPN
MYCT1
EGF
FCRL1
ETNPPL
AFF1
conc
entr
hot12
hot3
Methods
Mutation freq
< 1%
1-2%
2-3%
3-4%
4-10%
> 10%

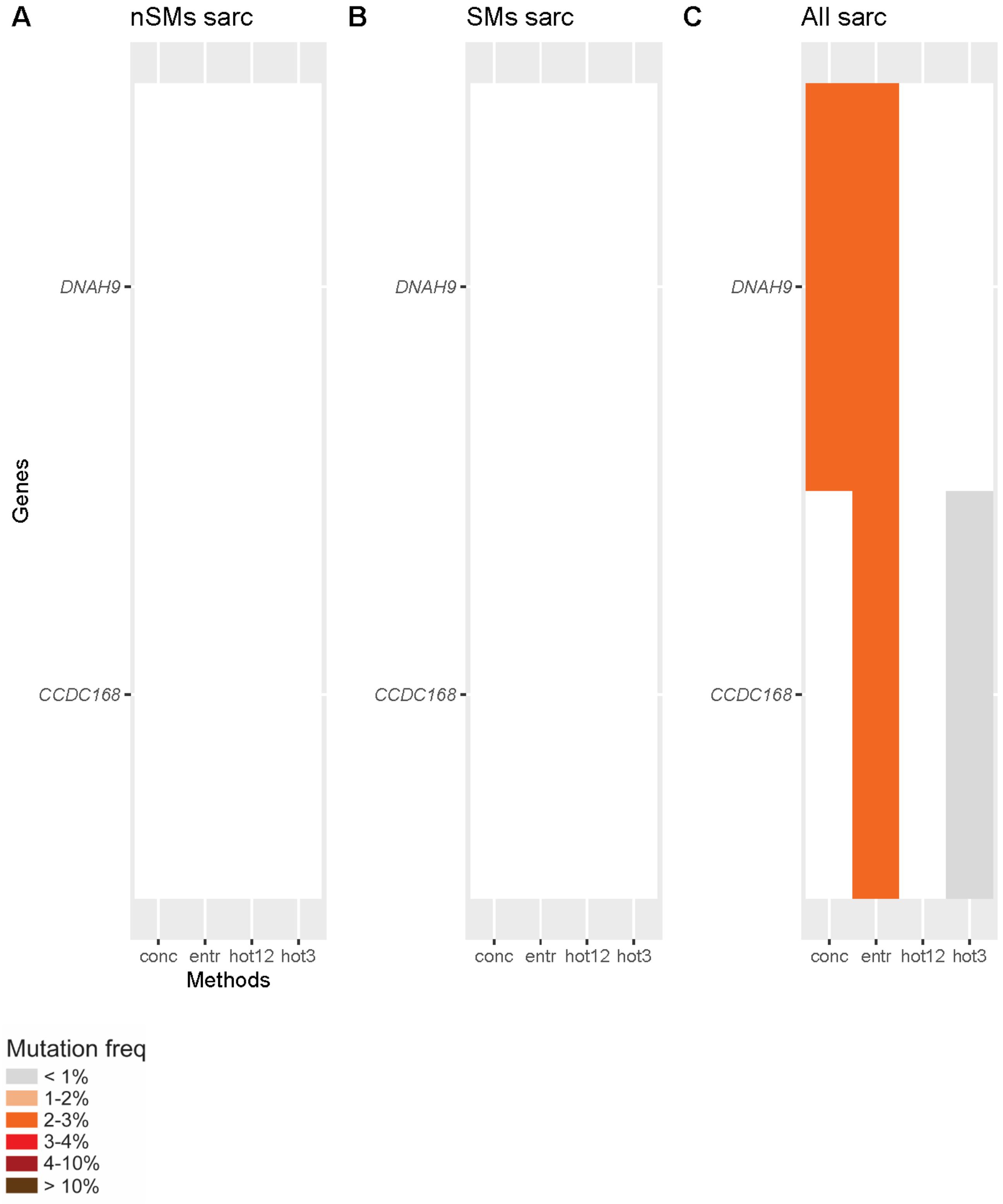

Figure S2. Heatmaps summarizing significant mutation clusters in 16 analysed tumor types. Only genes that were significant for at least two concentration methods for nSMs, SMs, or All mutations are depicted in these heatmaps. All genes with significant mutation clusters are reported in Supplementary Tables 2-5. Note: results obtained for SKCM, the 17th tumor type that was analysed, are depicted in Figure 1B.

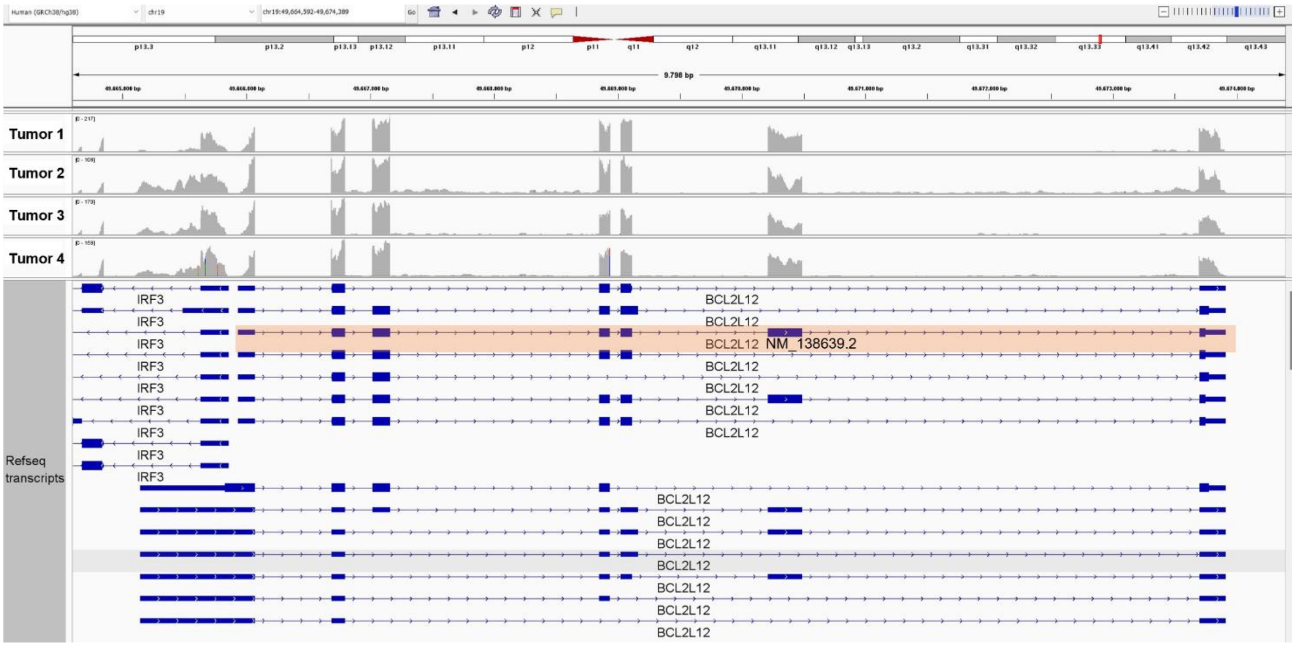

Figure S3. NM_138639.2 is the main expressed *BCL2L12* transcript in SKCM tumors. RNA-seq data from four representative SKCM tumors were mapped against hg38 in Integrative Genomics Viewer (IGV). The image shows that the reads covering *BCL2L12* mainly correspond to *BCL2L12* transcript NM_138639.2, which was also confirmed by Salmon[1] analysis.

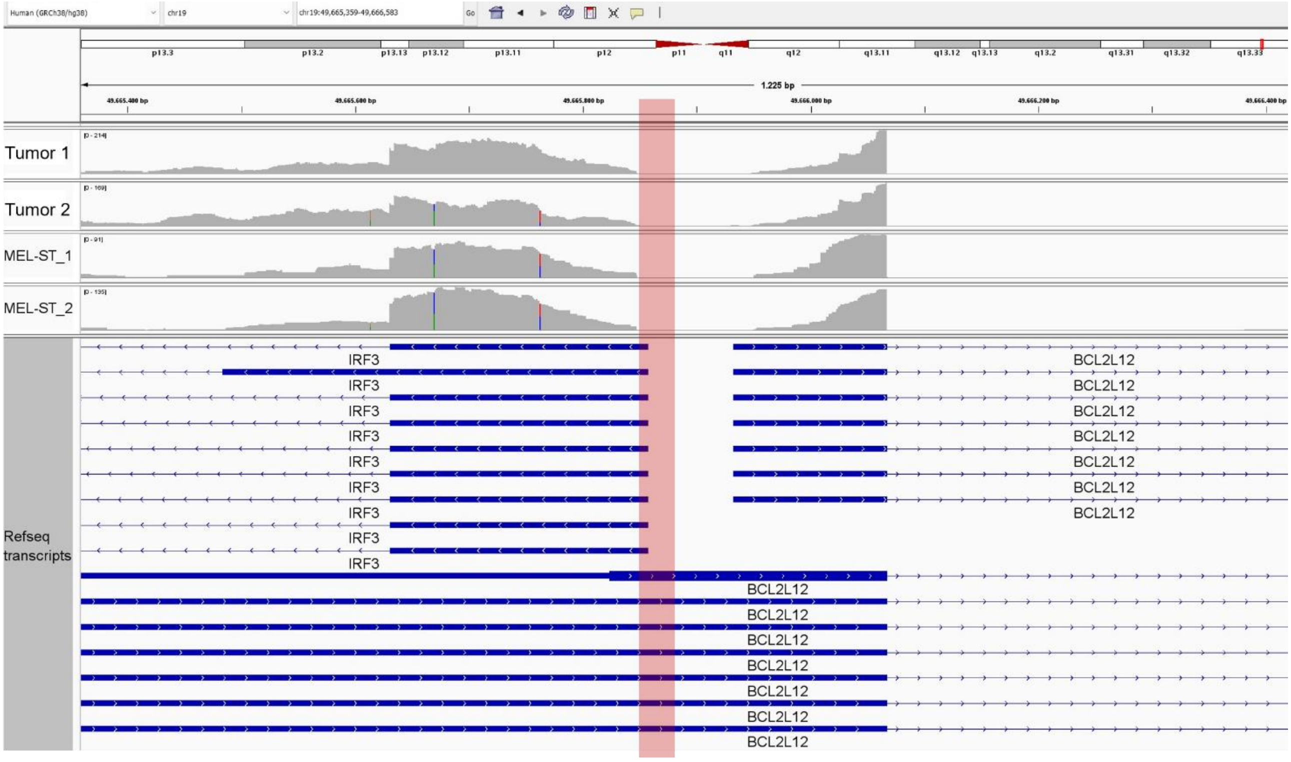

Figure S4. Mel-ST primary melanocytes express the same *BCL2L12* and *IRF3* transcripts as SKCM tumors. Illumina based RNA-seq data from two melanoma tumors (tumor 1 and tumor 2) and from Mel-ST cells (two replicates indicated by _1 and _2) were mapped against hg38 in IGV. The RNA-seq read distribution plot in the *IRF3/BCL2L12* promoter region is shown, and the region in which *IRF3/BCL2L12* promoter mutations were identified in SKCM tumors is indicated with a red shaded box. The figure shows that SKCM tumors and Mel-ST cells express the same *BCL2L12* and *IRF3* transcripts, and that the identified mutations represent *IRF3/BCL2L12* promoter mutations in SKCM tumors and in Mel-ST cells.

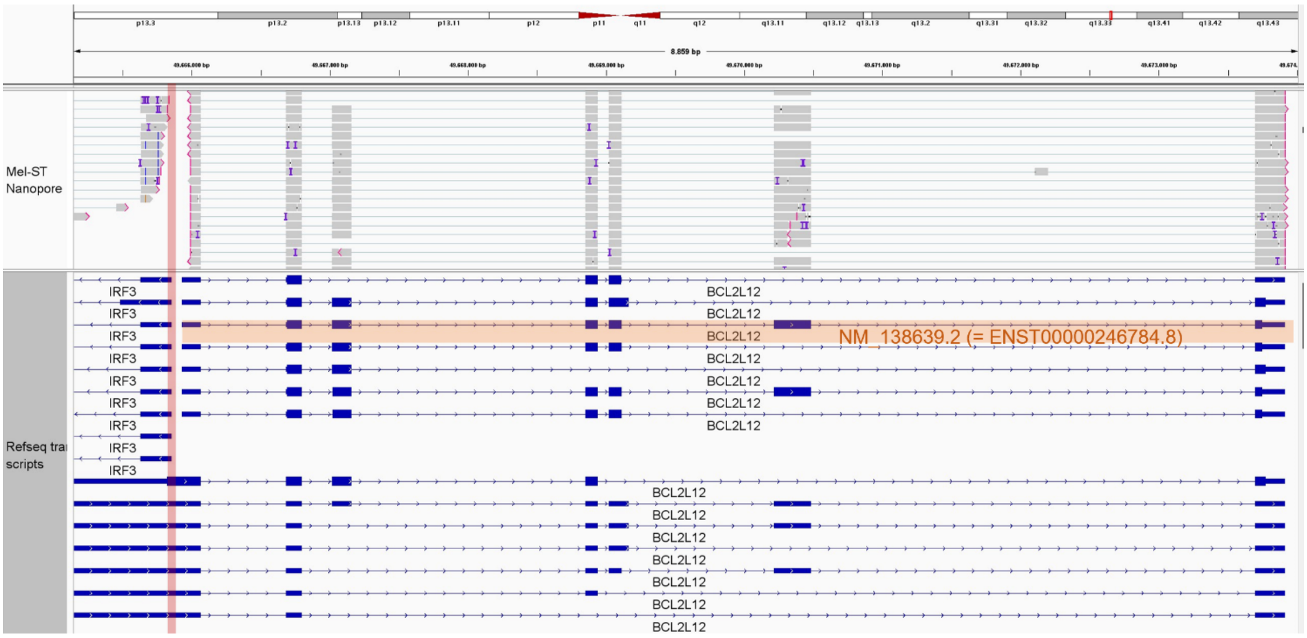

Figure S5. Confirmation of main expressed *BCL2L12* transcript by Oxford Nanopore Technology (ONT) Sequencing on Mel-ST cells. IGV analysis showing mapping of ONT direct cDNA sequencing reads, confirming that NM_138639.2 (or ENST00000246784.8) is the main expressed *BCL2L12* isoform in Mel-ST cells. The position of the *IRF3/BCL2L12* promoter mutations is indicated by the red rectangle.

A

NCBI_ID	Protein size (kDa)
NM_0010040668.2	27
NM_138639.2	27
NM_001282520.1	25
NM_001282519.2	20
NM_001282516.2	17
NM_001385706.1	17
NM_001282521.2	11
NM_001282517.2	9
NR_104201.1	-
NR_104202.1	-
NR_104203.1	-
NR_104200.1	-
NR_104204.1	-
NR_104205.1	-

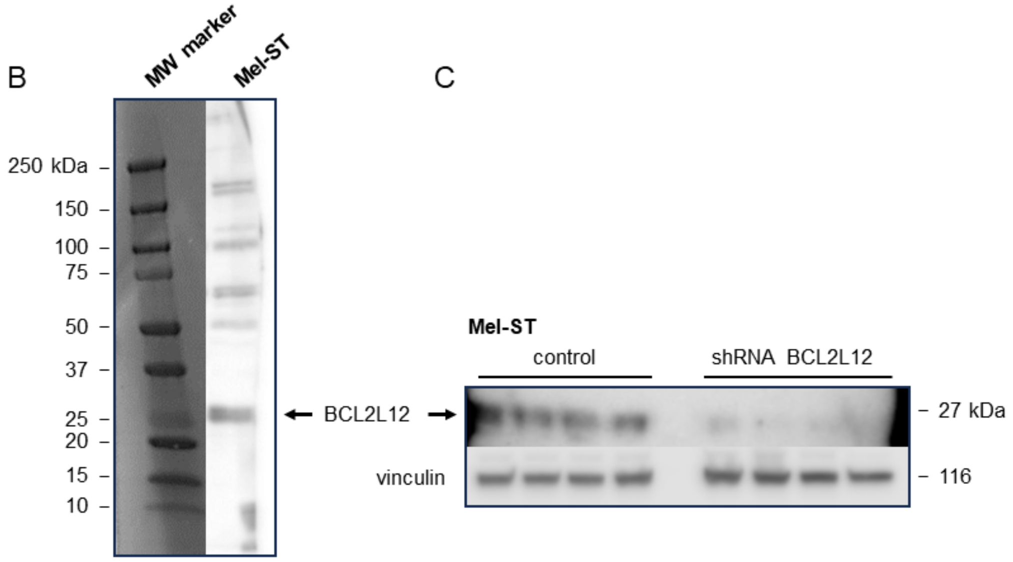

Figure S6. Western blot showing BCL2L12 protein MW fitting with expressed RNA isoform NM_138639.2. A) Overview of different BCL2L12 isoforms and corresponding protein MW. Transcript NM_138639.2, the main expressed RNA isoform (Figure S3) corresponds to a protein of MW 27kDa. **B)** Western blot analysis of BCL2L12 in Mel-ST cells. The indicated band on the western blot matches the 27kDa MW of the protein corresponding to NM_138639.2. The signals of higher MW that are detected are likely aspecific signals, as their MW is higher than all reported BCL2L12 proteoforms. **C)** Four different shRNAs were used to knock down BCL2L12 in Mel-ST cells, showing disappearance of the 27kDa protein band.

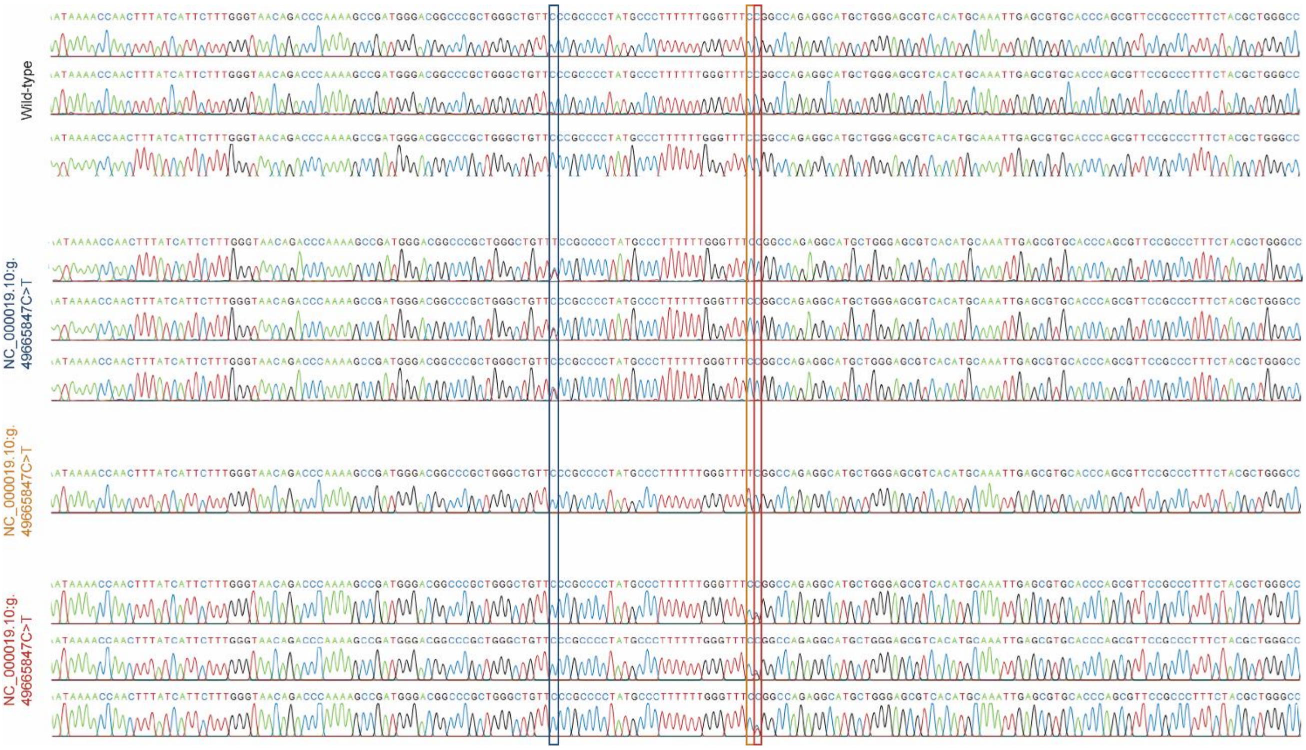

Figure S7. Sanger sequence validation of CRISPR-Cas9 engineered knock-in mutations NC_000019.10:g.49668547C>T, NC_000019.10:g.49668574C>T and NC_000019.10: g.49668575C>T. Each shown chromatogram corresponds to a different single-cell-derived clone of the indicated genotype. The vertical boxes indicate the positions of the corresponding mutations. All mutant clones were heterozygous for the indicated mutations.

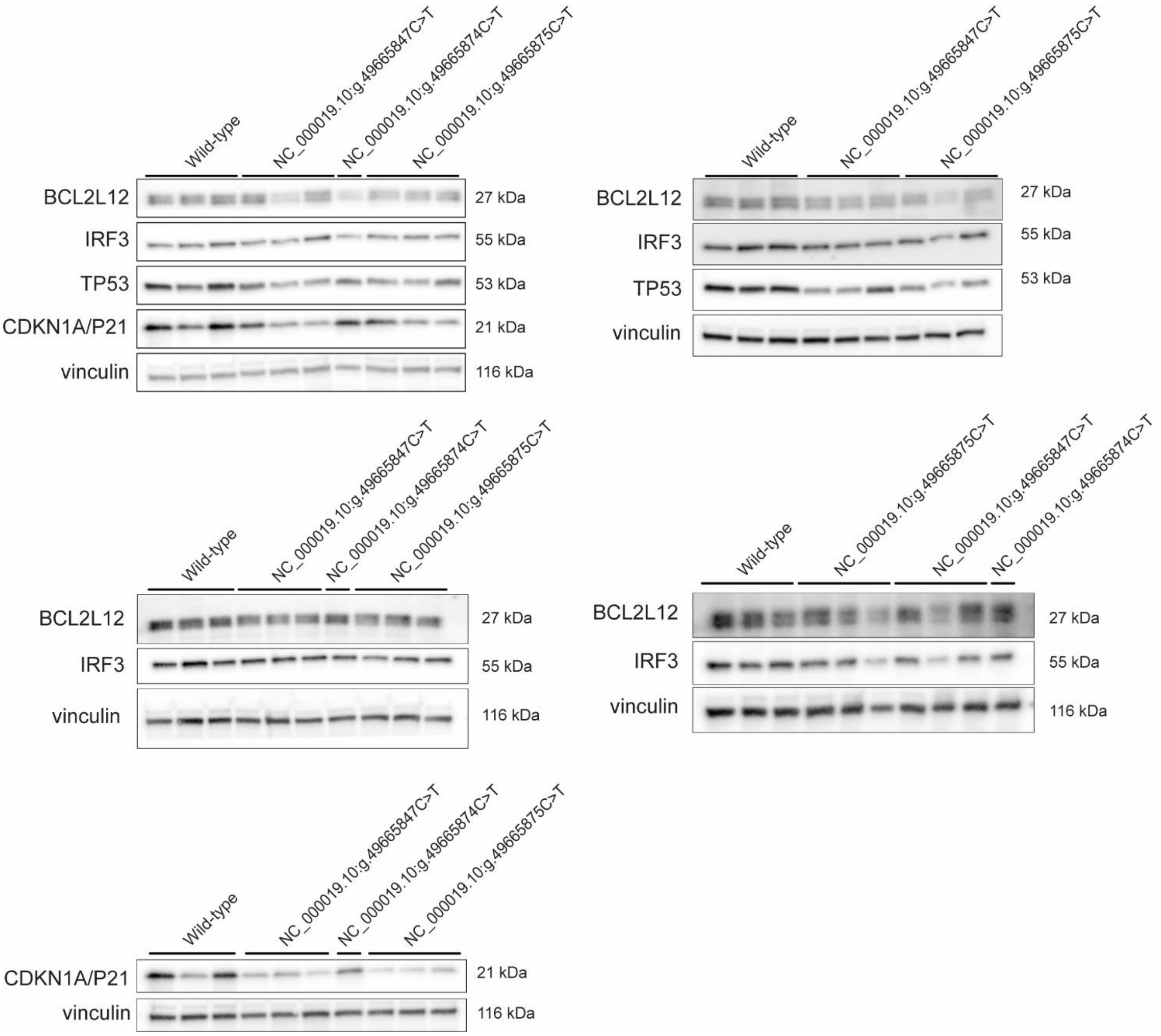

Figure S8. Additional western blotting replicates. Additional western blotting results that were included in the pooled data quantification in main Figure 3D and 3I.

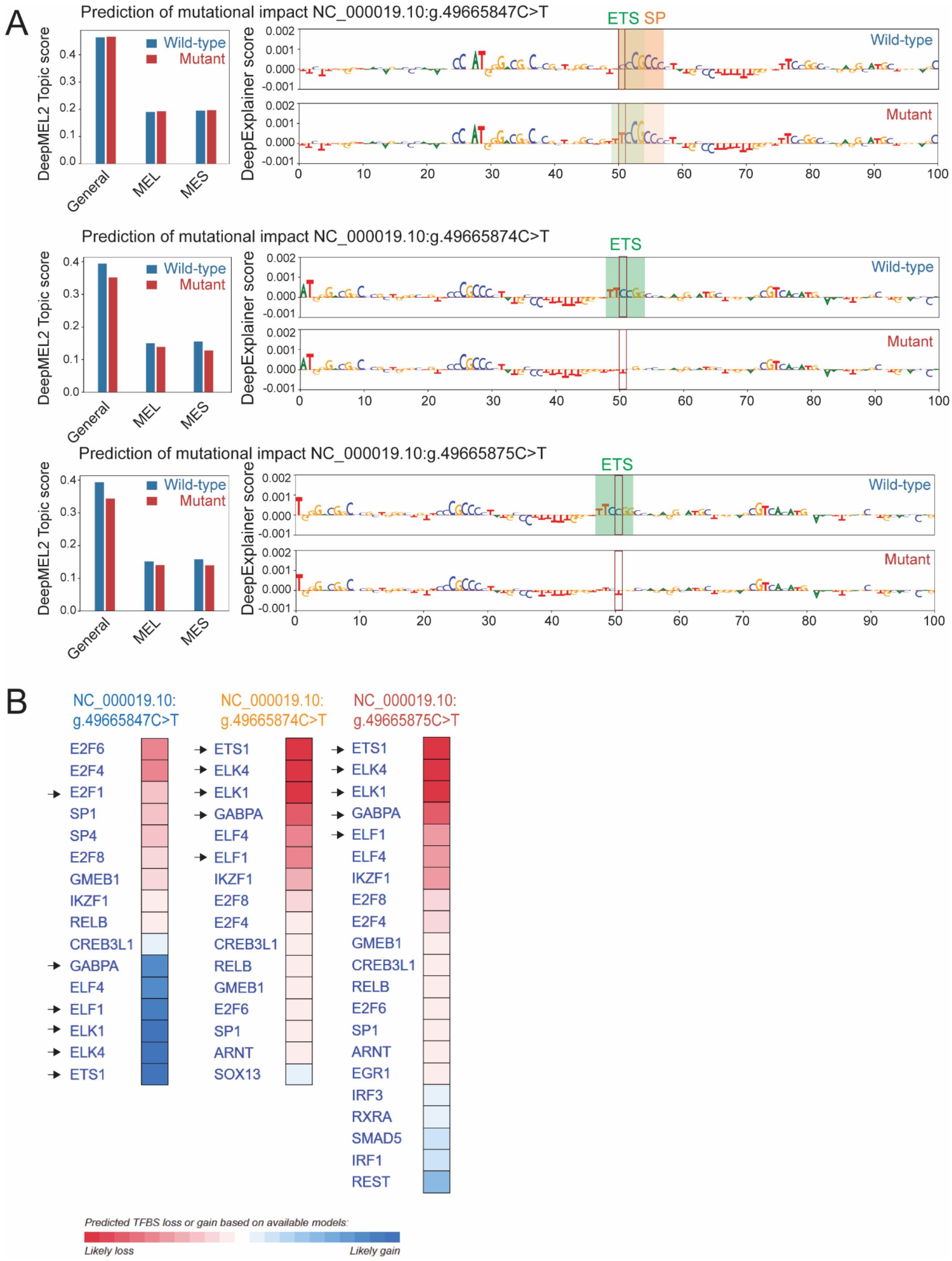
A
Prediction of mutational impact NC_000019.10:g.49665847C>T
DeepMEL2 Topic score
Wild-type
Mutant
General
MEL
MES
DeepExplainer score
ETS
SP
Prediction of mutational impact NC_000019.10:g.49665874C>T
Prediction of mutational impact NC_000019.10:g.49665875C>T
B
NC_000019.10: g.49665847C>T
E2F6
E2F4
E2F1
SP1
SP4
E2F8
GMEB1
IKZF1
RELB
CREB3L1
GABPA
ELF4
ELF1
ELK1
ELK4
ETS1
NC_000019.10: g.49665874C>T
ETS1
ELK4
ELK1
GABPA
ELF4
ELF1
IKZF1
E2F8
E2F4
CREB3L1
RELB
GMEB1
E2F6
SP1
ARNT
SOX13
NC_000019.10: g.49665875C>T
ETS1
ELK4
ELK1
GABPA
ELF1
ELF4
IKZF1
E2F8
E2F4
GMEB1
CREB3L1
RELB
E2F6
SP1
ARNT
EGR1
IRF3
RXRA
SMAD5
IRF1
REST
Predicted TFBS loss or gain based on available models:
Likely loss
Likely gain

Figure S9. DeepMEL2, Physbinder and FABIAN-Variant prediction of the impact of *IRF3/BCL2L12* promoter mutations on transcription factor binding. A) Scoring of the three *IRF3/BCL2L12* promoter variants (NC_000019.10:g.49665847C>T, NC_000019.10:g.49665874C>T and NC_000019.10:g.49665875C>T) using DeepMEL2, a melanoma specific deep learning model to interpret how sequence variation affects promoter and enhancer activity and TF binding sites[2]. The DeepMEL2 predictions are based on training the model based on three classes/topics, which represent a general melanoma (general), a melanocytic (MEL), and a mesenchymal (MES) state. The visualisation on the right shows the nucleotide targeted by the mutation (indicated by a red rectangle), as well as the 50 nucleotides upstream and downstream, and illustrates the creation (NC_000019.10:g.49665847C>T) or disruption (NC_000019.10:g.49665874C>T and NC_000019.10:g.49665875C>T) of ETS transcription factor binding sites that underlie the observed prediction differences. Green shaded areas indicate an ETS binding site. **B)** Predicted effect of NC_000019.10:g.49665847C>T, NC_000019.10:g.49665874C>T and NC_000019.10:g.49665875C>T on transcription factor binding using FABIAN-variant[3]. Results corroborated PhysBinder[4] are indicated with an arrow.

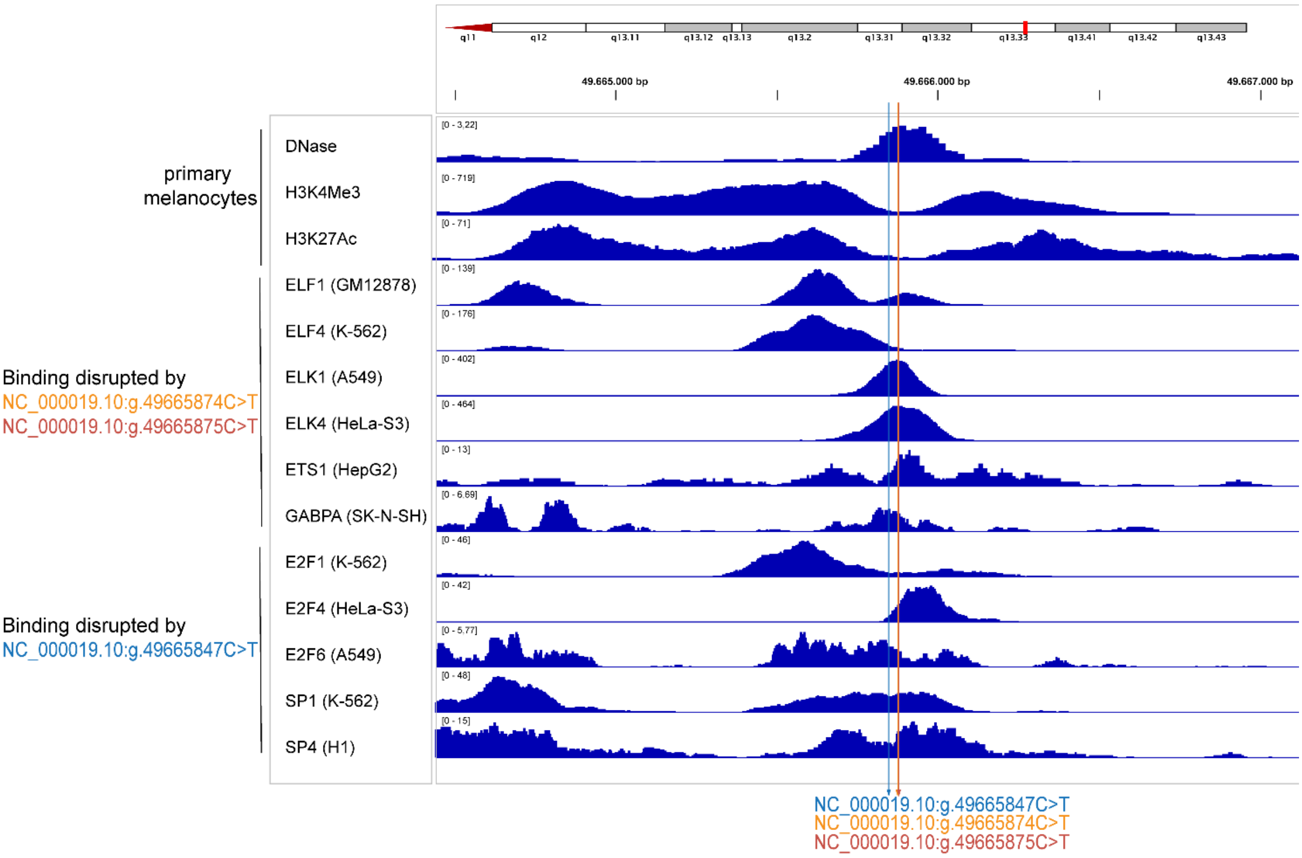

Figure S10. The *IRF3/BCL2L12* promoter mutations are in an open active chromatin region that can be bound by ETS, E2F and SP family transcription factors. ENCODE DNase-seq and ChIP-seq data for H3K4Me3 and H3K27Ac indicate open chromatin and an active *IRF3/BCL2L12* promoter region in wild-type primary melanocytes. ENCODE ChIP-seq data show binding peaks of the transcription factors whose binding is predicted to be disrupted by the *IRF3/BCL2L12* promoter mutations. The cell lines in which these ChIP-seq data were obtained are indicated between brackets.

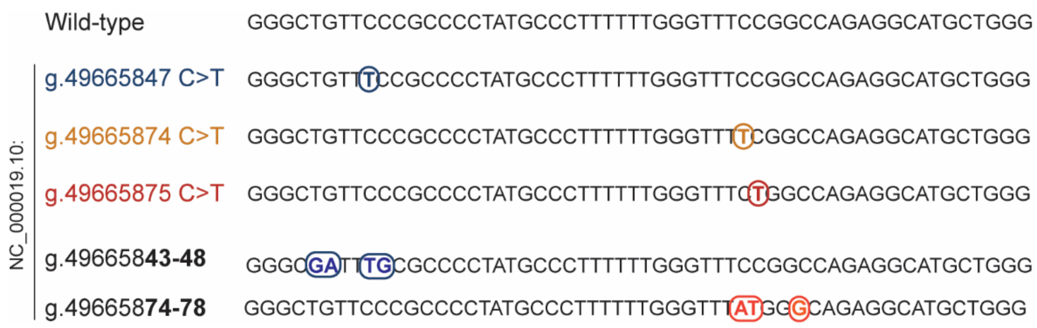

Figure S11. Sequences of *BCL2L12* promoter dual-luciferase assays. The upper four lines indicate the sequences of the wild-type and mutated *BCL2L12* promoter reporter assays to model the *IRF3/BCL2L12* promoter mutations that we identified in melanoma. Results from these reporter assays are shown in Figure 3E. The lowest two lines indicate the sequences of the *BCL2L12* promoter assays shown in Figure 3F. Here, the *BCL2L12* promoter region was mutated to disrupt all predicted transcription factor binding sites in the regions NC_000019.10:g.49665843-48 and NC_000019.10:g.49665874-78.

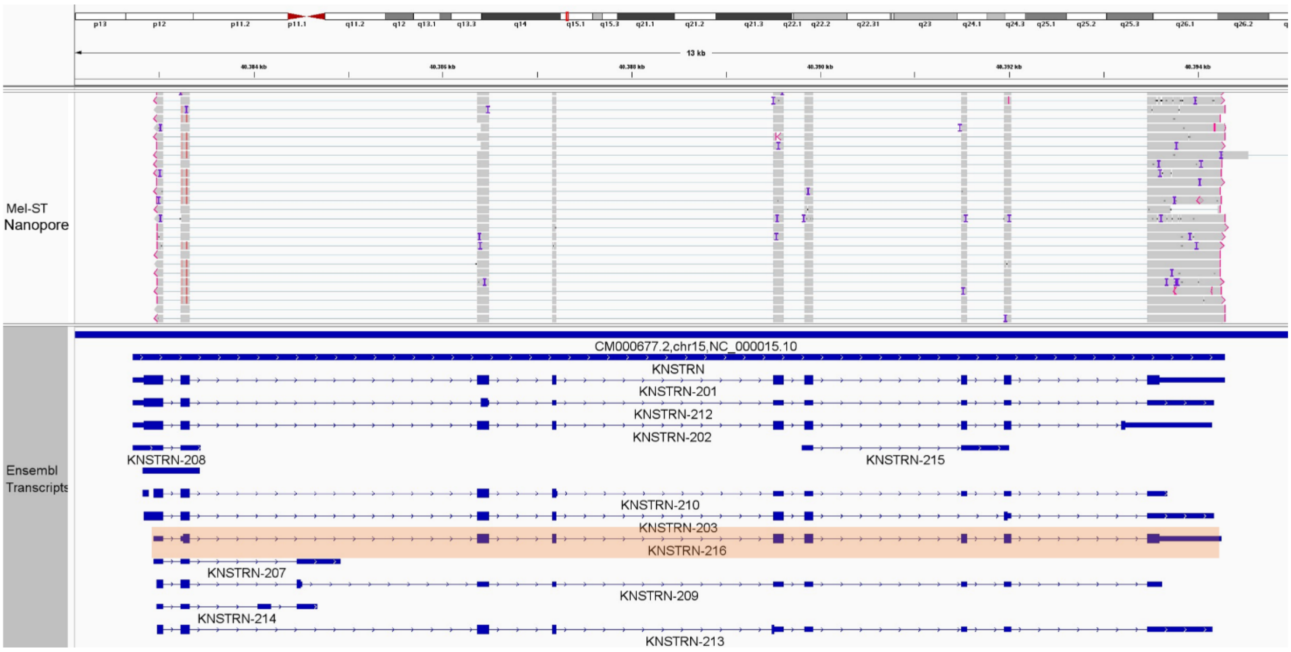

Figure S12. Confirmation of main expressed *KNSTRN* transcript by Oxford Nanopore Technology (ONT) Sequencing on Mel-ST cells. IGV analysis showing mapping of ONT direct cDNA sequencing reads, confirming that KNSTRN-216 (or ENST00000608100) is the main expressed KNSTRN isoform in Mel-ST cells.

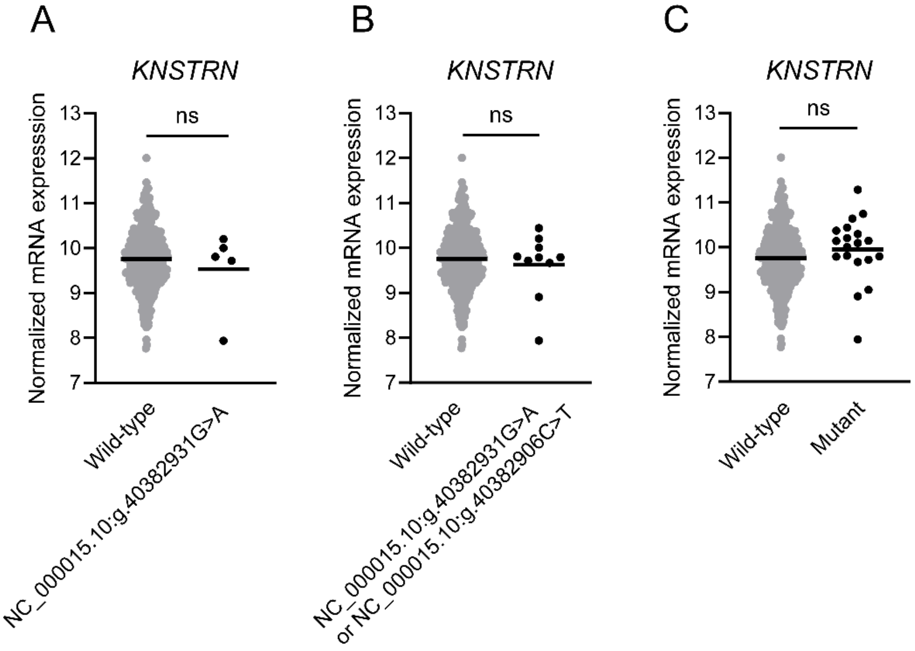

Figure S13. Impact of *KNSTRN* promoter mutations on mRNA expression in melanoma. A) Normalized *KNSTRN* mRNA expression in melanoma tumors with a wild-type (n=421) or NC_000015.10:g.40382931G>A mutant (n=5) *KNSTRN* promoter region. **B)** Normalized *KNSTRN* mRNA expression in melanoma tumors with a wild-type (n=421) or NC_000015.10:g.40382906C>T or NC_000015.10:g.40382931G>A mutant (n=10) *KNSTRN* promoter region. **C)** Normalized *KNSTRN* mRNA expression in melanoma tumors with a wild-type (n=421) or mutant *KNSTRN* promoter region (any mutation in region NC_000015.10:g.40382846-40382938; n=19). Horizontal lines indicate median expression. Statistics: Mann–Whitney U test.

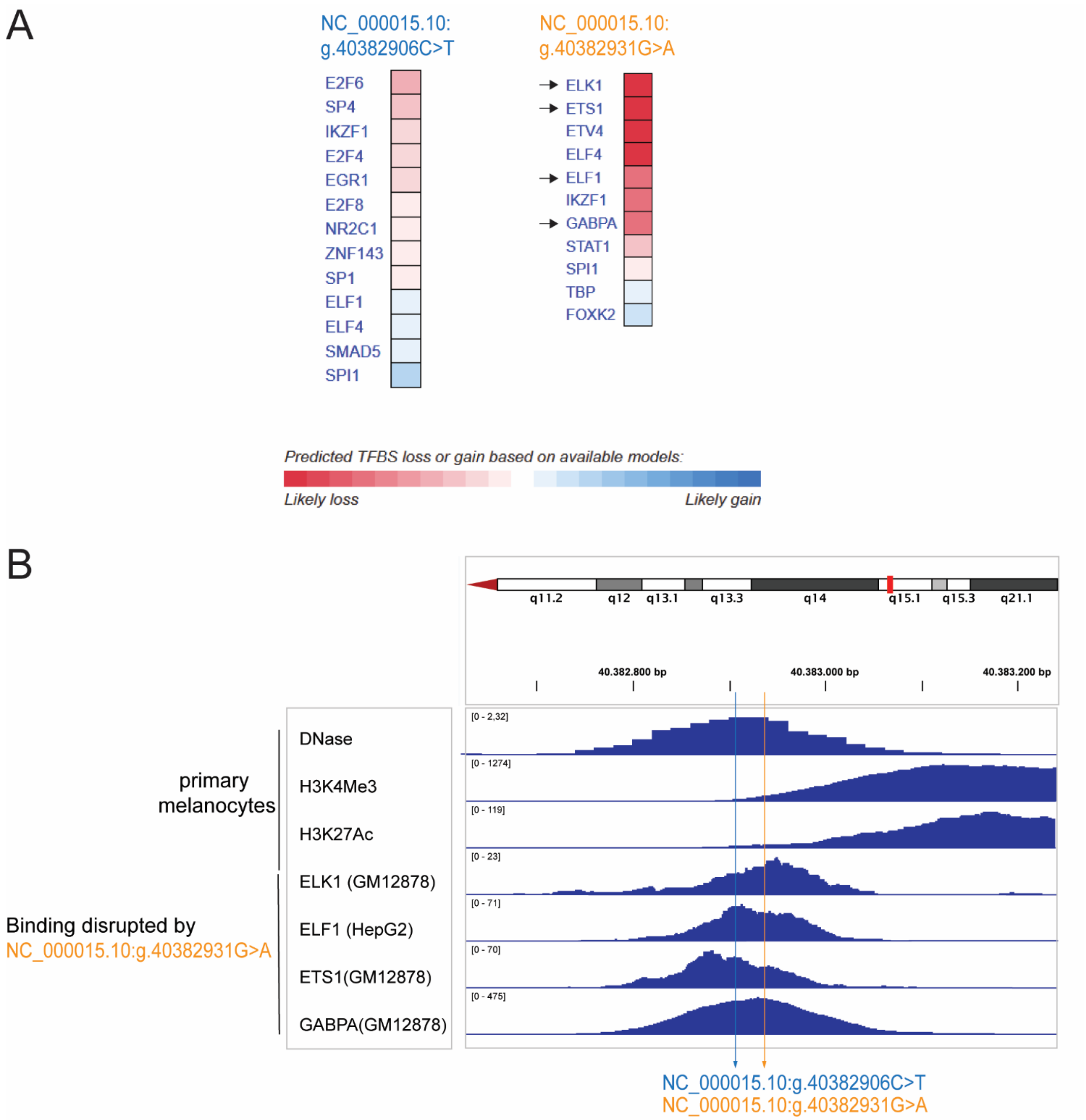

Figure S14. PhysBinder and FABIAN-Variant prediction of the impact of *KNSTRN* promoter mutations on transcription factor binding, and ChIP transcription factor binding of wild-type promoter A) Predicted effect of NC_000015.10:g.40382906C>T and NC_000015.10: g.40382931G>A on transcription factor binding using FABIAN-variant. Results corroborated by PhysBinder are indicated with an arrow. **B)** ENCODE DNase-seq and ChIP-seq data for H3K4Me3 and H3K27Ac show open chromatin and an active KNSTRN promoter region in wild-type primary melanocytes. ENCODE ChIP-seq results supporting binding of the indicated transcription factors to the wild-type *KNSTRN* promoter region in cell lines from which data were available.

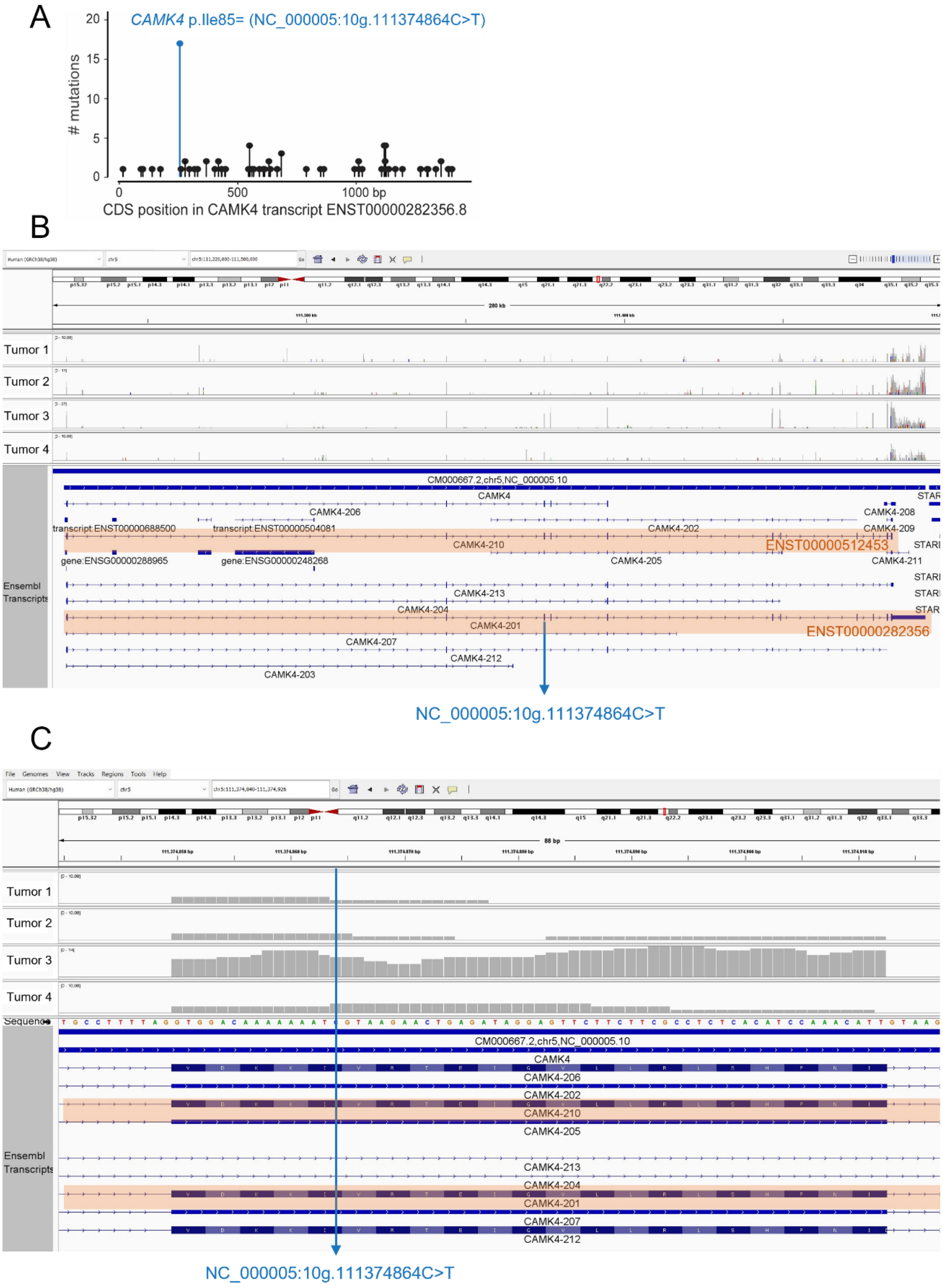

Figure S15. *CAMK4* synonymous mutations. A) Needleplot of mutations in *CAMK4* transcript ENST00000282356.8 in malignant melanoma (based on COSMIC). **B)** IGV analysis showing mapping of RNA-seq reads of four representative SKCM tumors against the *CAMK4* genome locus. The transcripts that are identified by Salmon as main expressed transcripts are indicated in orange (CAMK4-201; ENST00000282356 and CAMK4-210; ENST00000512453). **C)** Blow-up of the image shown in panel A, clearly showing that the position of the NC_000005.10:g.111374864C>T mutation is covered by RNA-seq reads, and that this mutation corresponds to a synonymous mutation in the low expressed *CAMK4* gene.

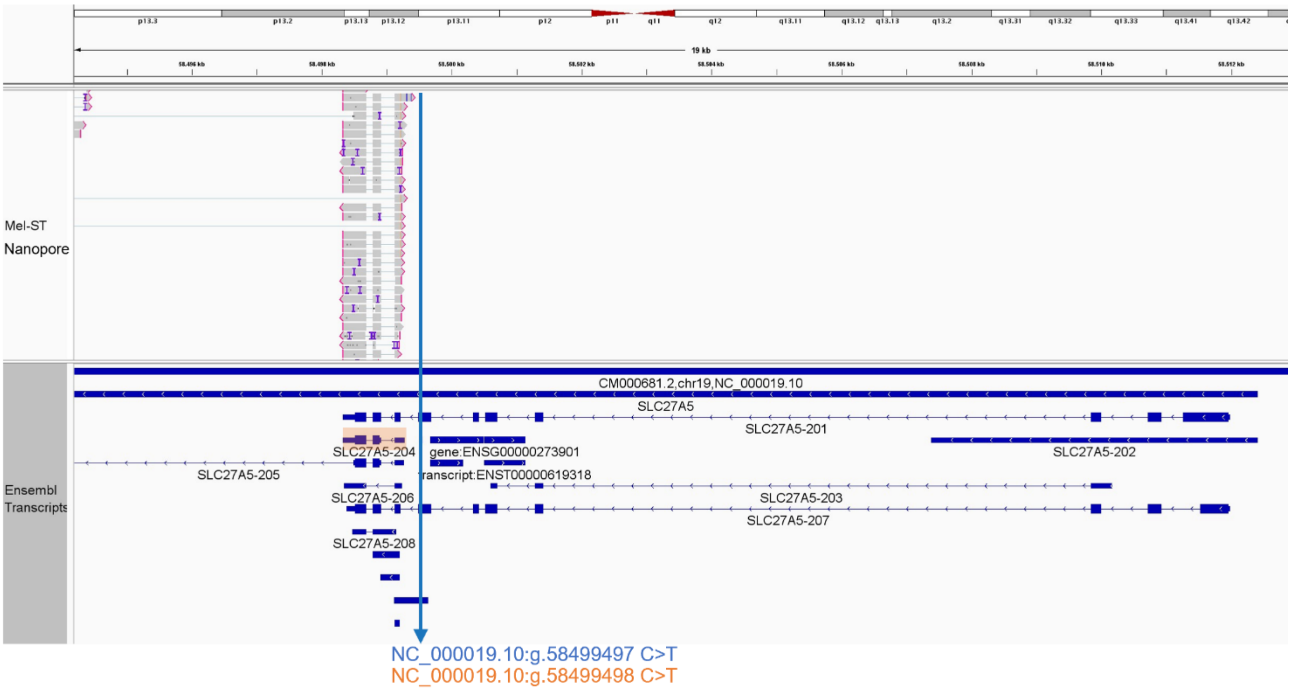

Figure S16. Confirmation of main expressed *SLC27A5* transcript by Oxford Nanopore Technology (ONT) Sequencing on Mel-ST cells. IGV analysis showing mapping of ONT direct cDNA sequencing reads, confirming that SLC27A5-204 (or ENST00000594786) is the main expressed *SLC27A5* isoform in Mel-ST cells. The position of the *SLC27A5* promoter mutations is indicated.

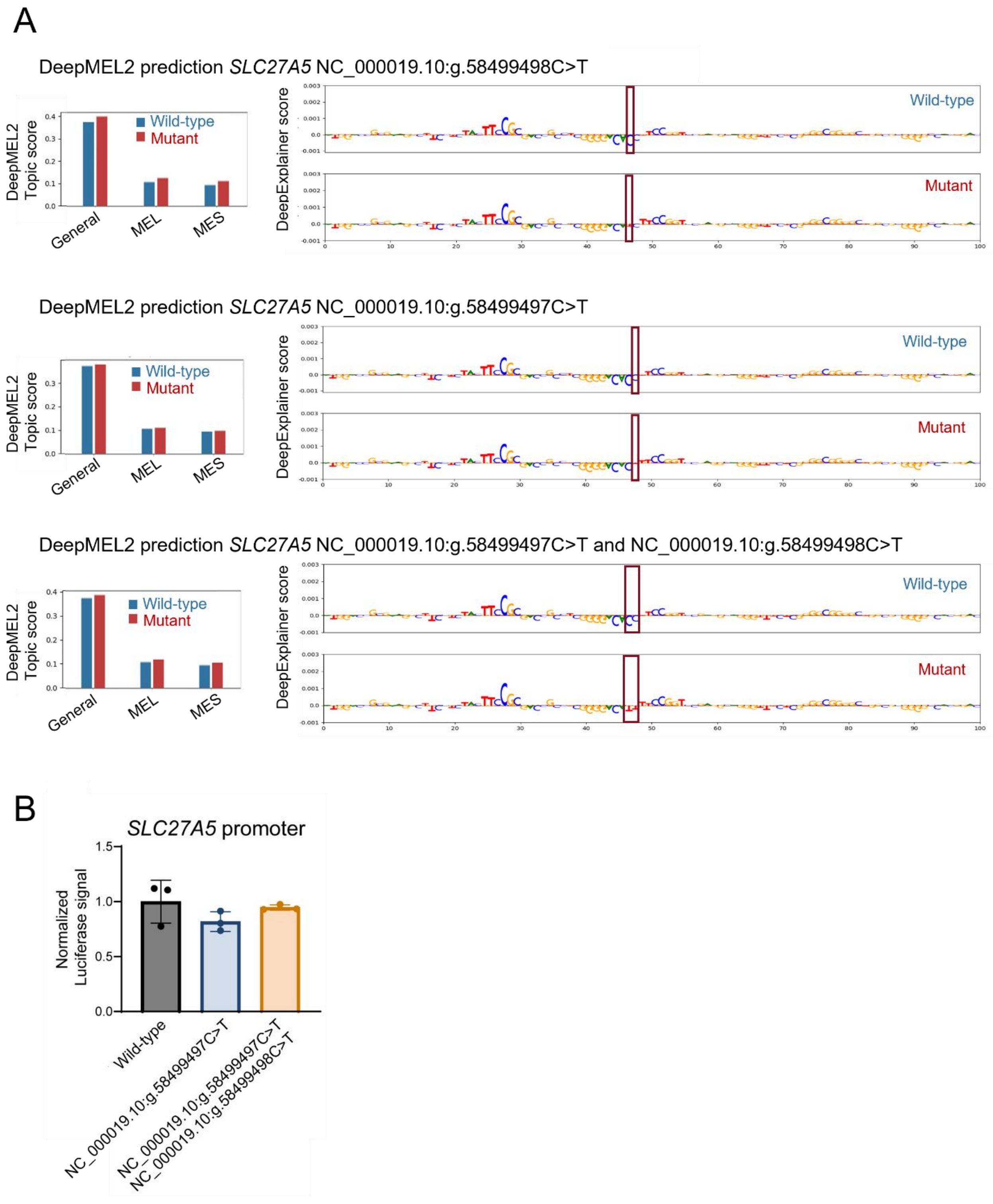

Figure S17. ***SLC27A5*** **DeepMEL2 predictions and reporter assay. A)** Scoring of the *SLC27A5* promoter variants using DeepMEL2. The visualisation on the right shows the nucleotide targeted by the mutation (indicated by a red rectangle), as well as the 50 nucleotides upstream and downstream, and illustrates potential effects on transcription factor binding sites. Note that also the NC_000019.10:g.58499497C>T/NC_000019.10:g.58499498C>T double mutant was analyzed, as we identified several melanoma tumors that display this double mutated genotype**. B)** Ratio of Firefly luciferase signal (under *SLC27A5* promoter) over Renilla luciferase signal (under constitutive TK promoter) as a measurement of wild-type and mutant *SLC27A5* promoter activity using a luciferase assays in HEK293T cells. Error bars indicate standard deviations. Statistics: Ordinary one-way ANOVA.

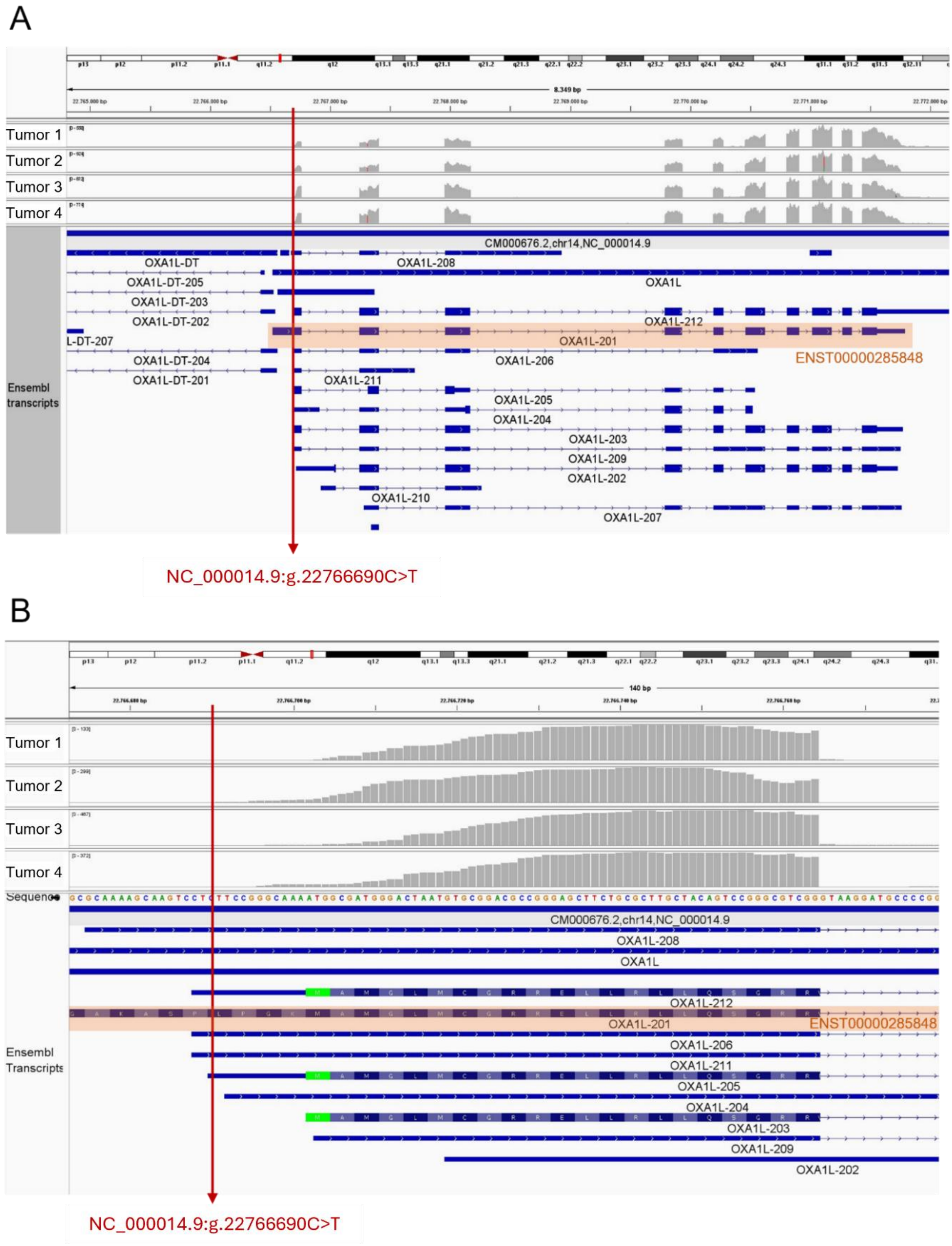

Figure S18. *OXA1L* Upstream non-coding mutations targeting the *OXA1L/OXA1L-DT* promoter region. A) IGV analysis showing mapping of RNA-seq reads of four representative SKCM tumors against the *OXA1L* genome locus. The transcript that is identified by Salmon as main expressed transcript is indicated in orange (OXA1L-201; ENST00000285848). The figure shows that the expression of RNA-seq reads does not entirely match the OXA1L-201 transcript, and that SKCM samples mainly express a transcript that is not reported in Ensembl. Furthermore, the image shows that the NC_000014.9:g.22766690C>T targets the shared promoter region of *OXA1L* and *OXA1L-DT*. **B)** Blow-up of the image in panel A, clearly showing that the position of the NC_000014.9:g.22766690C>T mutation is not covered by RNA-seq reads, and that this mutation is an upstream non-coding gene promoter mutation.

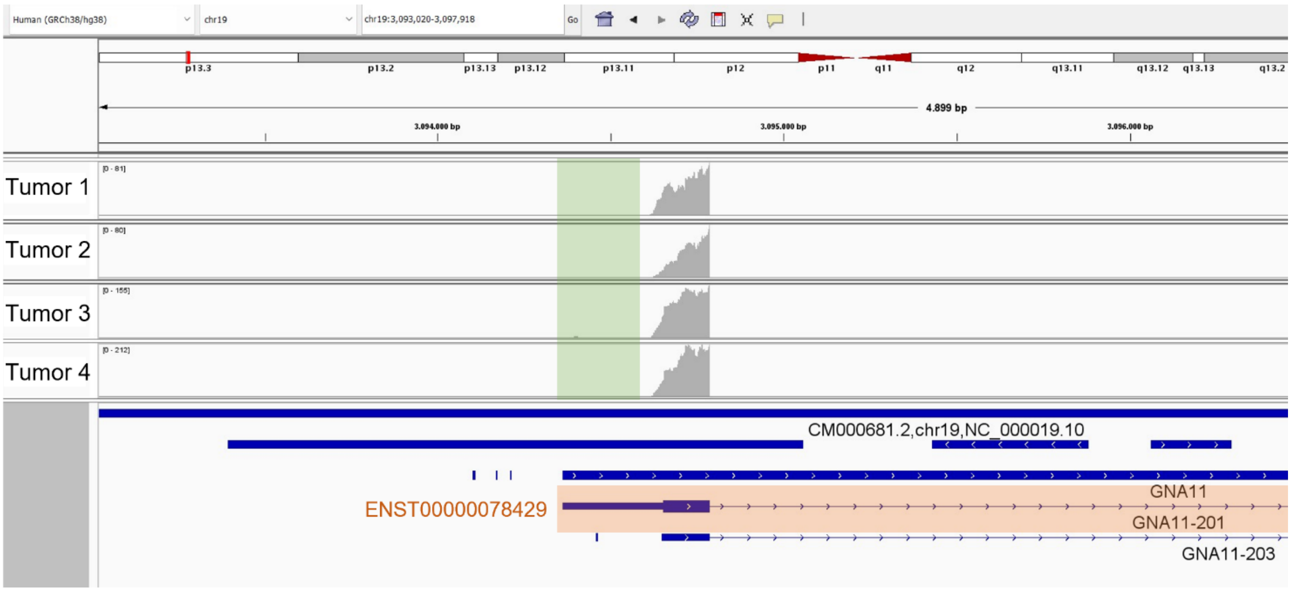

Figure S19. *GNA11* expressed transcript in SKCM tumors has a different 5′ UTR. IGV analysis showing mapping of RNA-seq reads of four representative SKCM tumors against the *GNA11* genome locus. The transcript that is identified by Salmon as main expressed transcript is indicated in orange (GNA11-201; ENST00000078429). The figure shows that the expression of RNA-seq reads does not entirely cover the GNA11-201 transcript, and that SKCM samples a transcript with a different UTR that is not reported in Ensembl. The green area indicates a 5′ UTR region in transcript GNA11-201 that is not covered by RNA-seq reads in SKCM tumors.

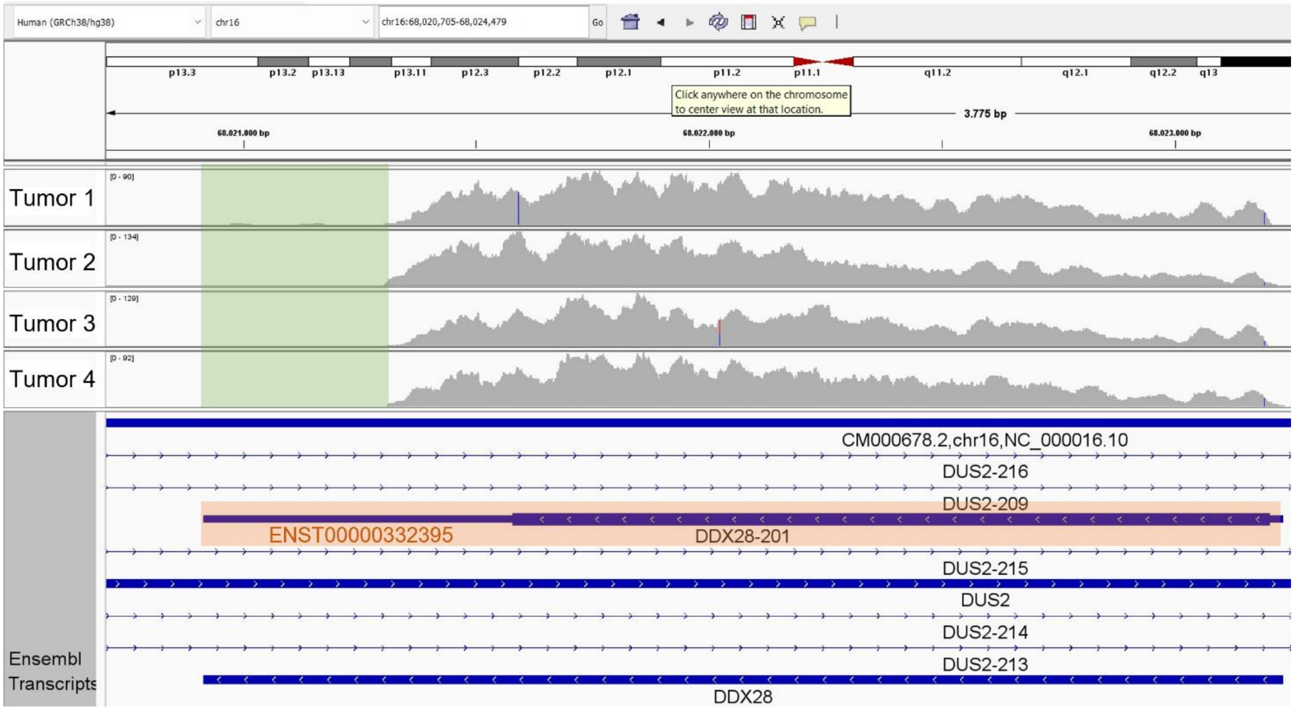

Figure S20. *DDX28* expressed transcript in SKCM tumors has a different 3′ UTR. IGV analysis showing mapping of RNA-seq reads of four representative SKCM tumors against the *DDX28* genome locus. The transcript that is identified by Salmon as main expressed transcript is indicated in orange (DDX28-201; ENST00000332395). The figure shows that the expression of RNA-seq reads does not entirely cover the DDX28-201 transcript, and that SKCM samples express a transcript with a different UTR that is not reported in Ensembl. The green area indicates a 3′ UTR region in transcript DDX28-201 that is not covered by RNA-seq reads in SKCM tumors.

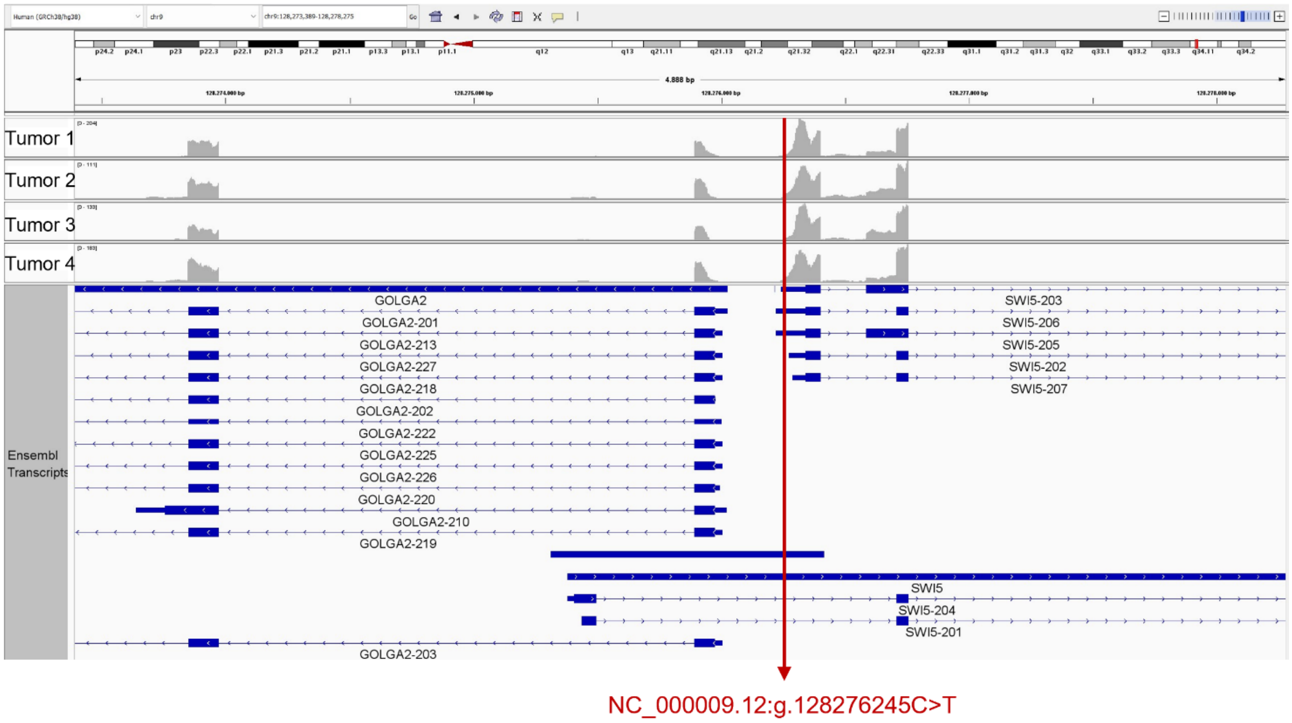

Figure S21. *SWI5/GOLGA2* promoter mutations. IGV analysis showing mapping of RNA-seq reads of four representative SKCM tumors against the *SWI5* genome locus. The image shows that the NC_000009.12:g.128276245 C>T mutation targets the *SWI5/GOLGA2* promoter region.

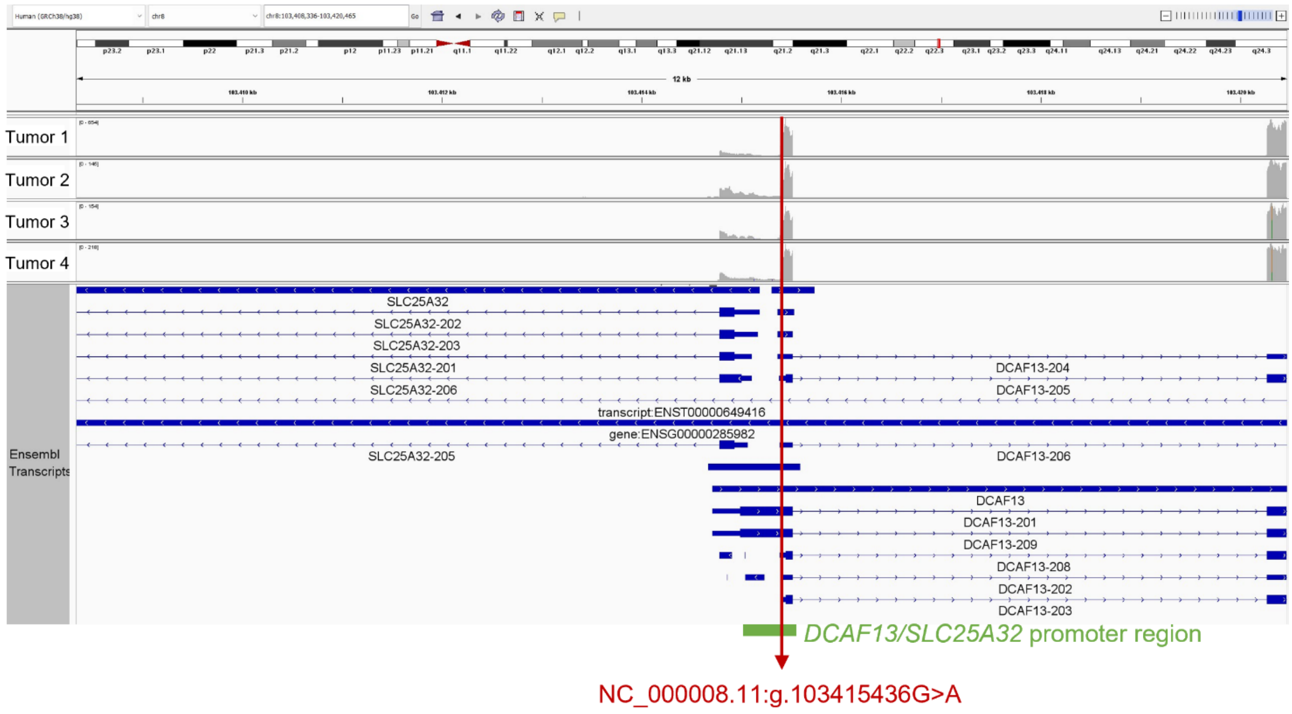

Figure S22. *DCAF13/SLC25A32* promoter mutations. IGV analysis showing mapping of RNA-seq reads of four representative SKCM tumors against the *DCAF13* genome locus. The image shows that the NC_000008.11g.103415436G>A mutation targets the shared *DCAF13/SLC25A32* promoter region.

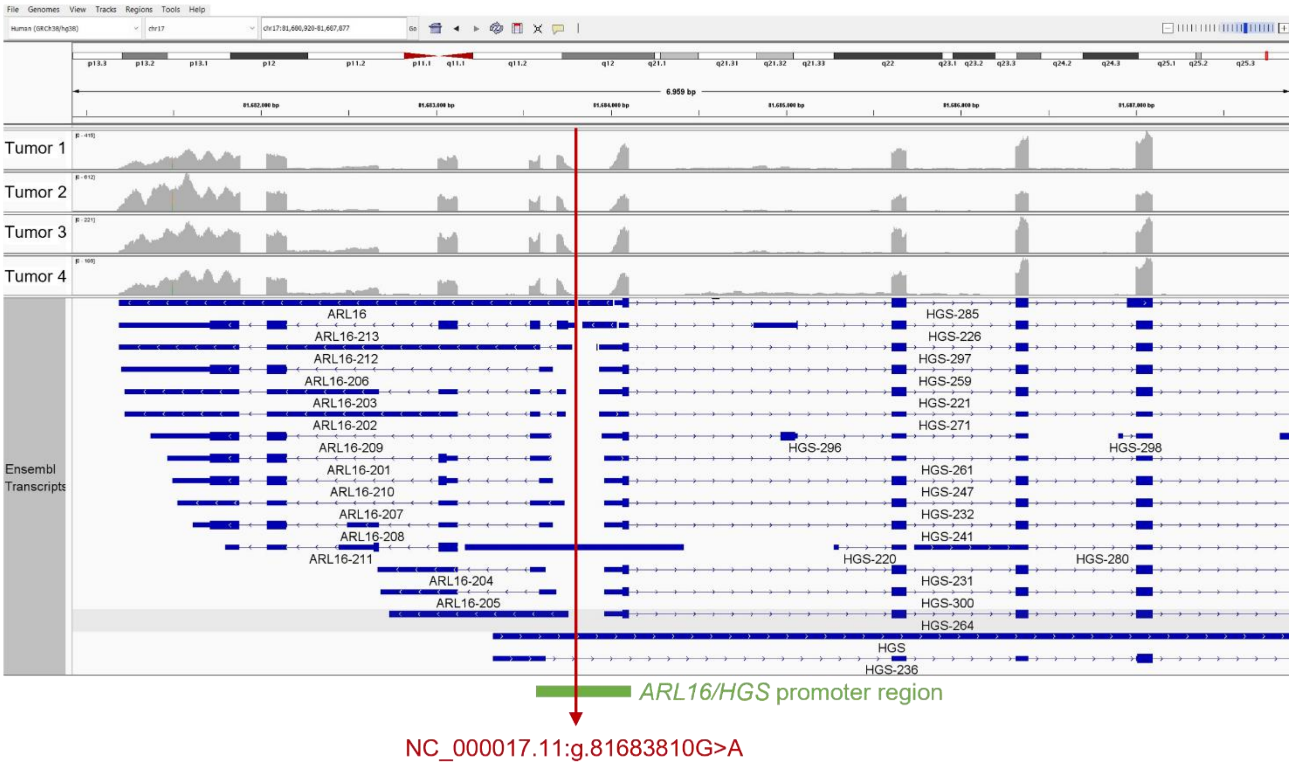

Figure S23. *ARL16/HGS* promoter mutations. IGV analysis showing mapping of RNA-seq reads of four representative SKCM tumors against the *ARL16* genome locus. The image shows that the NC_000017.11:g.81683810G>A mutation targets the shared *ARL16/HGS* promoter region.

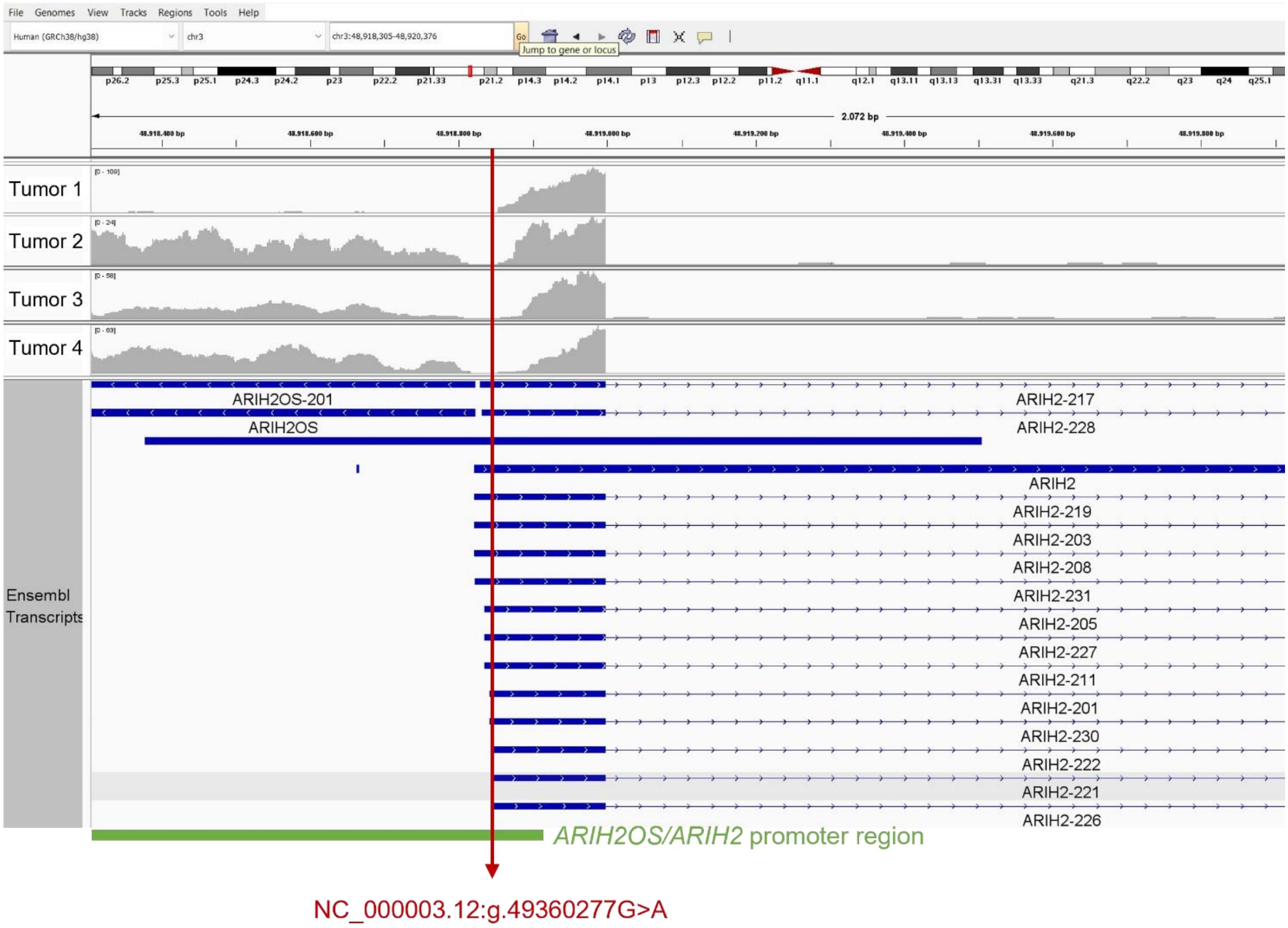

Figure S24. *ARIH2OS/ARIH2* promoter mutations. IGV analysis showing mapping of RNA-seq reads of four representative SKCM tumors against the *ARIH2OS* genome locus. The image shows that the NC_000003.12:g.48918840G>A mutation targets the shared *ARIH2OS/ARIH2* promoter region.

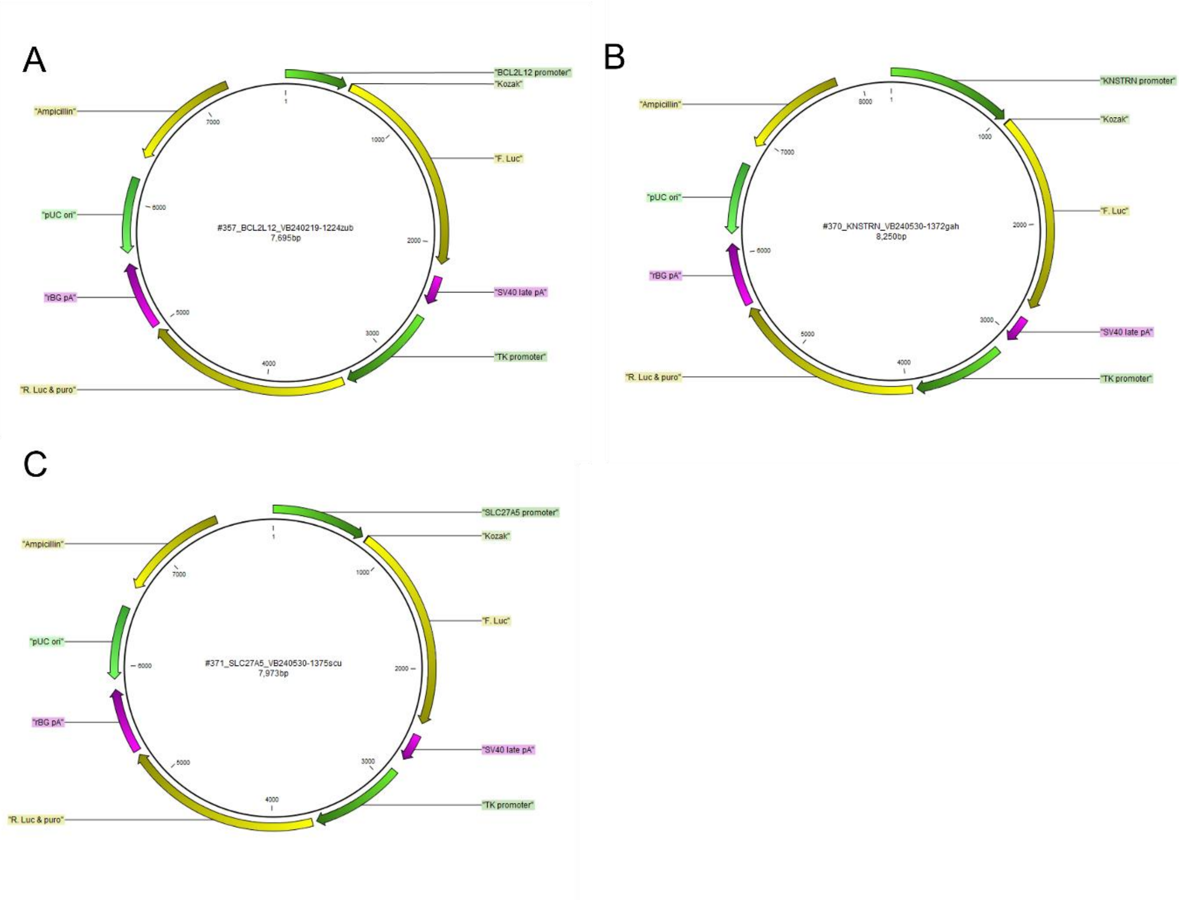

Figure S25. Plasmid maps of dual-luciferase reporter plasmids with wild-type and mutated promoter regions for A) *BCL2L12* (NC_000019.10:g.49665486 – 49665965) **B)** *KNSTRN* (NC_000015.10:g.403822312 – 40383265) and **C)** *SLC27A5* (NC_000019.10:g.58499132 – 58499889).

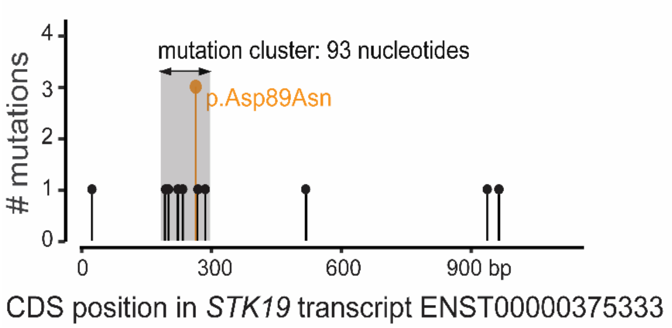

Figure S26. *WHR1* (*STK19*) mutation cluster. Needleplot of mutations in *WHR1* (formerly known as *STK19*) in the SKCM MC3 dataset in respect to transcript ENST00000375333.

Tumor type	Tumor type (complete name)	# Analyzed Samples
BLCA	Bladder Urothelial Carcinoma	402
BRCA	Breast invasive carcinoma	755
CESC	Cervical squamous cell carcinoma and endocervical adenocarcinoma	270
COADREAD	Colon adenocarcinoma and rectum adenocarcinoma	315
HNSC	Head and Neck squamous cell carcinoma	482
KIRC	Kidney renal clear cell carcinoma	366
KIRP	Kidney renal papillary cell carcinoma	281
LGGGBM	Low-grade glioma and glioblastoma multiforme	816
LIHC	Liver hepatocellular carcinoma	353
LUAD	Lung adenocarcinoma	503
LUSC	Lung squamous cell carcinoma	466
PRAD	Prostate adenocarcinoma	484
THCA	Thyroid carcinoma	483
UCEC	Uterine Corpus Endometrial Carcinoma	401
SKCM	Skin Cutaneous Melanoma	449
STES	Stomach and Esophageal carcinoma	486
SARC	Sarcoma	228

Table S1 - Analyzed tumor types. Overview of the analyzed tumor types in the MC3-working group dataset that were used in our study. The right column reports the number of samples that were included in our analyses after filtering in the data pre-processing steps.

BCL2L12 Transcripts				Location of mutations relative to *BCL2L12* transcript		Remark
Ensembl_Transcript ID	NCBI_ID	UCSC	Protein size (kDa)	NC_000019.10:g. 49665847 (hg38)	NC_000019.10:g. 49665874/75 (hg38)	
ENST00000594157.5	NM_001282517.2	NM_001282517	9.2	Upstream non-coding	Upstream non-coding	
-	NM_001282519.2	NM_001282519	19.9	Upstream non-coding	Upstream non-coding	
ENST00000246784.8	NM_138639.2	NM_138639	26.8	Upstream non-coding	Upstream non-coding	
-	NM_001282516.2	NM_001282516	17.1	Upstream non-coding	Upstream non-coding	
-	NM_001282521.2	NM_001282521	11.4	Upstream non-coding	Upstream non-coding	
-	NM_0010040668.2	NM_0010040668	26.8	Upstream non-coding	Upstream non-coding	
-	NM_001385706.1	NM_001385706	16.9	Upstream non-coding	Upstream non-coding	
ENST00000598979.5	-	-	9.2	Upstream non-coding	Upstream non-coding	
ENST00000600947.5	-	-	14.7	Upstream non-coding	Upstream non-coding	
ENST00000598306.2	-	-	10	Upstream non-coding	Upstream non-coding	
ENST00000594793.1	-	-	-	Upstream non-coding	Upstream non-coding	Transcript is not protein coding
ENST00000601168.1	-	-	-	Upstream non-coding	Upstream non-coding	Transcript is not protein coding
ENST00000246785.7	-	-	36.8	5'UTR	5'UTR	
ENST00000698560.1	-	-	9.2	5'UTR	5'UTR	
ENST00000619007.4	-	-	20	5'UTR	5'UTR	
ENST00000698555.1	-	-	20	5'UTR	5'UTR	
ENST00000698554.1	-	-	17.2	5'UTR	5'UTR	
ENST00000441864.6	-	-	36.9	5'UTR	5'UTR	
ENST00000698557.1	-	-	17.1	5'UTR	5'UTR	
-	NR_104201.1	NR_104201	-	5'UTR	5'UTR	Transcript is not protein coding
-	NR_104202.1	NR_104202	-	5'UTR	5'UTR	Transcript is not protein coding
-	NR_104203.1	NR_104203	-	5'UTR	5'UTR	Transcript is not protein coding
-	NR_104200.1	NR_104200	-	5'UTR	5'UTR	Transcript is not protein coding
-	NR_104204.1	NR_104204	-	5'UTR	5'UTR	Transcript is not protein coding
-	NR_104205.1	NR_104205	-	5'UTR	5'UTR	Transcript is not protein coding
ENST00000698558.1	-	-	19.8	5'UTR	5'UTR	
ENST00000698553.1	-	-	14.7	5'UTR	5'UTR	

ENST00000698556.1	-	-	14.5	5'UTR	5'UTR	
ENST00000698559.1	-	-	8	5'UTR	5'UTR	
ENST00000698561.1	-	-	5.1	5'UTR	5'UTR	
-	NM_001282520.1	NM_001282520	24.5	Protein coding (F8)	Protein coding (F17/R18)	

Table S7- *BCL2L12* isoforms. Overview of *BCL2L12* isoforms described in Ensembl, UCSC and NCBI. The table also reports the encoded proteins (if the isoform is protein coding) and the position of the nucleotides targeted by mutations in SKCM in respect to each of the reported transcripts.

	SKCM tumors (Illumina seq)		Mel-ST (Oxford Nanopore Technologies) (FLAIR isoform analysis, followed by manual correction in IGV)	Main expressed SKCM consensus transcript
Gene	Salmon	IGV		
BRAF	ENST00000496384	ENST00000496384	ENST00000496384.7	ENST00000496384
NRAS	ENST00000369535	ENST00000369535	ENST00000369535.5	ENST00000369535
CAMK4	ENST00000282356	ENST00000282356	ENST00000282356.9	ENST00000282356
SLC27A5	ENST00000594786	ENST00000594786	ENST00000594786.1	ENST00000594786
BCL2L12	ENST00000246784	ENST00000246784	ENST00000246784.8	ENST00000246784
KNSTRN	ENST00000608100	ENST00000608100	ENST00000608100.5	ENST00000608100
IDH1	ENST00000345146	ENST00000345146	ENST00000345146.7	ENST00000345146
BAAT	ENST00000259407	ENST00000259407	Not expressed	ENST00000259407
ANKRD30B	ENST00000320584	Too low expressed to evaluate	Too low expressed to evaluate	Too low expressed to evaluate
SLC9A9	ENST00000316549	ENST00000316549	ENST00000316549.11	ENST00000316549
OASL	ENST00000257570	ENST00000679655	ENST00000679655	ENST00000679655
TACC3	ENST00000313288	ENST00000313288	ENST00000313288.9	ENST00000313288
RAC1	ENST00000348035	ENST00000348035	ENST00000348035.9	ENST00000348035
PTGER3	ENST00000370924	ENST00000370924	Not expressed	ENST00000370924
IL17RD	ENST00000296318	ENST00000296318	ENST00000296318.12	ENST00000296318
CCDC28A	ENST00000617445	ENST00000617445	ENST00000617445.5	ENST00000617445
ZNF28	ENST00000457749	ENST00000457749	Too low expressed to evaluate	ENST00000457749
NPAS1	ENST00000439365	ENST00000439365	ENST00000439365.6	ENST00000439365
CNOT9;RQCD1	ENST00000273064	ENST00000273064	ENST00000273064.11-1	ENST00000273064
POLA2	ENST00000265465	ENST00000706538	ENST00000706538.1	ENST00000706538
GNAI2	ENST00000313601	ENST00000313601	ENST00000313601.11	ENST00000313601
FAM13A	ENST00000264344	ENST00000264344	de novo transcript, highly similar to ENST00000264344.10	ENST00000264344
DCLK2	ENST00000411937	ENST00000506325	Too low expressed to evaluate	ENST00000506325
SMURF2	ENST00000582081	ENST00000262435	ENST00000262435.14	ENST00000262435
RIPPLY3	ENST00000329553	ENST00000329553	Not expressed	ENST00000329553
PLK1	ENST00000300093	ENST00000300093	ENST00000300093.9	ENST00000300093
OXA1L	ENST00000285848	ENST00000285848.9 (but shorter 5'end)	ENST00000285848.9 (but shorter 5'end)	ENST00000285848.9 (but shorter 5'end)

STXBP4	ENST00000376352	ENST00000376352	ENST00000376352.6	ENST00000376352
GNA11	ENST00000078429	ENST00000078429 (but shorter 5'UTR)	ENST00000078429.9 (but shorter 5'UTR)	ENST00000078429 (but shorter 5'UTR)
DDX41	ENST00000330503	ENST00000330503	ENST00000330503.12	ENST00000330503
ATP2B1	ENST00000635033	ENST00000428670	ENST00000428670.8	ENST00000428670
TREM1	ENST00000589695	ENST00000244709	Not expressed	ENST00000244709
TBCK	ENST00000361687	ENST00000361687	ENST00000361687	ENST00000361687
GPCPD1	ENST00000379019	ENST00000379019	ENST00000379019.7	ENST00000379019
CDK4	ENST00000257904	ENST00000257904	ENST00000257904.11	ENST00000257904
AKR1B1	ENST00000285930	ENST00000285930	ENST00000285930.9	ENST00000285930
ZNF329	ENST00000358067	ENST00000358067	ENST00000598312.6	ENST00000358067
SWI5	ENST00000418976	ENST00000418976	ENST00000418976.3	ENST00000418976
RHOA	ENST00000418115	ENST00000418115	ENST00000418115.6	ENST00000418115
DHX15	ENST00000336812	ENST00000336812	ENST00000336812.5	ENST00000336812
DCAF13	ENST00000612750	ENST00000612750	ENST00000612750.5	ENST00000612750
ARL16	ENST00000622299	ENST00000622299	ENST00000622299.5	ENST00000622299
AIFM1	ENST00000287295	ENST00000287295	ENST00000287295.8	ENST00000287295
ZBTB12	ENST00000375527	ENST00000375527	ENST00000375527.3	ENST00000375527
YIPF1	ENST00000464950	ENST00000464950	ENST00000464950.6	ENST00000464950
TDRD7	ENST00000355295	ENST00000355295	ENST00000355295.5	ENST00000355295
MGAM2;RP11-1220K2.2	ENST00000477922	Too low expressed to evaluate	not expressed	Too low expressed to evaluate
PPP1R1A	ENST00000257905	ENST00000257905	not expressed	ENST00000257905
ARIH2OS	ENST00000647812	ENST00000647812	ENST00000647812.2	ENST00000647812
TMEM154	ENST00000304385	ENST00000304385	ENST00000304385.8 (but shorter 5'UTR)	ENST00000304385
SLC39A6	ENST00000269187	ENST00000269187	ENST00000269187.10	ENST00000269187
DDX28	ENST00000332395	ENST00000332395 (but shorter 3' UTR)	ENST00000332395.7 (but shorter 3' UTR)	ENST00000332395 (but shorter 3' UTR)

Table S8- Main expressed isoform using Illumina and long-read ONT. The table reports the main expressed isoform in SKCM tumors for the indicated genes identified by Salmon or by verification in IGV based on Illumina sequencing data. In addition, the table reports the main expressed isoform identified by long-read ONT sequencing in Mel-ST cells. This isoform was determined using the FLAIR analysis pipeline, followed by manual verification and correction in IGV where needed. *De novo* transcripts identified by FLAIR were only considered when no known GENCODE v47 transcript was identified. The column on the right reports the main expressed SKCM consensus transcript based on the Illumina and ONT sequencing isoform analysis.

Gene	P-value	Fold change
OASL	0.11	0.49
FAM13A	0.01	0.35
KNSTRN	0.38	1.10
NPAS1	0.32	0.63
STXBP4	0.41	1.22
CCDC28A	0.74	1.05
BCL2L12	0.07	0.80
TREM1	0.30	0.51
TBCK	0.52	0.84
POLA2	NA	NA
ZNF28	0.69	1.17
SLC9A9	0.42	0.69
ATP2B1	0.32	1.56
CAMK4	0.14	0.54
ZBTB12	NA	NA
AIFM1	0.94	1.02
BAAT	0.00	0.01
AKR1B1	0.23	1.50
PPP1R1A	0.00	0.05
DDX28	0.03	0.57
PLK1	0.88	0.95
BRAF	0.00	1.27
NRAS	0.00	1.58
IL17RD	0.97	0.98
TACC3	0.12	0.67
IDH1	0.04	1.30
YIPF1	0.98	1.00
RAC1	0.37	1.08
ARIH2OS	0.79	1.07
SWI5	0.76	0.93
RIPPLY3	0.49	1.69
SLC27A5	0.63	1.11
GPCPD1	0.82	0.93
CNOT9	0.55	1.07
ZNF329	0.22	0.68
SMURF2	0.26	0.76
DCLK2	0.36	1.35
OXA1L	0.07	1.34
DHX15	0.46	1.14
PTGER3	0.32	0.62
TMEM154	0.03	0.23
ARL16	0.45	1.12
SLC39A6	0.57	0.84
GNAI2	0.87	1.04
CDK4	0.06	0.66
DCAF13	NA	NA
DDX41	0.84	0.95

TDRD7	NA	NA
RHOA	NA	NA
PTGER3	NA	NA
GNA11	0.14	0.76

Table S9- Impact of SKCM mutation clusters on RNA expression of their host gene. Supportive data of Figure 6. The table reports the p-values and fold changes of differential RNA expression of the indicated genes in SKCM tumors with a mutated status for the identified mutation cluster in that gene (mutation cluster defined here as the region ranging from 30 nucleotides upstream to 30 nucleotides downstream of the most recurrent mutation) as compared to tumors with a wild-type status for the gene of interest.

RT-qPCR primers	
Gene	Primer sequence (5'-3')
	TGCACCACCAACTGCTTAGC
GAPDH	GGCATGGACTGTGGTCATGAG
	AGACACGCTGAGGGTCCTA
BCL2L12	TGGAGTTGGAACAGGAGACC
	TCGTGATGGTCAAGGTTGTG
IRF3	ATGTGCAGGTCCACAGTATTC
	CCTCAGCATCTTATCCGAGTGG
TP53	TGGATGGTGGTACAGTCAGAGC
	AGGTGGACCTGGAGACTCTCAG
CDKN1A	TCCTCTTGGAGAAGATCAGCCG
BCL2L12 shRNA SEQUENCES	
Name	Primer sequence (5'-3')
shRNA_A	GGTCTCCTGTTCCAACTCCACCTAGAAGC
shRNA_B	GCTACTCCAGACTTCTATGCTTTGGTGGC
shRNA_C	AAGAGCCAACAGACTTCCTGAGCCGCCTT
shRNA_D	ACAGAGAAGGAAGCCATACTGCGGAGGCT

Table S10- Materials and methods. Overview of RT-qPCR primers and shRNA sequences.

ANTIBODIES USED FOR WESTERN BLOTTING			
Target	Company	CAT#	Dilution
Vinculin	Sigma-Aldrich	V9131	1:25000
BCL2L12	ProteinTech	21969-1-AP	1:1000
IRF3	Invitrogen	703682	1:1000
TP53	Cell Signalling Technology	2524	1:1000
CDKN1A	Cell Signalling Technology	2947	1:1000
Goat Anti-Mouse IgG (H+L)	Thermo Fisher	31432	1:5000
Goat Anti-Rabbit IgG (H+L)	Thermo Fisher	31462	1:5000

Table S11- Materials and methods. Overview antibodies used for western blotting.

Legends of Supplemental tables provided as separate Excel files.

Table S2- Results concentration area method. Table reporting results obtained for the concentration area method that was applied on the pre-processed MC3-working group variant list. Results obtained when considering non-synonymous mutations (nSMs), synonymous mutations (SMs) and non-synonymous and synonymous mutations together (All) are reported in different tabs in the Excel file. Columns O-U in the sheet always report on the most frequent mutation in the identified mutation cluster. Note that mutation positions are indicated in respect to hg19, as in the MC3-working group dataset.

Table S3- Results hotspot 12 method. Table reporting results obtained for the hotspot 12 method that was applied on the pre-processed MC3-working group variant list. Results obtained when considering non-synonymous mutations (nSMs), synonymous mutations (SMs) and non-synonymous and synonymous mutations together (All) are reported in different tabs in the Excel file. Columns O-U in the sheet always report on the most frequent mutation in the identified mutation cluster. Note that mutation positions are indicated in respect to hg19, as in the MC3-working group dataset.

Table S4- Results hotspot 3 method. Table reporting results obtained for the hotspot 3 method that was applied on the pre-processed MC3-working group variant list. Results obtained when considering non-synonymous mutations (nSMs), synonymous mutations (SMs) and non-synonymous and synonymous mutations together (All) are reported in different tabs in the Excel file. Note that mutation positions are indicated in respect to hg19, as in the MC3-working group dataset.

Table S5- Results entropy method. Table reporting results obtained for the entropy method that was applied on the pre-processed MC3-working group variant list. Results obtained when considering non-synonymous mutations (nSMs), synonymous mutations (SMs) and non-synonymous and synonymous mutations together (All) are reported in different tabs in the Excel file. Columns I-O in the sheet always report on the most frequent mutation in the gene. Note that mutation positions are indicated in respect to hg19, as in the MC3-working group dataset.

Table S6- Concentration results heatmaps. Table reporting mutations in the genes depicted in the heatmaps in Figure 1B and Figure S2.

Supplemental references

1. Patro, R., Duggal, G., Love, M.I., Irizarry, R.A., and Kingsford, C. (2017). Salmon provides fast and bias-aware quantification of transcript expression. Nature methods *14*, 417-419. 10.1038/nmeth.4197.
2. Atak, Z.K., Taskiran, I.I., Demeulemeester, J., Flerin, C., Mauduit, D., Minnoye, L., Hulselmans, G., Christiaens, V., Ghanem, G.-E., Wouters, J., and Aerts, S. (2021). Interpretation of allele-specific chromatin accessibility using cell state-aware deep learning. Genome Res. *31*, 1082-1096. 10.1101/gr.260851.120.
3. Steinhaus, R., Robinson, P.N., and Seelow, D. (2022). FABIAN-variant: predicting the effects of DNA variants on transcription factor binding. Nucleic Acids Res. *50*, W322-W329. 10.1093/nar/gkac393.
4. Broos, S., Soete, A., Hooghe, B., Moran, R., van Roy, F., and De Bleser, P. (2013). PhysBinder: improving the prediction of transcription factor binding sites by flexible inclusion of biophysical properties. Nucleic Acids Res. *41*, W531-W534. 10.1093/nar/gkt288.
